# Targeting the *Schistosoma mansoni* nutritional mechanisms to design new antischistosomal compounds

**DOI:** 10.1038/s41598-023-46959-3

**Published:** 2023-11-13

**Authors:** Thaís F. A. Pavani, Maria E. Cirino, Thainá R. Teixeira, Josué de Moraes, Daniela G. G. Rando

**Affiliations:** 1 Grupo de Pesquisas Químico-Farmacêuticas, GPQFfesp, Departamento de Ciências Farmacêuticas, Instituto de Ciências Ambientais, Químicas e Farmacêuticas, Universidade Federal de São Paulo (UNIFESP), Rua São Nicolau, 210, 2° Andar, Centro, Diadema, São Paulo 09913-030 Brazil; 2 Instituto de Ciências Ambientais, Químicas e Farmacêuticas, Curso de Pós-Graduação em Biologia Química da Universidade Federal de São Paulo, Diadema, SP Brazil; 3 Núcleo de Pesquisas em Doenças Negligenciadas, NPDN, Universidade Guarulhos, Guarulhos, SP Brazil

**Keywords:** Chemical biology, Drug discovery, Parasitology, Medicinal chemistry

## Abstract

The chemical classes of semicarbazones, thiosemicarbazones, and hydrazones are present in various compounds, each demonstrating diverse biological activities. Extensive studies have revealed their potential as schistosomicidal agents. Thiosemicarbazones, in particular, have shown inhibitory effects on *Schistosoma mansoni's* cathepsin B1 enzyme (*Sm*CB1), which plays a crucial role in hemoglobin degradation within the worm's gut and its nutrition processes. Consequently, *Sm*CB1 has emerged as a promising target for novel schistosomiasis therapies. Moreover, chloroquinoline exhibits characteristics in its aromatic structure that hold promise for developing *Sm*CB1 inhibitors, along with its interaction with hemoglobin's heme group, potentially synergizing against the parasite's gut. In this context, we report the synthesis of 22 hybrid analogs combining hydrazones and quinolines, evaluated against *S. mansoni*. Five of these hybrids demonstrated schistosomicidal activity in vitro, with GPQF-8Q10 being the most effective, causing worm mortality within 24 h at a concentration of 25 µM. GPQF-8Q8 proved to be the most promising in vivo, significantly reducing egg presence in feces (by 52.8%) and immature eggs in intestines (by 45.8%). These compounds exhibited low cytotoxicity in Vero cells and an in in vivo animal model (*Caenorhabditis elegans*), indicating a favorable selectivity index. This suggests their potential for the development of new schistosomiasis therapies. Further studies are needed to uncover specific target mechanisms, but these findings offer a promising starting point.

## Introduction

**Figure 1:**
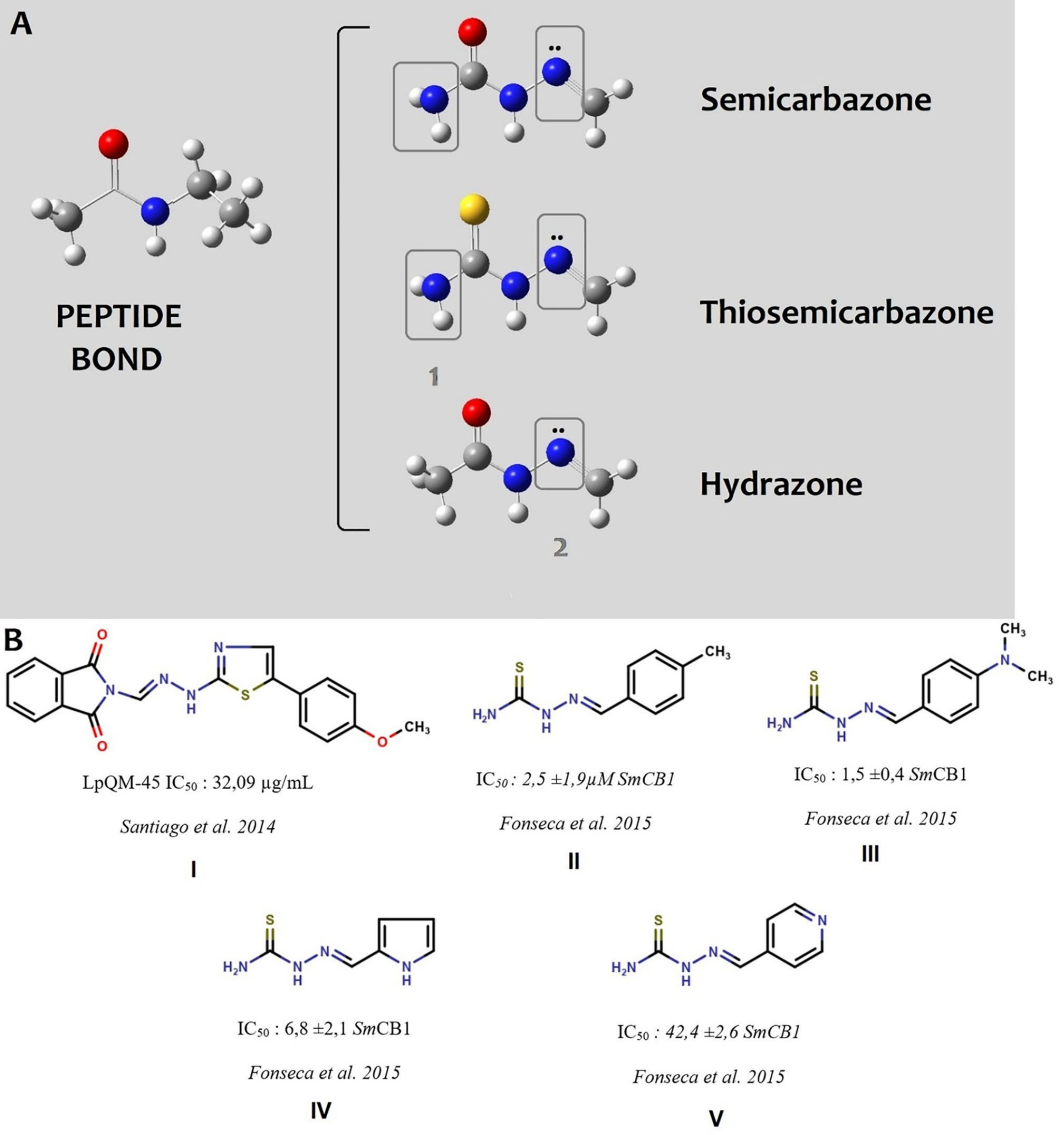
Semicarbazone, thiosemicarbazone, and hydrazone scaffolds and their active representatives against *Schistosoma mansoni* cathepsin B1. (**A**) Semicarbazones, thiosemicarbazones, and hydrazones similarities with peptide bonds and the extra points for potential new interactions highlighted in rectangles (1: hydrogen bond donor/acceptor and 2: hydrogen bond acceptor) with macromolecular targets. Atoms are sketched as ball-sticks and colored following the pattern: blue: nitrogen; red: oxygen; gray: carbon, yellow: sulfur and white: hydrogen. (**B**) *Sm*CB1 inhibitors from the chemical classes of the semicarbazone and thiosemicarbazones described in the literature.

Semicarbazones, thiosemicarbazones, and hydrazones are chemical classes of compounds with a broad range of biological activities. Due to their resemblance to the peptide bonds in proteins, they are regarded as privileged chemical groups, since they mislead macromolecules that initially identify peptide connections. Moreover, they present additional points for intermolecular interaction, which allows them to have greater affinity for the target (Fig. 1A).

In 2014, Santiago et al.^1^ conducted in vitro studies with ten thiosemicarbazones and hydrazones. They selected an analog, the LpQM-45, which significantly reduced worm motility, male–female pairing, and oviposition after 144 h, with an IC_50_ value of 32.09 μg/mL. However, the macromolecular target of these compounds, was not determined in this study. In 2015, Fonseca et al.^2^ synthesized and tested 29 thiosemicarbazones with a direct action on cathepsin B1 from *Schistosoma mansoni* (*Sm*CB1), revealing a set of five compounds with IC_50_ values under 10 μM. However, all the other analogs, showed only moderate inhibitory activity (Fig. 1B). Cathepsin B1 from *S. mansoni* is a peptidase responsible for hemoglobin degradation in the worm’s gut and, is the main protease involved in nutrition. This protease is crucial not only for worm survival but also, in the case of females, for oviposition, which ensures the continuity of the biological cycle^3^.

As *Sm*CB1 enzyme is considered a validated target, it warrants further efforts in the search for new inhibitors. Upon examining the structures presented by Fonseca et al. structures, it becomes evident that the majority of them also feature nitrogen in either the ortho or para position of the aromatic ring, specifically in relation to the azomethine group within their structures. While it appears that the activity of these compounds is not solely dependent on the presence of this nitrogen atom, it is noteworthy that most of the highly active compounds do indeed contain it. Taken this observation into account an in pursuit of rational molecular modifications to these structures, we proposed incorporating 4-amino-7-chloroquinoline as the aromatic system attached to hydrazones analogs of the compounds studied by Fonseca et al.^2^.

The rationale behind this proposal is based on the knowledge that chloroquine, a well-known widely used drug against the protozoa *Plasmodium* spp., features an aromatic nitrogen in the same position. It is also well-established that chloroquine interacts with the heme group from hemoglobin^4^. Given that the primary source of nutrients for *S. mansoni* is the hemoglobin, and *Sm*CB1 is responsible for its degradation, hybrid compounds combining the characteristics of both compound classes could potentially achieve a synergistic effect within the parasite’s digestive system.

Here, we present the results of synthesizing these compounds and conducting in vitro and in vivo biological screening against adult male and female *S. mansoni* worms.

## Results and discussions

**Figure 2:**
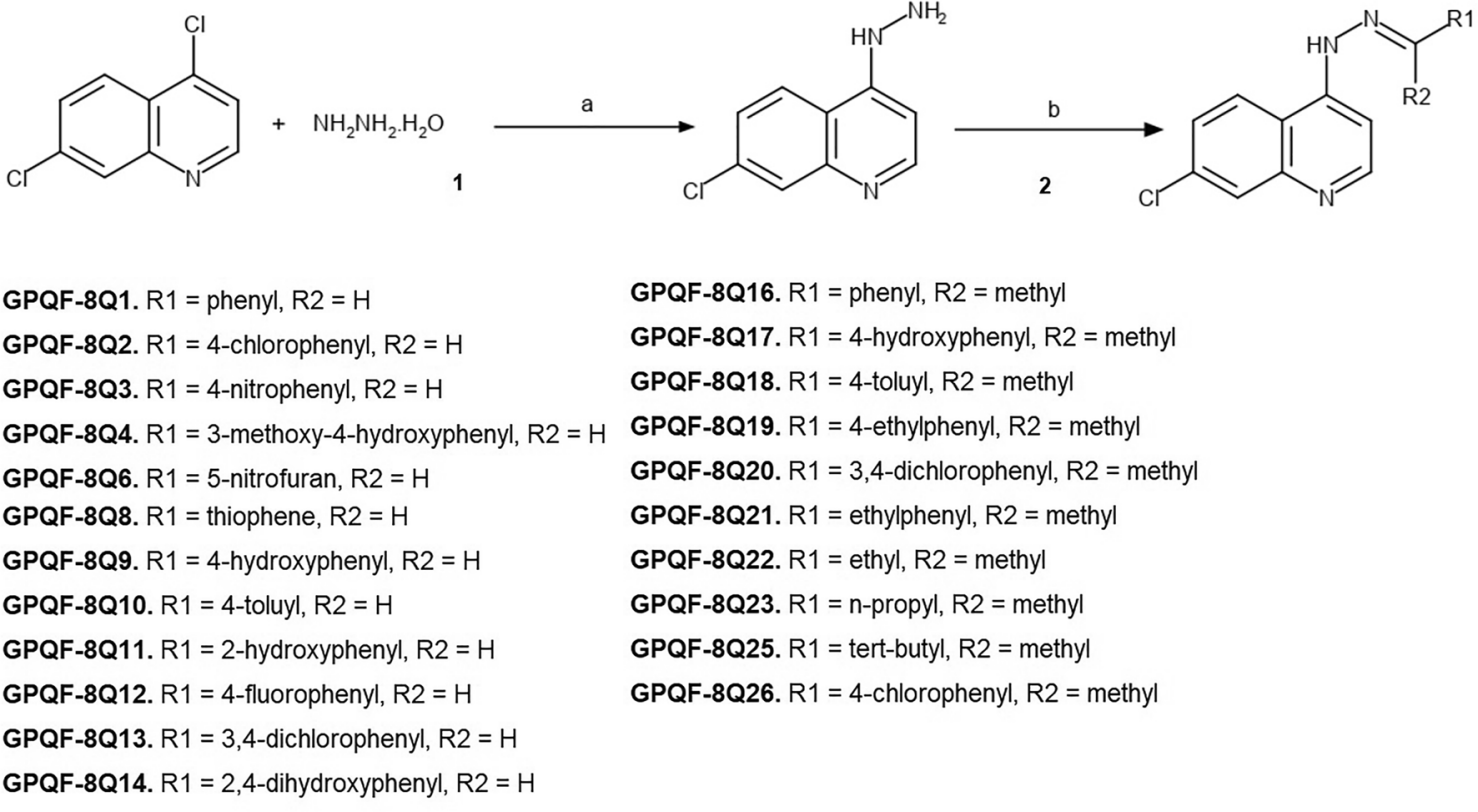
Synthetic approach to obtain the 22 analogs of 7‐chloro‐4‐[2‐(phenylmethylidene)hydrazin‐1‐yl]quinoline. General synthesis of 7-chloro-4-hydrazinylquinoline (1) and 7-chloro-4-hydrazinylquinoline derivatives (2). Reagents and conditions: (**a**) ethanol, reflux 80 °C, for 15 h; (**b**) corresponding aldehydes and ketones, ethanol/HOAc 25% or MeOH/HOAc 25%, reflux 80 °C, 3-24 h. In specific cases (**GPQF-8Q1**, **8Q22**, **8Q23**, and **8Q25**) the reaction occurred at room temperature for 24 h.

A series of 22 analogs of 7‐chloro‐4‐[2‐(phenylmethylidene)hydrazin‐1‐yl]quinoline were synthesized through the coupling of 7‐chloro‐4‐hydrazinylquinoline with various aldehydes or ketones, as described in Methods Session and illustrated in Fig. 2. In general, the compounds were obtained in solid form with good purity, which was confirmed through ^1^H-NMR and melting point analysis, and in moderate to good yields. As expected, Aldehyde derivatives, were obtained in higher yields than the ketone derivatives.

Table 1 lists the structures of the compounds in the series, along with their yields and the results of the initial in vitro trial conducted at 50 micromolars.

**Table 1:** Set of analogs synthesized and screened against *S. mansoni* adult worms.

ID			Synthetic yield (%)	CLogP	Activity against 49-day-old *S. mansoni* worms at 50 µM
	R1	R2			
GPQF-8Q1	Phenyl	H	52	4.56	Inactive
**GPQF-8Q2**	**4-Chlorophenyl**	**H**	**75**	**5.17**	**Active**
GPQF-8Q3	4-Nitrophenyl	H	59	4.50	Inactive
GPQF-8Q4	3-Methoxy-4-hydroxyphenyl	H	63	4.10	Inactive
GPQF-8Q6	5-Nitrofuran	H	58	3.65	Inactive
**GPQF-8Q8**	**Thiophene**	**H**	**61**	**4.48**	**Active**
**GPQF-8Q9**	**4-Hydroxyphenyl**	**H**	**77**	**4.26**	**Active**
**GPQF-8Q10**	**4-toluyl**	**H**	**96**	**5.06**	**Active**
**GPQF-8Q11**	**2-Hydroxyphenyl**	**H**	**96**	**4.26**	**Active**
GPQF-8Q12	4-Fluorophenyl	H	86	4.71	Inactive
GPQF-8Q13	3,4-Dichlorophenyl	H	43	5.77	Inactive
GPQF-8Q14	2,4-Dihydroxyphenyl	H	55	3.96	Inactive
GPQF-8Q16	phenyl	Methyl	52	4.41	Inactive
GPQF-8Q17	4-Hydroxyphenyl	Methyl	54	4.10	Inactive
GPQF-8Q18	4-Toluyl	Methyl	41	4.92	Inactive
GPQF-8Q19	4-Ethylphenyl	Methyl	49	5.37	Inactive
GPQF-8Q20	3,4-Dichlorophenyl	Methyl	45	5.62	Inactive
GPQF-8Q21	Ethylphenyl	Methyl	49	4.82	Inactive
GPQF-8Q22	Ethyl	Methyl	77	3.68	Inactive
GPQF-8Q23	*n*-Propyl	Methyl	51	4.13	Inactive
GPQF-8Q25	*Tert*-butyl	Methyl	59	4.78	Inactive
GPQF-8Q26	4-Chlorophenyl	Methyl	67	5.01	Inactive

Significant values in bold.

The ease of synthesis, along with good yields, the use of relatively inexpensive reagents, and the production of solid compounds, all represent desirable characteristics for drug candidates targeting neglected diseases like schistosomiasis.

All compounds were subjected to an initial trial at 50 µM. In the context of testing compounds against *S. mansoni*, only chemical compounds or natural extracts that demonstrated any activity at this concentration were considered significant for further in vitro and in vivo testing. Accordingly, five out of the 22 final compounds met these criteria and were selected to follow with the experimental investigations.

This first trial also uncovered some intriguing insights into the relationships between the chemical properties of the compounds and their biological behavior. Notably, all ketone derivatives proved to be inactive against the adult worms, at least within the established concentrations limits of the test. Even compounds with similar overall structures but derived from equivalent aldehydes or ketones, such as the pairs **GPQF-8Q9**/**GPQF-8Q17**, and **GPQF-8Q10**/**GPQF-8Q18,** exhibited contrasting biological responses at 50 µM, with only the aldehydes derivatives displaying activity. This strongly suggests the presence of a macromolecular target with specific stereochemical and/or volume requirements. The ketone derivatives, which possess a three-dimensional structure distinct from the typically flat structure of the azomethines derived from aldehydes, were inactive. These initial results point towards a stereo-specific binding site, where only the flat derivatives could exert significant inhibitory activity.

**Figure 3:**
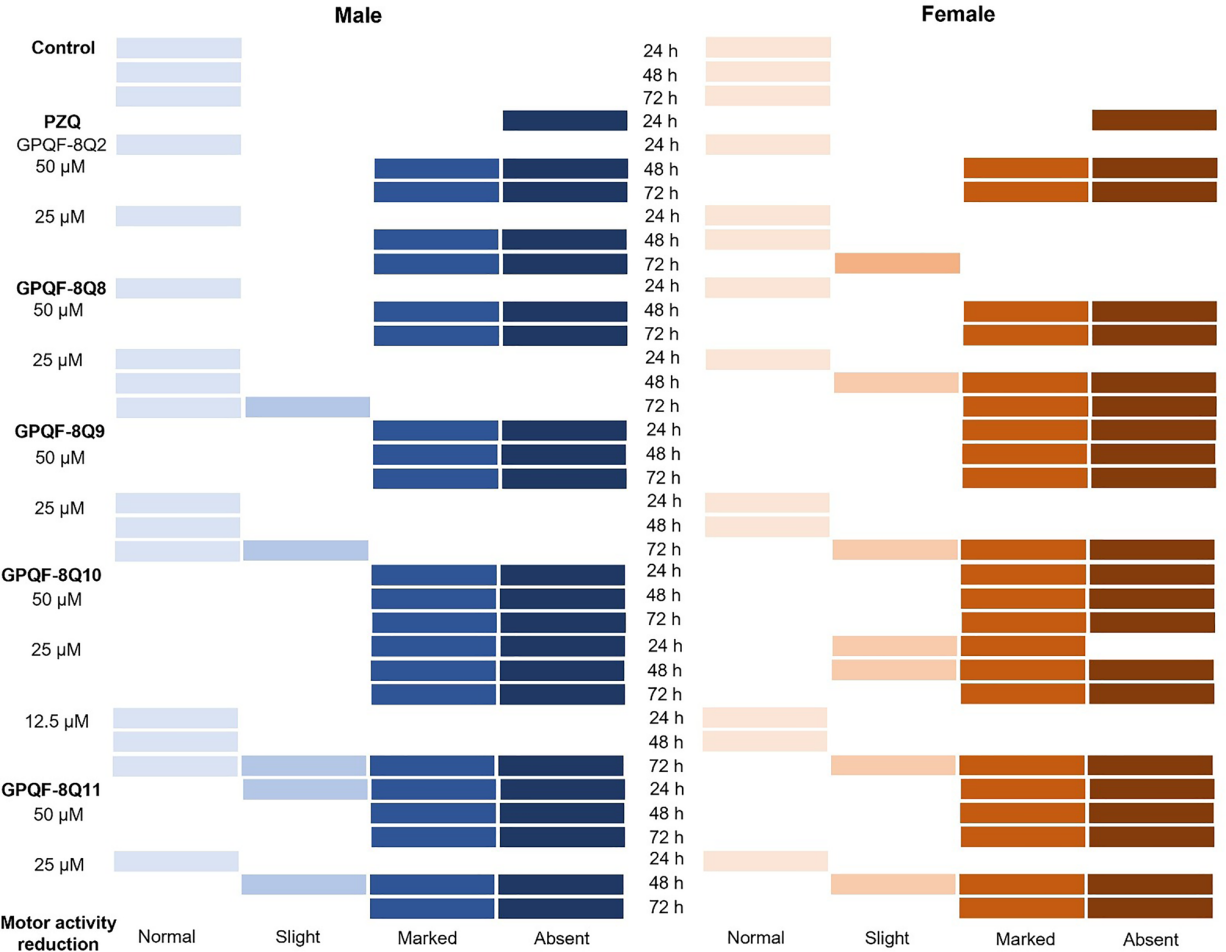
The viability of adult *S. mansoni* following incubation with five active analogs.

The five active analogs were subsequently subjected to in vitro assays at lower concentrations. The results, which include reductions in worm motor activity categorized as normal, slight, significant, or absent and are separated by sex, are presented in Fig. 3.

**Figure 4:**
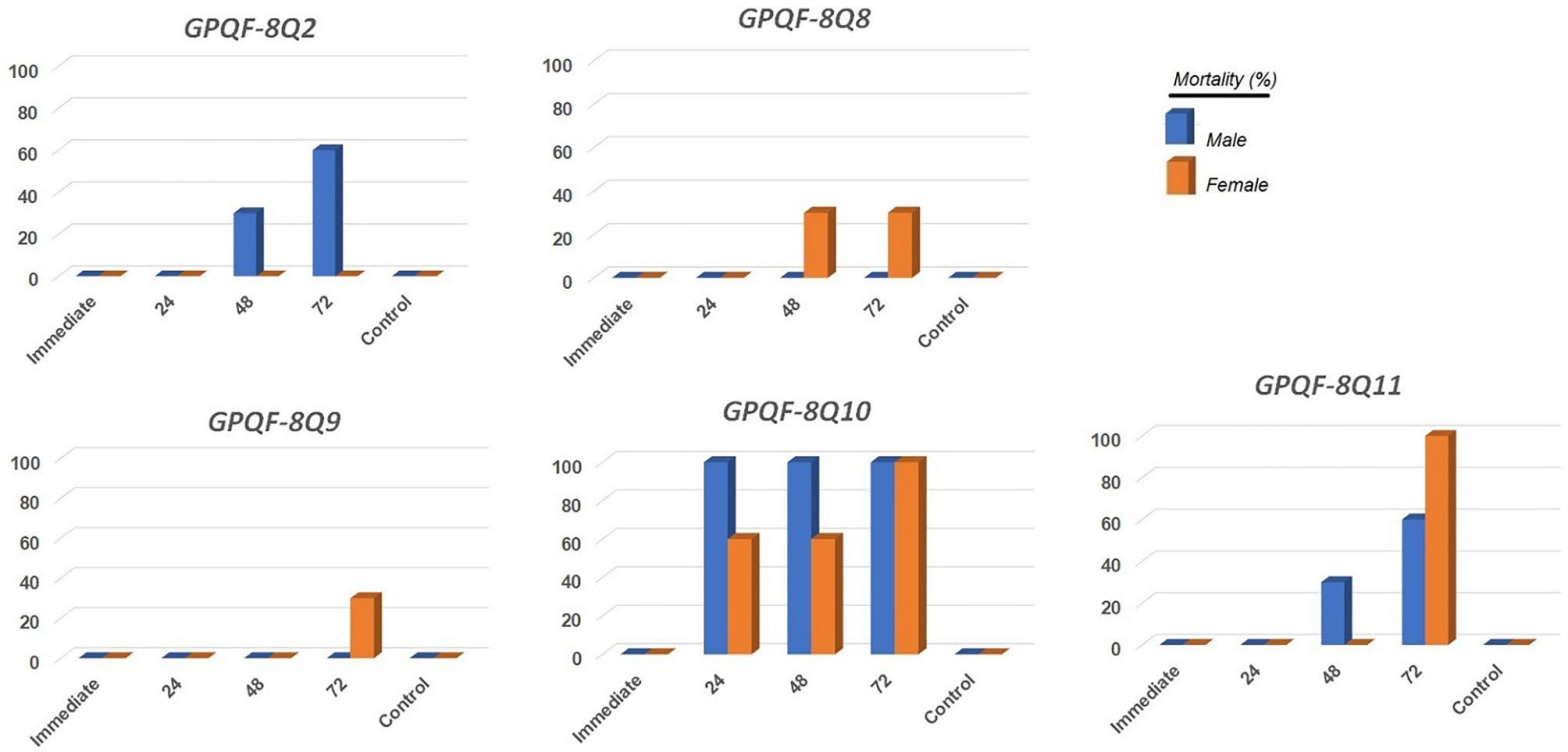
In vitro antischistosomal activity results after exposure to the compounds at 25 µM. Control = DMSO.

All compounds exhibited significant interference with worm motility at concentrations of 50 µM or lower. Distinctions in their biological behavior became apparent at 25 µM, where some compounds demonstrated effective interference with motility and lethality against the worms (Fig. 4).

With regard to lethality, **GPQF-8Q10** proved to be the most effective analog in vitro, killing the worms within the first 24 h at 25 µM. A noticeable reduction in motility followed a similar pattern. It appears that there may be differences in sensitivity between males and females, although these results do not provide a detailed understanding of how each gender responds to the treatment. Notably, females appeared to be affected by all the active analogs to varying degrees and at different time intervals, with the exception of **GPQF-8Q2**.

Another noteworthy observation is the compounds require some time to exert their lethal effects, as none of them killed the worms immediately. This time-dependent activity aligns with the design concept of these compounds, as their potential inhibition of *Sm*CB1 suggests that they induced death by interfering with nutrition, a process that naturally takes some time to occur.

The active compounds were subjected to testing against Vero cells (African green monkey kidney cells), a commonly employed cell line in toxicity studies, especially in the realm of antischistosomal drug discovery^13,17^. The cytotoxicity concentration that led to a 50% cell death rate (CC_50_) was determined. The results, including the effective concentration that caused 50% inhibition (EC_50_) and the selectivity index, are summarized in Table 2.

**Table 2:** Effective concentration 50% (EC_50_) against *S. mansoni* adult worms and cytotoxic concentration 50% (CC_50_) of analogs of the 7‐chloro‐4‐[2‐(phenylmethylidene)hydrazin‐1‐yl]quinoline after 72 h.

Compounds	*S. mansoni *EC_50_ (μM)		Mammalian cells CC_50_ (μM)	SI
	Male	Female		
**GPQF-8Q2**	19.6 [14.3–22.5]	34.7 [2.2–3.4]	> 200	> 5.8
**GPQF-8Q8**	35.8 [0.7–1.1]	28.5 [0.7–1.1]	> 200	> 5.6
**GPQF-8Q9**	36.1 [0.7–1.1]	30.2 [0.7–1.1]	> 200	> 5.5
**GPQF-8Q10**	13.3 [0.7–1.1]	15.6 [0.7–1.1]	> 200	> 12.8
**GPQF-8Q11**	17.1 [0.7–1.1]	15.8 [0.7–1.1]	> 200	> 11.7
**PZQ**	0.7 [0.6–0.9]	1.0 [0.8–1.1]	> 200	> 200

Cytotoxicity activity was assessed using an MTT assay. The SI values were calculated by dividing CC_50_ values obtained on Vero cells with EC_50_ values determined on schistosomes (based on the highest concentration). Values are calculated from three experiments, and each experiment was performed with three replicates. The 95% confidence interval is in square brackets.

*EC*_*50*_ effective concentration 50% based on mortality of *S. mansoni* adult worms, *CC*_*50*_ cytotoxic concentration 50% against Vero cells after 48 h incubation, *SI* selectivity index, *PZQ* praziquantel.

The measured EC_50_ values confirm the initial in vitro observations. The Vero cells assay indicated low toxicity of these compounds against these cells and the calculated selectivity indexes suggests that good selectivity could be achieved.

**Figure 5:**
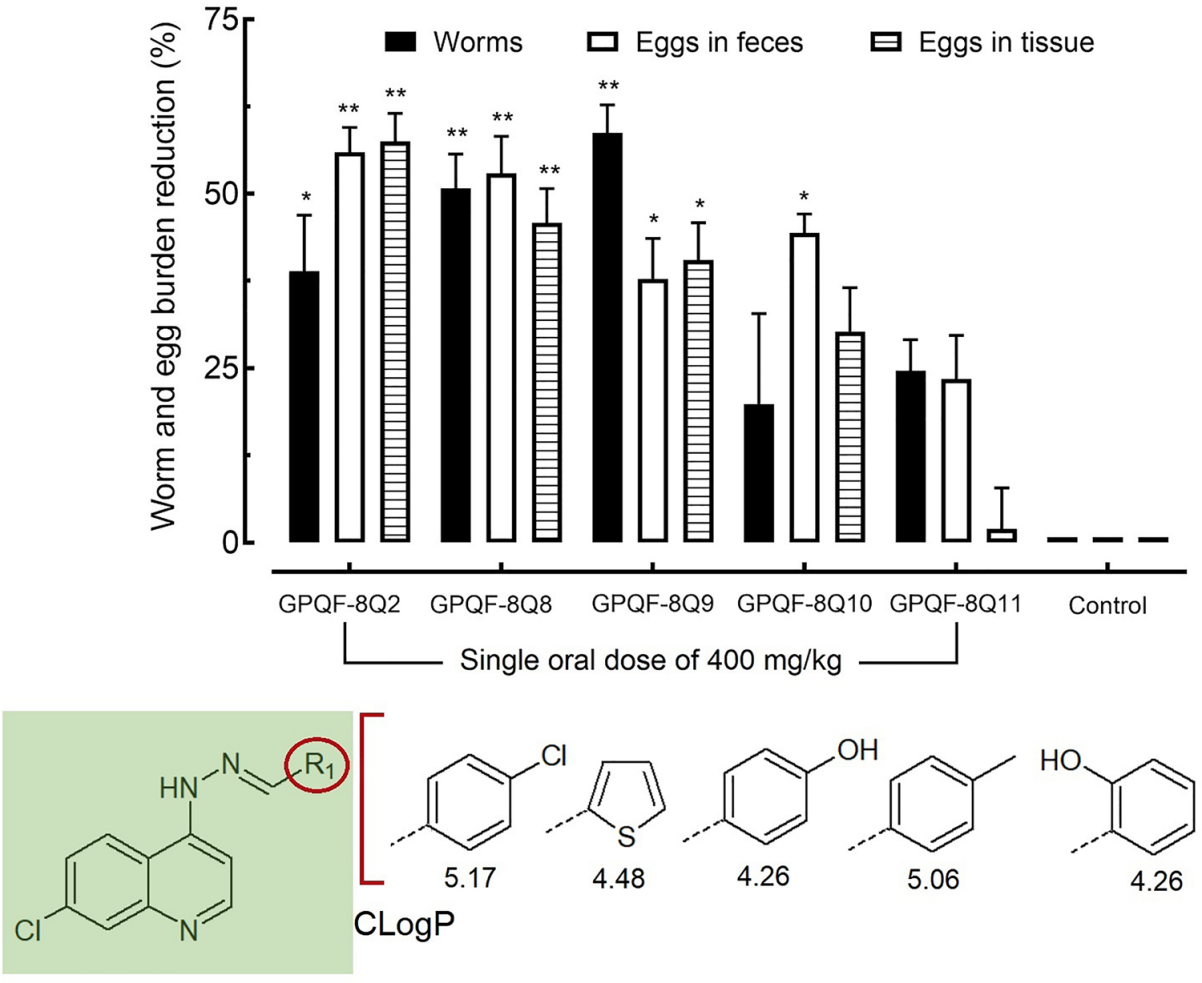
Biological in vivo results after 400 mg/kg single dose of the compounds. Efficacy of analogs of the 7‐chloro‐4‐[2‐(phenylmethylidene)hydrazin‐1‐yl]quinoline in a schistosomiasis animal model. Drugs were administered orally using a single dose of 400 mg/kg to *S. mansoni*-infected mice. On day 56 post-infection, all animals were humanely euthanized. Parasite burdens were determined by sex (male and female parasites). Egg burdens were determined by counting eggs in the feces (Kato-Katz technique) and in the tissue (oogram analysis in the intestine). Data are presented as the mean ± SD (*n* = 5 per group). The numbers represent the percentages of worms or egg reduction vs. infected untreated control. The partition coefficient (CLogP) is indicated just below the chemical structures. *P < 0.05; **P < 0.01 compared to the control group infected and treated with the vehicle.

The next step in this investigation was the in vivo tests (Fig. 5).

Remarkably, the best-performing compound in in vitro tests, **GPQF-8Q10**, did not exhibit the same level of effectiveness in vivo*.* It might be tempting to associate this lack of in vivo activity with its high partition coefficient (CLogP 5.06). However, this does not appear to be the case, as the analog **GPQF-8Q2**, which had the highest CLogP value (5.17), showed the best reduction in oviposition. The most significant reductions in worm burden were achieved by compounds **GPQF-8Q8** and **GPQF-8Q9**, with reductions of 50.8 and 58.7%, respectively. **GPQF-8Q8** displayed the most comprehensive in vivo biological activity, as it was also the most effective compound in interfering with egg production. It led to reductions in both eggs found in feces (52.8%) and immature eggs found in the intestines (45.8%). The presence of the thiophene ring in this compound also contributed positively to lipophilicity, which could suggest that this factor plays a role in the action on oviposition.

Even the more “hydrophilic” compounds, **GPQF-8Q9** and **GPQF-8Q11** (both having a CLogP of 4.26 as they are position isomers), exhibited intriguing in vivo biological behaviors. Interestingly, only **GPQF-8Q9** showed significant in vivo activity, suggesting the presence of an important steric factor related to theses structures. Ortho substituents are well-known to influence the conformation of rings, forcing them out of the plane of the molecule, in this case, the plane of the 7-chloroquinoline ring. This could explain why **GPQF-8Q11** did not exhibit any in vivo activity at all. Since the only difference between these two compounds is the steric positioning of hydroxy group, the significantly different biological responses to both compounds strongly indicate the presence of an endogenous receptor with selective steric specificities.

Another noteworthy observation is that the two best-performing in vivo, **GPQF-8Q8** and **GPQF-8Q9**, were also the only two compounds that predominantly exhibited in vitro activity against females. In a study by Liu et al.^5^ in 2014, it was demonstrated that *S. japonicum* expresses a network of proteases in its gut, with Cathepsin B1 being the predominant enzyme. Importantly, they observed that this enzyme was upregulated in female gut. Therefore, the preference of **GPQF-8Q8** and **GPQF-8Q9** for acting on females could be a strong indication that *Sm*CB1 is indeed the target of these compounds.

It is important to highlight, however, that the *Sm*CB1 could not be the only possible macromolecular target since the in vitro tests do not guarantee the specificity for only one target. Only enzymatic inhibitory assays would prove that this enzyme would be a real target for these compounds, and they have already been performed by our group. But even in this case the selectivity for just one target could not be assured.

*Caenorhabditis elegans* is a nematode commonly employed as an in vivo toxicity model ^6,7^. Its utility extends to research aiming to discover new anthelmintic agents ^8,9^. In order to delve deeper into the potential toxicity of analogs of 7‐chloro‐4‐[2‐(phenylmethylidene)hydrazin‐1‐yl]quinoline, we assessed the survival of *C. elegans* exposed to various compound concentrations. As shown in Table 3, none of the compounds exhibited any toxic effects, and all nematodes displayed a sinusoidal shape and mobility comparable to the untreated animals.

**Table 3:** Toxicity of analogs of the 7‐chloro‐4‐[2‐(phenylmethylidene)hydrazin‐1‐yl]quinoline on *Caenorhabditis elegans* model.

Concentrations (μM)	GPQF-8Q2	GPQF-8Q8	GPQF-8Q9	GPQF-8Q10	GPQF-8Q11	LVM
0^b^	100^a^	100^a^	100^a^	100^a^	100^a^	100^a^
10	100	100	100	100	100	0
25	100	100	100	100	100	N.D
50	100	100	100	100	100	N.D
100	100	100	100	100	100	N.D
200	100	100	100	100	100	N.D

*N.D.* not determined.

^a^Survival percentage of *C. elegans* nematode receiving no treatment ^b^(control) or exposed to analogs of the 7‐chloro‐4‐[2‐(phenylmethylidene)hydrazin‐1‐yl]quinoline at different concentrations. Except with levamisole (LVM), which was used as a positive control, the survival of the treated nematode is similar to that of the untreated controls.

## Conclusion

Considering the results reported herein, the 7‐chloro‐4‐[2‐(phenylmethylidene)hydrazin‐1‐yl]quinoline scaffold appears to be a promising starting point for the development of new antischistosomal drugs. Among the 22 tested compounds, five demonstrated schistosomicidal activity in the in vitro assay, with **GPQF-8Q10** emerging as the most potent compound at 25 µM, leading to the death of all worms within 72 h. Notably, this compound displayed values of 13.3 and 15.6 µM against male and female worms, respectively.

In the in vivo assay, **GPQF-8Q8** displayed the most comprehensive biological activity, reducing both the number of eggs found in feces (52.8%) and immature eggs found in the intestines (45.8%). The compounds also demonstrated low cytotoxicity against Vero cells and a favorable selectivity index. Furthermore, none of the compounds exhibited any toxic effect in the in vivo toxicity assay using the *C. elegans* model.

Although further studies are needed to determine the target, the collective findings strongly suggest the potential of the hydrazone quinoline scaffold in the quest for new antischistosomal leads.

## Methods

### Chemistry

Reagents and solvents were obtained from commercial suppliers without the need for further purification. Chemical reactions were monitored by thin layer chromatography (TLC) using Merck 60 F_254_ Silica gel on TLC aluminum foils, hexane/ethyl acetated 1:2 was employed as eluent and the plates were visualized by ultraviolet (UV) irradiation (254 nm). Melting point analyses were conducted in triplicate using a Marte científica PDF III apparatus. Infrared (IR) analyses were carried out using an IR Affinity-1 Shimadzu Fourier Transform Infrared Spectrophotometer. Samples were prepared with anhydrous KBr, and the analyses covered the wave number region between 4000 and 400 cm^−1^. ^1^H-NMR analyses were performed on a Bruker-300 Ultrashield operating at 300 MHz, with solvents DMSO-d_6_ or CDCl_3_. The chemical shift values (δ) were expressed in parts per million (ppm), employing tetramethylsilane (TMS) as the internal reference. The following abbreviations were used to describe the multiplicities: s = singlet; d = doublet; dd = double doublet; t = triplet; m = multiplet. Coupling constants (*J*) were described in Hertz.

All the structural data collected are provided in the Supplementary Information (Fig. S1–S46).

The synthesis of the intermediate 7-chloro-4-hydrazinylquinoline was conducted following a previously described methodology in the literature^10–12^. A total of 5 mmol of 4,7-Dichloroquinoline was dissolved in 10 mL of ethanol and placed in a round-bottom flask equipped with a reflux system. After 5 min of stirring, 25 mmol of hydrazine hydrate 80% (w/v) was added to the reaction. The solution was then heated under reflux for 15 h at 80–90 °C. After this period, the solution was refrigerated for approximately 8 h. The resulting dark yellow precipitate was washed with cold distilled water and filtered under reduced pressure. The crude compound was subsequently recrystallized from ethanol and a yellow crystalline solid was obtained.

#### 7-Chloro-4-hydrazinylquinoline

Prepared in accordance with the synthetic methodology previously described. Yellow crystalline solid. **Yield** 80%. **M.P**. 220–224 °C (lit.^11^ 225–226°C). ^**1**^**H-NMR**-(DMSO-d_6_)—*δ* = ppm: 4.45 (sl, 2H, H_12_); 6.86 (d, 1H, *J* = 6 Hz, H_3_); 7.38 (dd, 1H, *J*_1_ = 9Hz, *J*_2_ = 3 Hz, H_7_); 7.75 (s, 1H, H_9_); 8.16 (d, 1H, *J* = 9 Hz, H_6_); 8.40 (d, 1H, *J* = 9 Hz, H_2_) 8.59 (sl, 1H, H_11_);. **IR**—νNH_2_ and νNH 3282 and 3253 cm^-1^; νCH 3053 cm^−1^; νC = C 1612 cm^−1^; νC = N 1570 cm^−1^.

#### General procedure: quinoline derivatives

The quinoline derivatives were obtained following the synthetic methodology reported in the literature^12–14^. First, (1 mmol) of corresponding aldehyde or ketone was introduced into a round-bottom flask, and in most cases*, approximately 8 mL of a ethanol/acetic acid 25% solution was added to the round-bottom flask. The solution was kept under agitation until the complete solubilization of the corresponding reagents. Then, (1 mmol) of 7-chloro-4-hydrazinylquinoline was added, and the reaction mixture was heated under reflux** (about 80 °C) for about 3–17 h. After the reflux complete, the resulting mixture was concentrated under reduced pressure, and the crude compounds were washed with cold water and cold ethyl ether, then filtered under reduced pressure to obtain the desired products.

*In some cases, the solvent employed was ethanol **(GPQF-8Q1**) or a solution of MeOH/HAOc 25% (**GPQF-8Q6**).

**In the case of **GPQF-8Q1**, **8Q22**, **8Q24**, and **8Q25**, the reaction was kept at room temperature for 24 h.

#### 4-[(2*E*)-2-benzylidenehydrazinyl]-7-chloroquinoline (GPQF-8Q1)

Prepared following the general synthetic procedure of quinoline derivatives, which involved using 7-chloro-4-hydrazinylquinoline (1mmol) and benzaldehyde (1 mmol) as reactants, ethanol was employed as the solvent, and the reaction was maintained at room temperature for 24 h. Yellow solid. **Yield** 52%. **M.P**. 220–224 °C (lit.^13^ 223–225°C). ^**1**^**H-NMR-(DMS**O-*d*_6_)—*δ* = ppm: 7.37 (d, 1H, *J* = 6 Hz, H_3_); 7.42–7.51 (m, 3H, H_16_, H_17_ H_18_); 7.57 (dd, 1H, *J*_1_ = 9Hz, *J*_2_ = 3, H_7_); 7.81 (d, 2H, *J* = 6 Hz; H_15_ e H_19_); 7.86 (s, 1H, H_9_); 8.42 (d, 1H, *J* = 9 Hz, H_6_) 8.44 (s, 1H, H_13_); 8.47 (d, 1H, *J* = 6 Hz, H_2_) . **IR—**νNH 3446 cm^−1^; νCH 3188 and 2891 cm^−1^; νC = C 1618 cm^−1^; νC = N 1577 cm^−1^.

#### 7-Chloro-4-[(2*E*)-2-(4-chlorobenzylidene)hydrazinyl]quinoline (GPQF-8Q2)

Prepared in accordance with the general synthetic procedure for quinoline derivatives, which involved using 7-chloro-4-hydrazinylquinoline (1mmol) and 4-chlorobenzaldehyde (1 mmol), a solution of ethanol and acetic acid 25% was employed as the solvent. The reaction was conducted under reflux at 80 °C during 3 h. Orange solid. **Yield** 75%. **M.P**. 320–324 °C. ^**1**^**H-NMR**-(DMSO-*d*_6_)—*δ* = ppm: 7.60 (d, 2H, *J* = 9, H_16_, H_18_), 7.65 (d, 1H, *J* = 6, H_3_), 7.88 (d, 1H, *J* = 9 Hz, H_7_), 7.94 (d, 2H, *J* = 9 Hz, H_15_, H_19_), 8.07 (d, 1H, *J* = , H_9_), 8.69 (d, 1H, *J* = 6 Hz, H_6_), 8.75–8.78 (m, 2H, H_13_, H_2_). **IR**—νNH 3446 cm^−1^; νCH 2875 and 2698 cm^−1^; νC = C 1627 cm^−1^; νC = N 1612 cm^−1^.

#### 7-Chloro-4-[(2*E*)-2-(4-nitrobenzylidene)hydrazinyl]quinoline (GPQF-8Q3)

7-Chloro-4-hydrazinylquinoline (1mmol) and 4-nitrobenzaldehyde (1 mmol) were coupled in the presence of ethanol and acetic acid 25% solution. The reaction mixture was refluxed at 80 °C for 18 h, following the general synthetic procedure. Pale-yellow solid. **Yield** 59%. **M.P**. 348–350 °C. ^**1**^**H-NMR**-(DMSO-*d*_6_)—*δ* = ppm: 7.74 (d, 1H, *J* = 6Hz, H_3_), 7.93 (dd, 1H, *J*_1_ = 9Hz, *J*_2_ = 3Hz, H_7_), 8.08 (s, 1H, H_9_), 8.19 (d, 2H, *J* = 9Hz, H_15_, H_19_), 8.36 (d, 2H, *J* = 9Hz, H_16_, H_18_), 8.73–8.81 (m, 2H, H_2_, H_6_, H_13_). **IR**—νNH 3446 cm^−1^; νCH 2646 cm^−1^; νC = C 1614 cm^−1^.

#### 4-{(*E*)-[2-(7-chloroquinolin-4-yl)hydrazinylidene]methyl}-2-methoxyphenol (GPQF-8Q4)

7-Chloro-4-hydrazinylquinoline (1 mmol) and 4-hydroxy-3-methoxybenzaldehyde (1 mmol) were coupled following the general synthetic procedure, in the presence of ethanol/acetic acid 25% solution and the reaction was maintained under reflux at 80 °C for 3 h. Yellow solid. **Yield** 63%. **M.P**. 284–288 °C (lit.^10^ 272–275 °C). ^**1**^**H-NMR**-(DMSO-*d*_6_)—*δ* = ppm: 3.89 (s, 3H, H_22_); 6.94 (d, 1H, *J* = 6 Hz, H_3_); 7.25 (d, 1H, *J* = 3Hz, H_15_); 7.45 (s, 1H, H_19_); 7.61 (d, 1H, *J* = 9 Hz, H_16_); 7.84 (d, 1H, *J* = 9 Hz, H_7_); 8.10 (s, 1H, H_9_); 8.61 (d, 1H, *J* = 6 Hz, H_6_); 8.77 (s, 1H, H_13_); 8.91 (d, 1H, *J* = 9 Hz, H_2_); 9.96 (sl, 1H, H_11_); 12.98 (sl, 1H, H_23_). **IR**—νOH and νNH 3421 cm^−1^; νCH 3209 cm^−1^; νC = C 1608 cm^−1^; νC = N 1589 cm^−1^.

#### 7-Chloro-4-{(2*E*)-2-[(5-nitrofuran-2-yl)methylidene]hydrazinyl}quinoline (GPQF-8Q6)

Prepared following the general synthetic procedure for quinoline derivatives, which involved using 7-chloro-4-hydrazinylquinoline (1mmol) and 5-nitrofuran-2-carbaldehyde (1 mmol), a solution of methanol and acetic acid 25% was used as the solvent. The reaction was maintained under reflux at 80 °C for 17 h. Mustard yellowish solid. **Yield** 58%. **M.P**. 284–286 °C (lit.^15^ 238–240 °C). ^**1**^**H-NMR**-(DMSO-*d*_6_)—*δ* = ppm: 7.46 (d, 1H, *J* = 3Hz, H_4_), 7.52 (d, 1H, *J* = 6Hz, H_12_), 7.83 (d, 1H, *J* = 3 Hz, H_4_), 7.86 (d, 1H, *J* = 6Hz, H_18_), 8.07 (s, 1H, H_16_), 8.71 (d, 1H, *J* = 6 Hz, H_19_), 8.76 (s, 1H, H8) 8.82 (d, 1H, *J* = 9Hz, H_13_). **IR**—νNH 3446 cm^−1^; νCH 3082 and 2646 cm^−1^; νC = C 1614 cm^−1^; νC = N 1589 cm^−1^.

#### 7-Chloro-4-[(2*E*)-2-(thiophen-2-ylmethylidene)hydrazinyl]quinoline (GPQF-8Q8)

7-Chloro-4-hydrazinylquinoline (1mmol) and thiophene-2-carbaldehyde (1 mmol) were coupled following the general synthetic procedure, in the presence of a solution of ethanol and acetic acid 25%. The reaction mixture was maintained under reflux 80 °C for 16 h. Yellowish bright solid. **Yield** 61%. **M.P**. 224–226 °C (lit.^15^ 231–232 °C). ^**1**^**H-NMR**-(DMSO-*d*_6_)—*δ* = ppm: 7.15–7.19 (m, 2H, H_3_, H_10_), 7.49 (d, 1H, *J* = 3Hz, H_4_), 7,57 (dd, 1H, *J*_1_ = 9Hz, *J*_2_ = 3Hz, H_16_), 7.66 (d, 1H, *J* = 6 Hz, H_2_), 7.82 (s, 1H, H_14_), 8.37–8.43 (m, 2H, H_11_, H_17_), 8.66 (s, 1H, H_6_). **IR**—νNH 3444 cm^−1^; νCH 3074cm^−1^; νC = C 1610 cm^−1^; νC = N 1550 cm^−1^.

#### 4-{(*E*)-[2-(7-chloroquinolin-4-yl)hydrazinylidene]methyl}phenol (GPQF-8Q9)

7-Chloro-4-hydrazinylquinoline (1mmol) and 4-hydroxybenzaldehyde (1 mmol) were coupled following the general synthetic procedure, in the presence of a solution of ethanol and acetic acid 25% and maintained under reflux at 80 °C for 16 h. Orange solid. **Yield** 77%. **M.P**. 280–284 °C (lit.^10^ 233–235 °C). ^**1**^**H-NMR**-(DMSO-*d*_6_)—*δ* = ppm: 6.85 (d, 2H, *J* = 9Hz, H_16_, H_18_), 7.26 (d, 1H, *J* = 3 Hz, H_3_), 7.51 (d, 1H, *J* = 6 Hz, H_7_), 7.62 (d, 2H, *J* = 9 Hz, H_15_, H_19_), 7.82 (sl, 1H, H_9_), 8.31 (s, 1H, H_13_), 8.35 (d, 1H, *J* = 12Hz, H_6_); 8.47 (d, 1H, *J* = 12 Hz, H_2_) 9.97 (sl, 1H, H_21_), 11,03 (sl, 1H, H_11_). ). **IR**—νO-H and νNH 3425 cm^−1^; νCH 3030cm^−1^; νC = C 1608 cm^−1^; νC = N 1552 cm^−1^.

#### 7-Chloro-4-[(2*E*)-2-(4-methylbenzylidene)hydrazinyl]quinoline (GPQF-8Q10)

Prepared following the general synthetic procedure, which involved using 7-chloro-4-hydrazinylquinoline (1mmol) and 4-methylbenzaldehyde (1 mmol), in the presence of a solution of ethanol and acetic acid 25%. The reaction was maintained under reflux at 80 °C for 7 h. Sharp range crystals. **Yield** 96%. **M.P**. 190–192 °C (lit.^12^ 240–244 °C). ^**1**^**H-NMR**-(DMSO-*d*_6_)—*δ* = ppm: 2.36 (s, 3H, H_21_), 7.29 (d, 2H, *J* = 9Hz, H_16_, H_18_), 7.34 (d, 1H, *J* = 6Hz, H_3_), 7.56 (dd, 1H, *J*_1_ = 9Hz, *J*_2_ = 3Hz, H_7_), 7.69 (d, 2H, *J* = 9Hz, H_15_, H_19_), 7.84 (d, 1H,* J* = 3Hz, H_9_), 8.38–8.41 (m, 2H, H_6_, H_13_), 8.45 (d, 1H, *J* = 6Hz, H_2_). **IR**—νNH 3446 cm^−1^; νCH 3022cm^−1^; νC = C 1618 cm^−1^; νC = N 1579 cm^−1^.

#### 2-{(*E*)-[2-(7-chloroquinolin-4-yl)hydrazinylidene]methyl}phenol (GPQF-8Q11)

Prepared in accordance with general synthetic procedure. 7-chloro-4-hydrazinylquinoline (1mmol) and 2-hydroxylbenzaldehyde (1 mmol) were coupled using a solution of ethanol and acetic acid 25%. The reaction was conducted under reflux at 80 °C for 17 h. Golden yellow solid. **Yield** 96%. **M.P**. 222–226 °C (lit.^13^ 150–151 °C). ^**1**^**H-NMR**-(DMSO-*d*_6_)—*δ* = ppm: 6.90–6.95 (m, 3H, H_3_, H_16_, H_18_), 7.27 (t, 1H, *J* = 7.5Hz, H_17_), 7.48 (d, 1H, *J* = 9Hz, H_7_), 7.69–7.70 (m, 2H, H_6_, H_19_), 8.17 (s, 1H, H_9_), 8.38 (d, 1H, *J* = 9Hz, H_2_), 8.73 (s, 1H, H_13_). **IR**—νO-H and νNH 3427 cm^−1^; νCH 2553cm^−1^; νC = C 1622 cm^−1^; νC = N 1554 cm^−1^.

#### 7-Chloro-4-[(2*E*)-2-(4-fluorobenzylidene)hydrazinyl]quinoline (GPQF-8Q12)

Prepared in accordance with general synthetic procedure. 7-chloro-4-hydrazinylquinoline (1mmol) and 4-fluorobenzaldehyde (1 mmol) were coupled, using a solution of ethanol and acetic acid 25%. The reaction was conducted under reflux at 80 °C for 17 h. Mustard yellow solid. **Yield** 86%. **M.P**. 236–241 °C (lit.^13^ 225–226 °C). ^**1**^**H-NMR**-(DMSO-*d*_6_)—*δ* = ppm: 7.28–7.35 (m, 3H, H_3_, H_15_, H_19_), 7.56 (dd, 1H, *J*_1_ = 9Hz, *J*_2_ = 3Hz, H_7_), 7.84–7.89 (m, 3H, H_9_, H_16_, H_18_), 8.37–8.42 (m, 3H, H_2_, H_6_, H_13_). **IR**—νNH 3421 cm^−1^; νCH 3209cm^−1^; νC = C 1608 cm^−1^; νC = N 1571 cm^−1^.

#### 7-Chloro-4-[(2*E*)-2-(3,4-dichlorobenzylidene)hydrazinyl]quinoline (GPQF-8Q13)

7-Chloro-4-hydrazinylquinoline (1mmol) and 3,4-dichlorobenzaldehyde (1 mmol) were coupled, using a solution of ethanol and acetic acid 25% employed as the solvent, and kept under reflux at 80 °C during 16 h, following the general synthetic procedure. Light yellow solid. **Yield** 43%. **M.P**. 320 °C (decomposition). ^**1**^**H-NMR**-(DMSO-*d*_6_)—*δ* = ppm: 7.70 (d, 1H, *J* = 9 Hz, H_3_); 7.79 (d, 1H, *J* = 6Hz, H_16_) 7.84–7.92 (m, 2H, H_7_, H_15_), 8.03 (s, 1H, H_19_) 8.19 (s, 1H, H_9_) 8.63–8.69 (m, 3H, H_2_, H_6_, H_13_). **IR**—νNH 3414 cm^−1^; νCH 2879cm^−1^; νC = C 1616 cm^−1^; νC = N 1589 cm^−1^.

#### 4-{(*E*)-[2-(7-chloroquinolin-4-yl)hydrazinylidene]methyl}benzene-1,3-diol (GPQF-8Q14)

7-chloro-4-hydrazinylquinoline (1mmol) and 3,4-dihydroxybenzaldehyde (1 mmol) were coupled using a solution of ethanol and acetic acid 25% as the solvent. The reaction was conducted under reflux at 80 °C for 16 h, following the general synthetic procedure. Yellow solid. **Yield** 55%. **M.P**. 340 °C (decomposition). ^**1**^**H-NMR**-(DMSO-*d*_6_)—*δ* 6.40 (d, 1H, *J* = 9Hz, H_3_), 6.45 (s, 1H, H_16_), 7.46 (d, 1H, *J* = 9 Hz, H_7_), 7.75 (d, 1H, *J* = 9Hz, H_18_), 7.82 (d, 1H, *J* = 9Hz, H_19_), 8.08 (s, 1H, H_9_), 8,57 (d, 1H, *J* = 6Hz, H_6_), 8.81 (d, 1H, *J* = 9Hz, H_2_) 9.01 (s, 1H, H_13_), 10.13 (s, 1H, H_22_), 10.36 (s, 1H, H_21_). **IR**—νO-H and νNH 3427 cm^−1^; νCH 2897 cm^−1^; νC = C 1631 cm^−1^; νC = N 1606 cm^−1^.

#### 7-Chloro-4-[(2*E*)-2-(1-phenylethylidene)hydrazinyl]quinoline (GPQF-8Q16)

Prepared following the general synthetic procedure, which involved using 7-chloro-4-hydrazinylquinoline (1mmol) and 1-phenylethanone (1 mmol) as reactants, and a solution of ethanol and acetic acid 25% as the solvent. The reaction was maintained under reflux at 80 °C for 16 h. Light yellow solid. **Yield** 52%. **M.P**. 302–306 °C. ^**1**^**H-NMR**-(DMSO-*d*_6_)—*δ* = ppm: 2.65 (s, 3H, H_14_), 7.49–7.55 (m, 4H, H_3_, H_18_, H_19_, H_20_), 7.84 (dd, 1H, *J*_1_ = 9Hz, *J*_2_ = 3Hz, H_7_), 8.00 (m, 2H, H_17_, H_21_), 8.18 (s, 1H, H_9_), 8.64 (d, 1H, *J* = 6Hz, H_6_), 8.82 (d, 1H, *J* = 9Hz, H_2_). **IR**—νNH 3408 cm^−1^; νCH 2767cm^−1^; νC = C 1633 cm^−1^; νC = N 1606 cm^−1^.

#### 4-{(1*E*)-1-[2-(7-chloroquinolin-4-yl)hydrazinylidene]ethyl}phenol (GPQF-8Q17)

7-Chloro-4-hydrazinylquinoline (1mmol) and 1-(4-hydroxyphenyl)ethanone (1 mmol) were prepared following the general synthetic procedure, using a solution of ethanol and acetic acid 25% as the solvent. The reaction was conducted under reflux at 80 °C for 16 h. Yellow solid. **Yield** 54%. **M.P**. 320 °C (decomposition). ^**1**^**H-NMR**-(DMSO-*d*_6_)—*δ* = ppm: 2.57 (s, 3H, H_14_), 6.90 (d, 2H, *J* = 9Hz, H_17_, H_21_), 7.45 (d, 1H, *J* = 9Hz, H_3_), 7.81 (dd, 1H, *J*_1_ = 9Hz, *J*_2_ = 3Hz, H_7_), 7.87 (d, 2H, *J* = 9Hz, H_18_, H_20_), 8.13 (s, 1H, H_9_), 8.60 (d, 1H, *J* = 9Hz, H_6_), 8.87 (d, 1H, *J* = 9Hz, H_2_), 10.12 (s, 1H, H_22_). **IR**—νO-H and νNH 3209 cm^−1^; νCH 2787cm^−1^; νC = C 1608 cm^−1^; νC = N 1585 cm^−1^.

#### 7-Chloro-4-{(2*E*)-2-[1-(4-methylphenyl)ethylidene]hydrazinyl}quinoline (GPQF-8Q18)

Prepared in accordance with general synthetic procedure, which involved using 7-chloro-4-hydrazinylquinoline (1mmol) and 1-(4-methylphenyl)ethanone (1 mmol) as reactants, and a solution of ethanol and acetic acid 25% as the solvent. The reaction as maintained under reflux at 80 °C for 16 h. Yellow solid. **Yield** 41%. **M.P**. 310 °C (decomposition). ^**1**^**H-NMR**-(DMSO-*d*_6_)—*δ* = ppm: 2.38 (s, 3H, H_22_), 2.60 (s, 3H, H_14_), 7.32 (d, 2H, *J* = 6Hz, H_18_, H_20_), 7.51 (d, 1H, *J* = 9Hz, H_3_), 7.83 (d, 1H, *J* = 9Hz, H_7_), 7.90 (d, 2H, *J* = 6, H_17_, H_21_), 8.08 (s, 1H, H_9_), 8.82 (d, 1H, *J* = 9Hz, H_2_). **IR**—νNH 3466 cm^−1^; νCH 2555 cm^−1^; νC = C 1610 cm^−1^; νC = N 1585 cm^−1^.

#### 7-Chloro-4-{(2*E*)-2-[1-(4-ethylphenyl)ethylidene]hydrazinyl}quinoline (GPQF-8Q19)

7-Chloro-4-hydrazinylquinoline (1mmol) and 1-(4-ethylphenyl)ethanone (1 mmol) were coupled in accordance with general synthetic procedure. Ethanol and acetic acid 25% were employed as the solvent, and the reaction was maintained under reflux at 80 °C for 16 h. Sharp yellow crystals. **Yield** 49%. **M.P**. 300–304 °C. ^**1**^**H-NMR**-(DMSO-*d*_6_)—*δ* = ppm: 1.22 (t, 3H, *J* = 9Hz, H_23_); 2.62 (s, 3H, H_14_); 2.68 (q, 2H, *J* = 6Hz, H_22_); 7.35 (d, 2H, *J* = 6Hz, H_18_, H_20_); 7,52 (d, 1H, *J* = 6Hz, H_3_); 7,85 (dd, 1H, *J*_1_ = 9Hz, *J*_2_ = 3Hz, H_7_); 7,92 (d, 2H, *J* = 6Hz, H_17_, H_21_); 8,13 (s, 1H, H_9_); 8,65 (d, 1H, *J* = 6Hz, H_6_); 8,86 (d, 1H, *J* = 9Hz, H_2_). **IR**—νNH 3473 e 3415 cm^−1^; νCH 2536 cm^−1^; νC = C 1608 cm^−1^; νC = N 1583 cm^−1^.

#### 7-Chloro-4-{(2*E*)-2-[1-(3,4-dichlorophenyl)ethylidene]hydrazinyl}quinoline (GPQF-8Q20)

Prepared following the general synthetic procedure, which involved using 7-chloro-4-hydrazinylquinoline (1mmol) and 1-(3,4-dichlorophenyl)ethanone (1 mmol) as reactants, and a solution of ethanol/acetic acid 25%. The reaction was conducted under reflux at 80 °C for 16 h. Light orange solid. **Yield** 45%. **M.P**. 294–298 °C. ^**1**^**H-NMR**-(DMSO-*d*_6_)—*δ* = ppm 2.62 (s, 3H, H_14_); 7.57 (s, 1H, H_17_); 7.78 (d,1H, *J* = 9Hz, H_21_); 7.91 (d, 1H, *J* = 12Hz, H_3_); 7.99 (d, 1H, *J* = 6Hz, H_20_); 8.11 (s, 1H, H_7_); 8.21 (s, 1H, H_9_). **IR**—νNH 3473 and 3417 cm^−1^; νCH 2640cm^−1^; νC = C 1606 cm^−1^; νC = N 1587 cm^−1^.

#### 7-Chloro-4-[(2*E*)-2-(4-phenylbutan-2-ylidene)hydrazinyl]quinoline (GPQF-8Q21)

Prepared in accordance with general synthetic procedure, which involved using 7-chloro-4-hydrazinylquinoline (1mmol) and 4-phenylbutan-2-one (1 mmol) as reactants, and a solution of ethanol/acetic acid 25% as the solvent. The reaction was maintained under reflux at 80 °C for 16 h. Beige solid. **Yield** 71%. **M.P**. 161–166 °C. ^**1**^**H-NMR**-(DMSO-*d*_6_)—*δ* = ppm: 2.21 (s, 3H, H_23_); 2.78 (t, 2H, *J* = 7.5 Hz, H_15_); 2.98 (t, 2H, *J* = 7.5 Hz, H_14_); 7.13 (d, 1H, *J* = 9 Hz, H_3_); 7.19–7.23 (m, 2H, H_18_, H_20_); 7.30–7.52 (m, 3H, H_17_, H_19_, H_21_); 7.77 (dd, 1H, *J*_1_ = 9 Hz, J_2_ = 3 Hz H_7_); 7.95 (s, 1H, H_9_); 8.53 (d, 1H, *J* = 9 Hz, H_6_); 8.64 (d, 1H, *J* = 9 Hz, H_2_). ^**13**^**C-NMR**-(75 MHz, DMSO-d_6_)—*δ* = ppm: 17.67 (C_23_); 31.03 (C_15_); 31.48 (C_14_); 100.18 (C_3_); 111.24 (C_9_); 118.14 (C_5_); 119.76 (C_16_); 125.97 (C_19_); 126.15 (C_21_); 126.52 (C_17_); 128.35 (C_7_); 128.39 (C_6_); 137.75 (C_8_); 139.78 (C_13_); 141.07 (C_4_); 143.54 (C_10_); 152.19 (C_2_). **IR**—νNH 3421cm^−1^; νCH 3055cm^−1^; νC = C 1606 cm^−1^; νC = N 1554 cm^−1^.

#### 4-[(2*E*)-2-(butan-2-ylidene)hydrazinyl]-7-chloroquinoline (GPQF-8Q22)

Prepared using 7-chloro-4-hydrazinylquinoline (1mmol) and propan-2-one (1 mmol), in the presence of a solution of ethanol/acetic acid 25%, and left at room temperature for 24 h. Light yellow solid. **Yield** 77%. **M.P**. 71–74 °C. ^**1**^**H-NMR**-(CDCl_3_)—*δ* = ppm: 1.14 (t, 3H, *J* = 6Hz, H_15_); 2.09 (s,3H, H_17_); 2.33–2.41 (m, 2H, H_14_); 7.22 (d, 1H, *J* = 6Hz, H_3_); 7.51 (d, 1H, *J* = 9Hz, H_7_); 7.86 (s, 1H, H_9_); 8.37(d, 1H, *J* = 9Hz, H_6_); 8.53 (d, 1H, *J* = 6Hz, H_2_) 9.38 (s, 1H, H_11_). ^**13**^**C-NMR**-(75 MHz, DMSO-d_6_)—*δ* = ppm: 10.72 (C_15_); 14.56 (C_17_); 32.31 (C_14_); 102.12 (C_3_); 115.69 (C_5_); 120.39 (C_9_); 125.64 (C_6_ and C_7_); 134.90 (C_8_ and C_13_); 146.92 (C_4_); 161.88 (C_10_); 177.40 (C_2_). **IR**—νNH 3402 cm^−1^; νCH 2966 cm^−1^; νC = C 1610 cm^−1^; νC = N 1577 cm^−1^.

#### 7-Chloro-4-[(2*E*)-2-(pentan-2-ylidene)hydrazinyl]quinoline (GPQF-8Q23)

Prepared using 7-chloro-4-hydrazinylquinoline (1mmol) and butan-2-one (1 mmol), in the presence of a solution of ethanol/acetic acid 25%. The reaction was mainteined at room temperature for 24 h. Yellow solid. **Yield** 51%. **M.P**. 250 °C (decomposition). ^**1**^**H-NMR**-(DMSO-*d*_6_)—*δ* = ppm: 0.96 (t, 3H, *J* = 7.5Hz, H_16_); 1.63–1.40 (m, 2H, H_15_); 2.21 (s, 3H, H_18_); 2.43 (t, 2H, *J* = 7.5Hz, H_14_); 7.24 (d, 1H, *J* = 6Hz, H_3_); 7.81 (d, 1H, *J* = 9Hz, H_7_); 8.12 (s, 1H, H_9_); 8.58 (d, 1H, *J* = 9Hz, H_6_); 8.81 (d,1H, *J* = 9Hz, H_2_). **IR**—νNH 3419 cm^−1^, νCH 2956–2648 cm^−1^; νC = C 1610 cm^−1^; νC = N 1587 cm^−1^.

#### 7-Chloro-4-[(2*E*)-2-(3,3-dimethylbutan-2-ylidene)hydrazinyl]quinoline (GPQF-8Q25)

Prepared using 7-chloro-4-hydrazinylquinoline (1mmol) and 3,3-dimethybutan-2-one (1 mmol), with a solution of ethanol/acetic acid 25% as the solvent. The reaction was maintained at room temperature for 24 h. Light yellow solid. **Yield** 59%. **M.P**. 228–232 °C. ^**1**^**H-NMR**-(DMSO-*d*_6_)—*δ* = ppm: 1.23 (s, 9H, H_19_, H_18_, H_15_); 2.23 (s, 3H, H_17_); 7.27 (d, 1H, *J* = 9Hz, H_7_); 7.79 (d, 1H, *J* = 9Hz, H_3_); 8,17 (s, 1H, H_9_), 8.58 (d, 1H, *J* = 6Hz, H_6_); 8.83 (d, 1H, *J* = 9Hz, H_2_). ^**13**^**C-NMR**-(75 MHz, DMSO-d_6_)—*δ* = ppm: 13.78 (C_17_); 27.47 (C_15_, C_18_ and C_19_); 39.35 (C_14_); 100.18 (C_3_); 114.07 (C_9_); 119.10 (C_5_); 126.56 (C_7_); 126.69 (C_6_); 138.00 (C_8_); 139.15 (C_13_); 142.96 (C_4_); 152.80 (C_10_). **IR**—νNH 3419 cm^−1^; νCH 2970–2648 cm^−1^; νC = C 1608 cm^−1^; νC = N 1585 cm^−1^.

#### 7-Chloro-4-{(2*E*)-2-[1-(4-chlorophenyl)ethylidene]hydrazinyl}quinoline (GPQF-8Q26)

7-Chloro-4-hydrazinylquinoline (1mmol) and 1-(4-chlorophenyl)ethanone (1 mmol) were coupled following the general synthetic procedure. A solution of ethanol/acetic acid 25% was used as the solvent, and the reaction was maintained under reflux at 80 °C for 16 h. Orange solid. **Yield** 67%. **M.P**. 290–292 °C. ^**1**^**H-NMR**-(DMSO-*d*_6_)—*δ* = ppm: 2.62 (s, 3H, H_14_); 7.58 (d, 3H, *J* = 9Hz, H_3_, H_17_, H_21_); 7.86 (d, 1H, *J* = 9Hz, H_7_); 8.03 (d, 2H, *J* = 9Hz, H_18_, H_2_0); 8.08 (s, 1H, H_9_); 8.66 (d, 1H, *J* = 6Hz, H_6_); 8.82 (d, 1H, *J* = 9Hz, H_2_). **IR**—νNH 3481 and 3414cm^−1^; νCH 2607cm^−1^; νC = C 1622 cm^−1^; νC = N 1587 cm^−1^.

### Biology

#### Animals, parasites, cells, and nematodes

The life cycle of *S. mansoni* (Belo Horizonte strain) is maintained through routine passage through *Biomphalaria glabrata* snails and Swiss mice at the Research Center on Neglected Diseases (Guarulhos University, SP, Brazil). Both rodents and snails were kept under environmentally controlled conditions (25 °C; humidity of 50%), with free access to food and water^16^. Vero cells were obtained from the American Type Culture Collection (ATCC CCL-81; Manassas, VA, USA). Cells were routinely cultured in Dulbecco’s Modified Eagle Medium (DMEM) supplemented with 5% heat-inactivated fetal bovine serum and antibiotics (100 U/mL penicillin and 100 μg/mL streptomycin) at 37 °C in a humidified atmosphere containing 5% CO_2_^17^.

*Caenorhabditis elegans* wild-type (N2 strain), kindly provided by Dr. Carlos E. Winter (University of São Paulo), was routinely cultured at 22 °C on nematode growth medium (NGM) plates seeded with *Escherichia coli* (strain OP50) as a food source according to standard protocols ^18^.

#### In vitro antischistosomal assay

The antischistosomal assay was performed according to the methodology previously described^16,19,20^. Briefly, adult parasites, obtained from infected mice at day 42 post-infection, were incubated in flat bottom 24-well plates (Corning, New York, NY, USA) in RPMI 1640 culture medium (Vitrocell, Campinas, SP, Brazil) supplemented with 5% inactivated fetal calf serum, antibiotics (100 U/mL penicillin and 100 μg/mL streptomycin), and buffered with HEPES 25 mM (Sigma-Aldrich, St. Louis, MO, USA). Compounds were dissolved in dimethyl sulfoxide (DMSO at a final concentration of 0.5% v/v) and tested at 50 μM using PZQ 2 μM and DMSO 0.5% as positive and negative controls, respectively. Parasites were kept for 72 h (37 °C, 5% CO_2_, Panasonic, Osaka, Japan) and their viability was assessed using a Motic AE2000 inverted microscope (Vancouver, Canada)^21^. Each experiment was performed in triplicate and repeated at least three times. The negative control (using the highest concentration of DMSO, i.e., 0.5) and positive control (praziquantel 2 µM) were included.

For the determination of an effective concentration of 50% (EC_50_), compounds were tested using 1:2 serial dilutions from 50 to 3.12 µM as previously described^22,23^. Each concentration was tested in five replicates, and experiments were repeated once.

#### In vitro cytotoxicity assay

The cytotoxicity of the compounds on the Vero cells was determined by thiazolyl blue tetrazolium bromide (MTT) assay (Sigma-Aldrich) as previously described^21,24^. Briefly, cells were seeded using the density of 2 × 104 cells/well in a 96-well culture plate (Corning) and incubated with compounds (started at 200 µM and followed a 1:2 dilution series) at 37 °C and 5% CO_2_ (Panasonic). After 72 h, MTT solution was added to each well and the absorbance at 595 nm was read on a spectrophotometer (Epoch, BioTek Instruments, Winooski, VT, USA). The selectivity indices (SI) were calculated by dividing the 50% cytotoxic concentration (CC_50_) obtained on cells with 50% effective concentration (EC_50_) values determined on *S. mansoni*^25^.

#### In vivo antiparasitic studies in an animal model of schistosomiasis

In vivo studies in *S. mansoni*-infected mice were performed according to standard protocols^20,26^. Rodents (3 weeks old) were infected subcutaneously with 80 *S. mansoni* cercariae each and animals were randomly divided into experimental groups (five mice per group). Compounds were dissolved in 2% ethanol in water (v/v) and tested at a single oral dose (400 mg/kg) administered 42 days post-infection^27^. For comparison, praziquantel (400 mg/kg) and a corresponding amount of vehicle were administered to groups of five schistosomes-infected animals in the same period^28,29^. Two weeks after treatment, animals were placed in a chamber and euthanized by introducing 100% medical grade CO_2_ and therapeutic efficacy was based on the following aspects: (i) the number of schistosomes collected by portal perfusion (worm burden); (ii) quantitative fecal examination (egg burden determined by Kato-Katz technique), and (iii) eggs in tissue (egg burden in intestine determined by oogram method). as previously described^30,31^. The percentage of worm and egg reduction was calculated by means of the following equation: % reduction = [(value of untreated control group − value of treatment group)/value of untreated control group] × 100%^32^. The nonparametric Kruskal–Wallis test was used to compare the medians of the responses between the treatment and control groups using the GraphPad Prism software (version 8.0; CA, USA). Differences were considered statistically significant at *P* < 0.05^9^.

#### In vitro toxicity assay in *C. elegans* model

For the drug assessment, the age-synchronized L1 stage was cultivated on standard NGM plates^18^. When at the L4 stage, approximately 25 nematodes were transferred to each well of a 96-wells NGM plates containing the drug of interest at the desired concentration (10, 25, 50, 100, and 200 μM) along with a positive control (levamisole 10 μM) and a negative control (DMSO 0.5%). Nematodes were kept at 22 °C for 24 h, and their viability was verified microscopically using a Motic AE2000 inverted microscope (Vancouver, Canada) equipped with an ultra-high definition (UHD) camera and with a 48-inch 4K-UHD monitor system (LG Electronics, São Paulo, Brazil)^33^. The worm viability was calculated by counting mobile worms and those considered dead which showed no movement on physical stimuli with a fine needle^34^.

### Ethical approval

Animal studies are reported in compliance with the ARRIVE guidelines. The protocol for experimental design was reviewed and approved by the Committee for the Ethical Use of Animals in Experimentation at Guarulhos University, (Guarulhos, SP, Brazil; protocol ID 47/20) in accordance with the Guidelines for Care and Use of Laboratory Animals as stipulated by Brazilian laws.

### Supplementary Information

Supplementary Figures.

## Data Availability

The data pertinent to this study is incorporated within the manuscript. The raw data supporting the findings can be made available by the corresponding author upon a reasonable request.
