# Supplementary material for: Targeting the Schistosoma mansoni nutritional mechanisms to design new antischistosomal compounds
Source: Sci Rep. 2023 Nov 13;13:19735. doi: 10.1038/s41598-023-46959-3 (PMC10643403; doi:10.1038/s41598-023-46959-3)
Supplement: Supplementary file 1 — Supplementary Figures. [file 41598_2023_46959_MOESM1_ESM.docx]

**“Targeting the *Schistosoma mansoni* nutritional mechanisms to design new schistosomicidal compounds”**

Thaís F.A. Pavani^1,2^; Maria E. Cirino^3^; Thainá R. Teixeira^3^; Josué de Moraes^3^; Daniela G.G. Rando^1*^.

1. Grupo de Pesquisas Químico-Farmacêuticas – GPQFfesp – Universidade Federal de São Paulo, Instituto de Ciências Ambientais, Químicas e Farmacêuticas, Departamento de Ciências Farmacêuticas, Diadema-SP, Brazil

2. Curso de Pós-Graduação em Biologia Química da Universidade Federal de São Paulo, Instituto de Ciências Ambientais, Químicas e Farmacêuticas, Diadema-SP, Brazil.

3. Núcleo de Pesquisas em Doenças Negligenciadas – NPDN – Universidade de Guarulhos, Guarulhos-SP, Brazil

*Address correspondence to this author at the Department of Pharmaceutical Sciences from Federal University of São Paulo – UNIFESP, Rua São Nicolau, 210, 2^o^ andar, Centro, Diadema – São Paulo, Brazil, Zip Code: 09913-030, Tel +55 11 3385-4137, ramal 3578.

**Supplementary information**

**Figure S1.** ^1^H-NMR of **7-chloro-4-hydrazinylquinoline**.

**
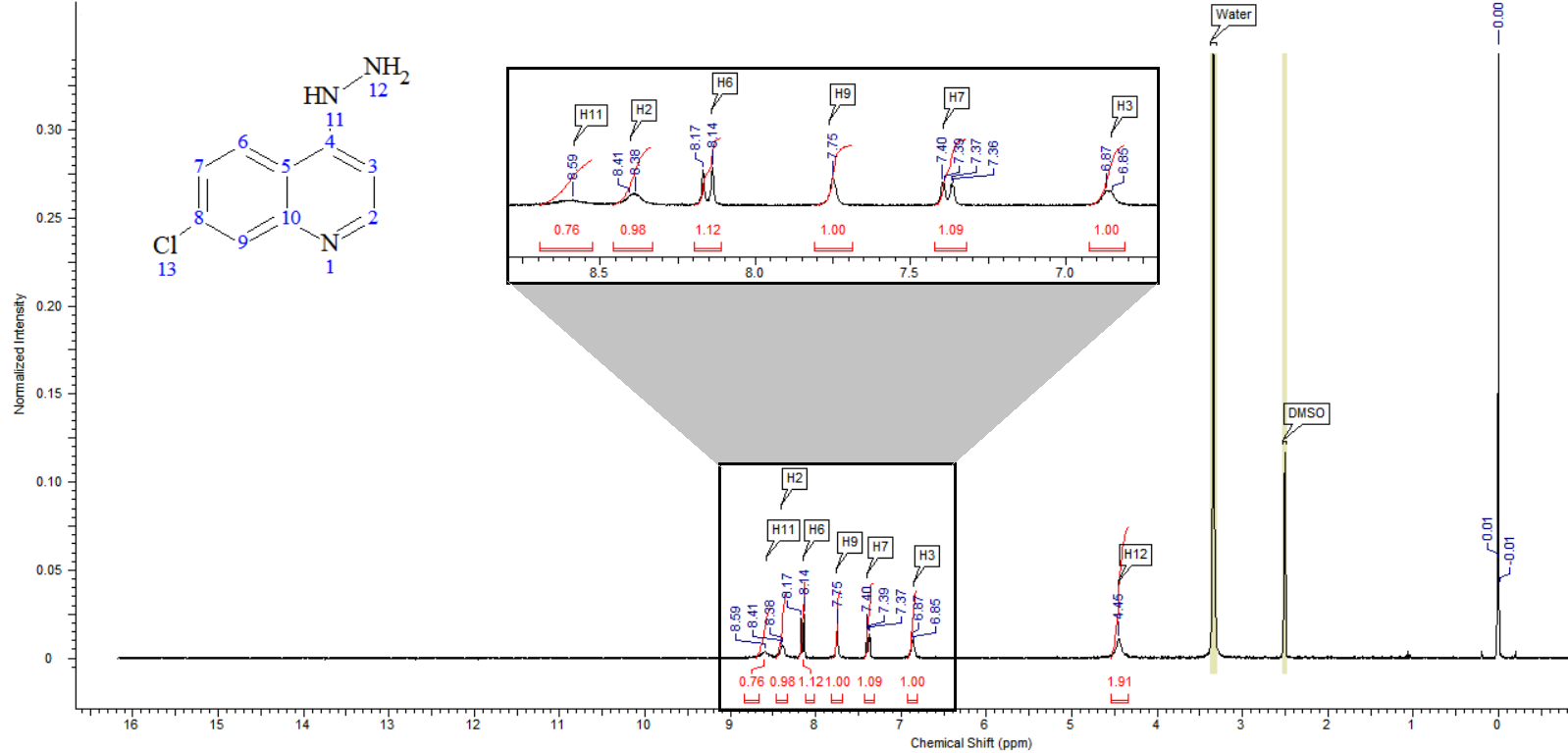
**

**Figure S2**. IV of **7-chloro-4-hydrazinylquinoline**.

**
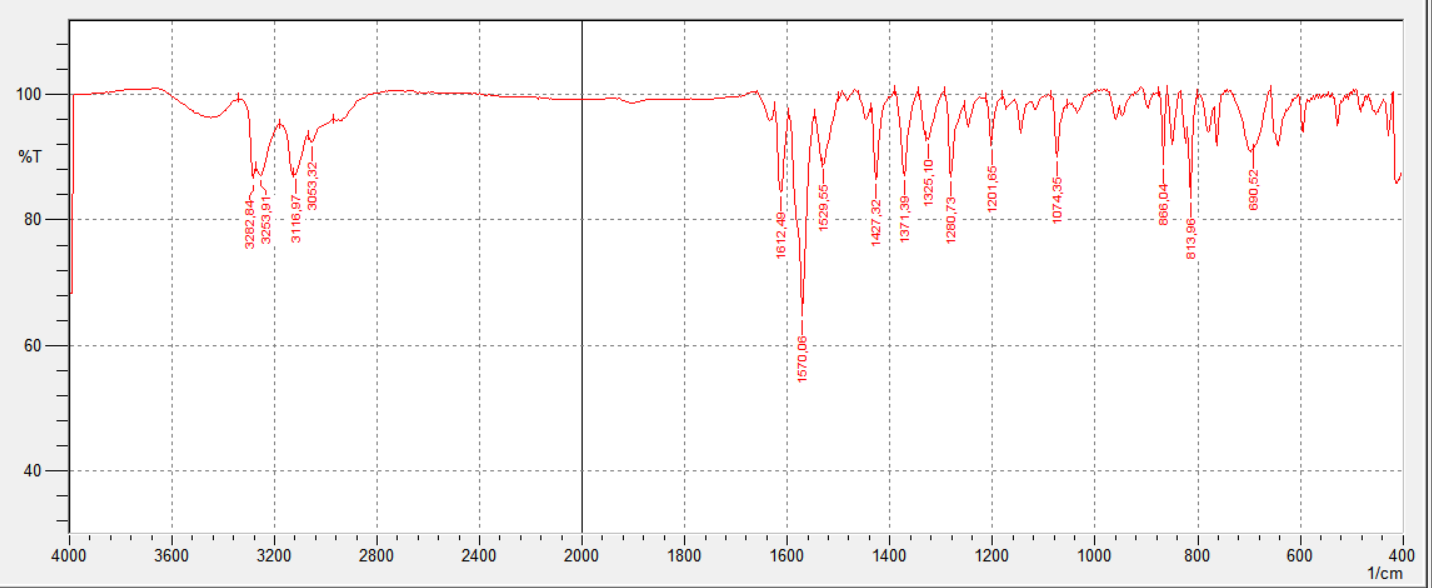
**

**Figure S3.** ^1^H-NMR of compound **GPQF-8Q1.**

**
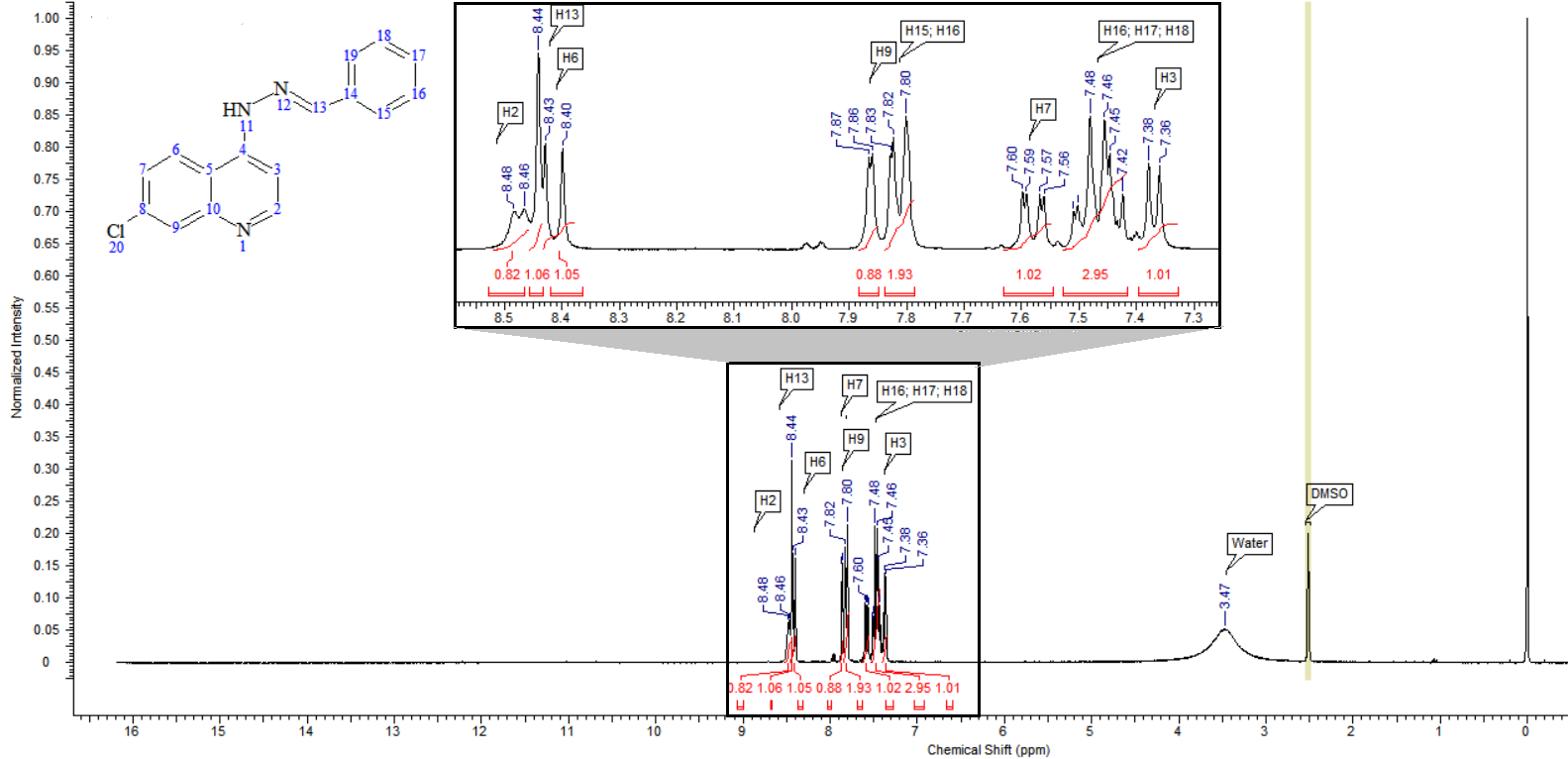
**

**Figure S4**. IV of compound **GPQF-8Q1.**


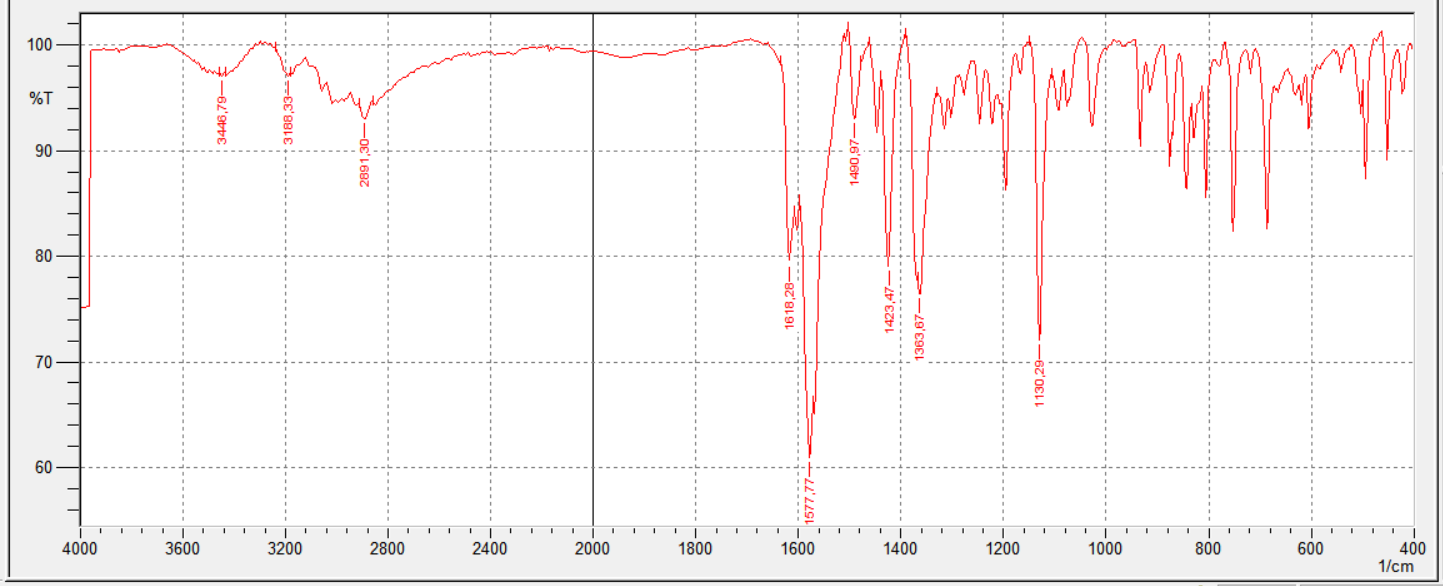


**Figure S5.** ^1^H-NMR of compound **GPQF-8Q2.**

**
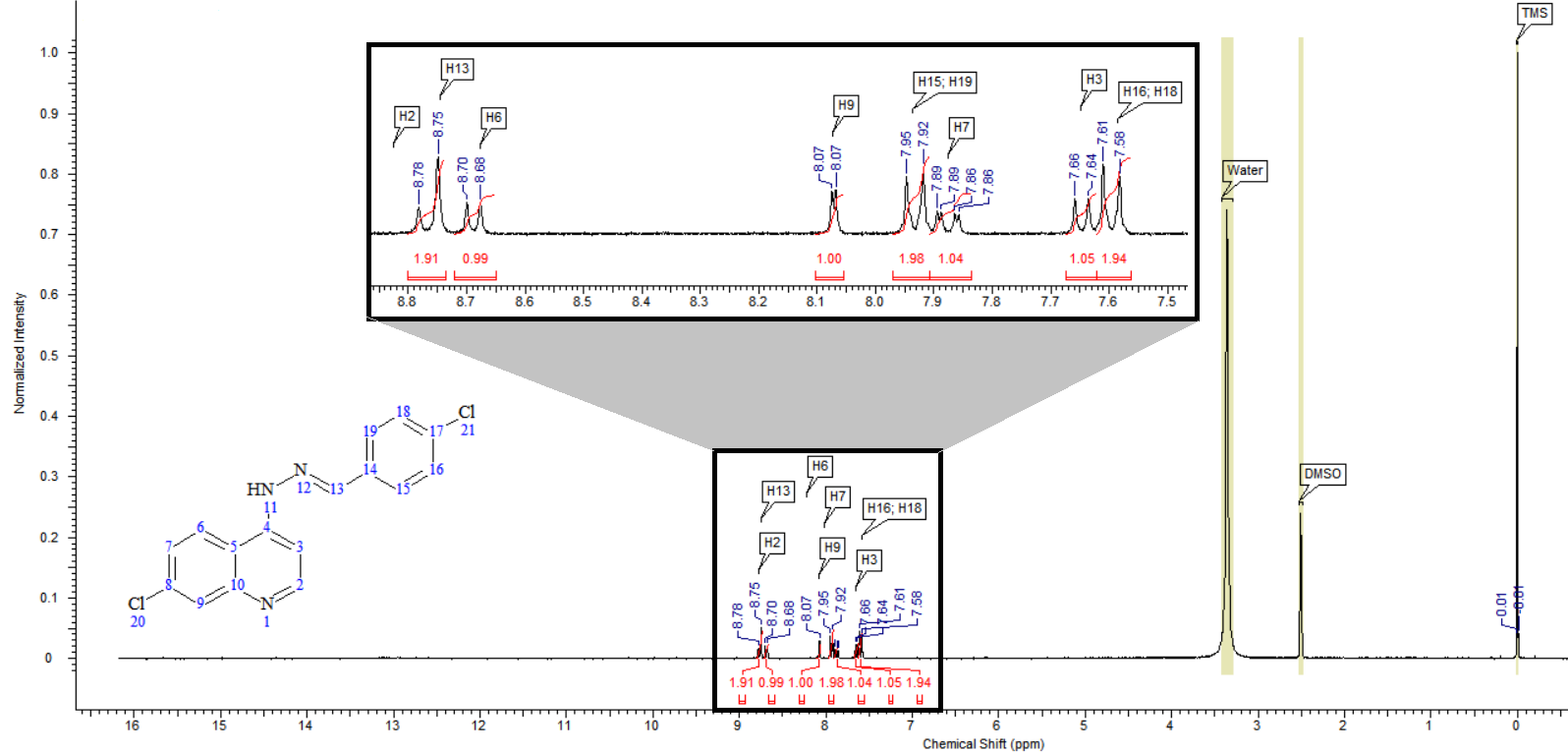
**

**Figure S6**. IV of compound **GPQF-8Q2.**

**
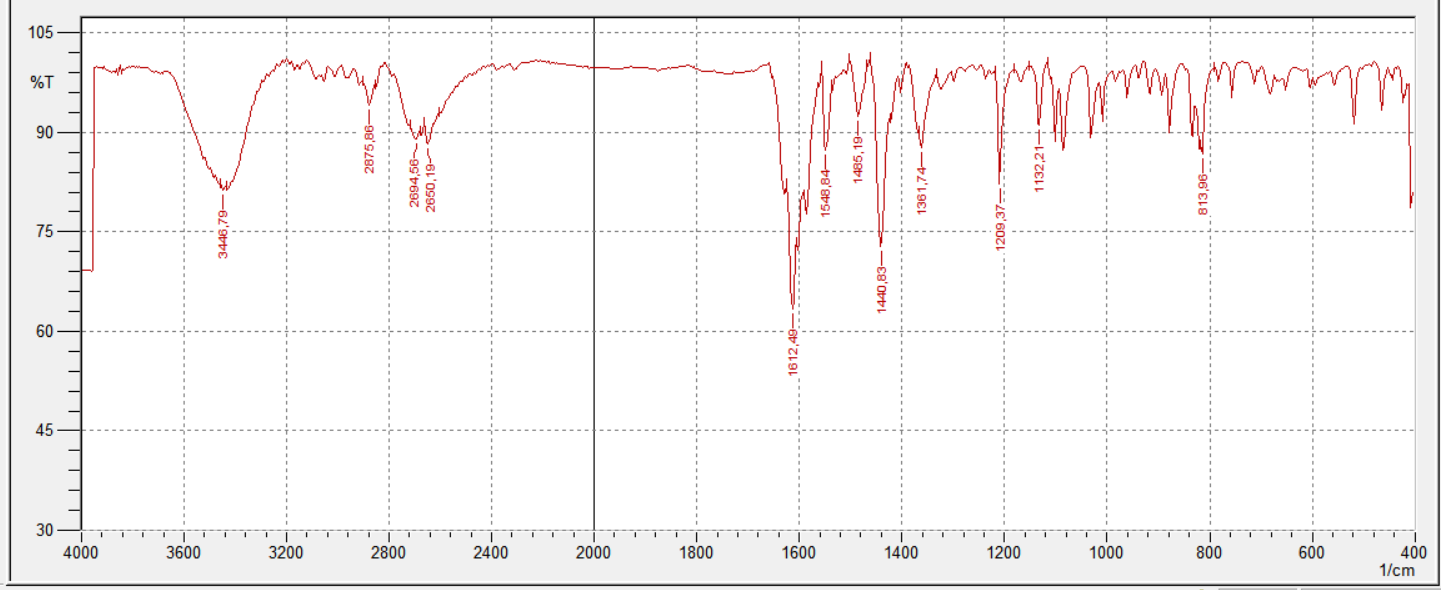
**

**Figure S7.** ^1^H-NMR of compound **GPQF-8Q3**

**
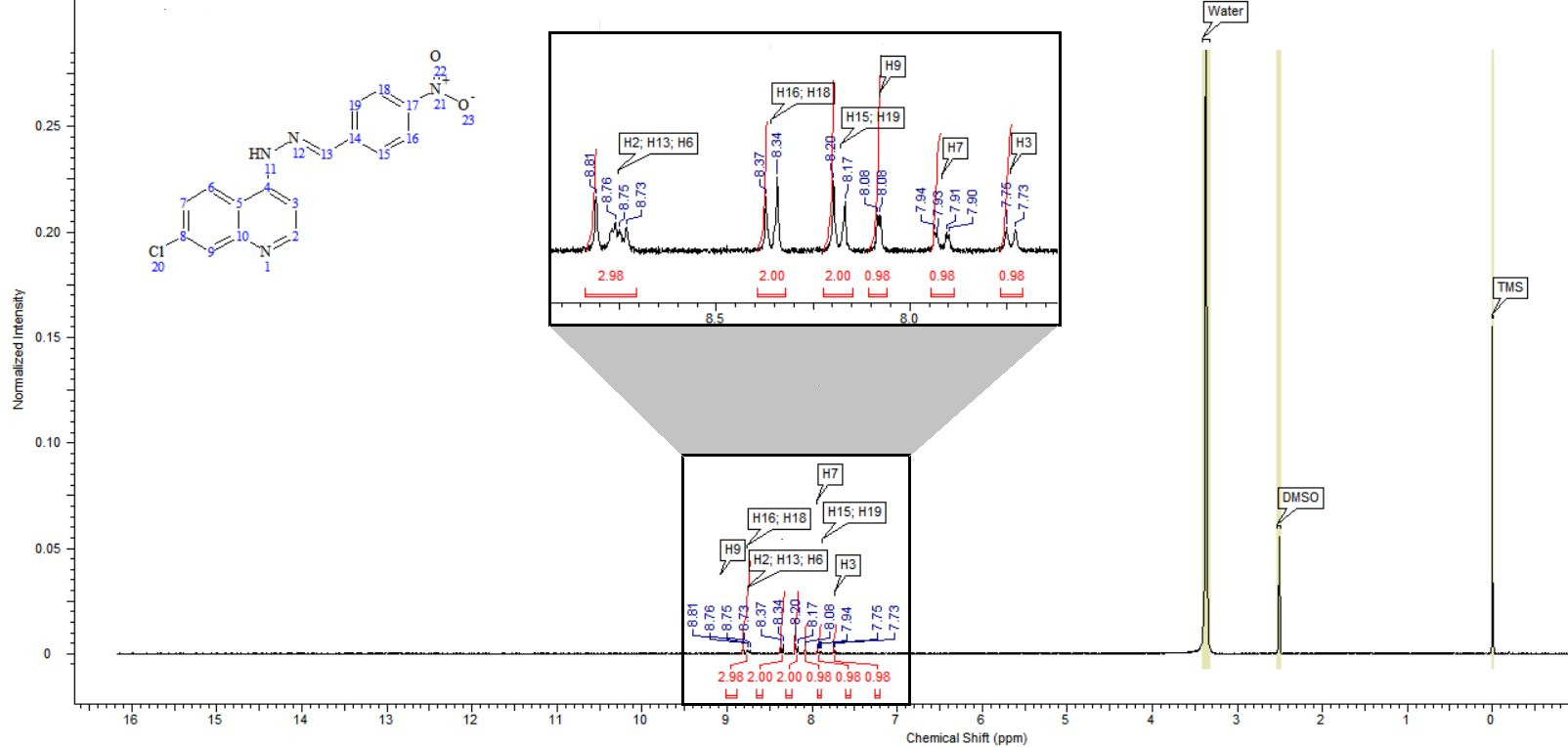
**

**Figure S8**. IV of compound **GPQF-8Q3**

**
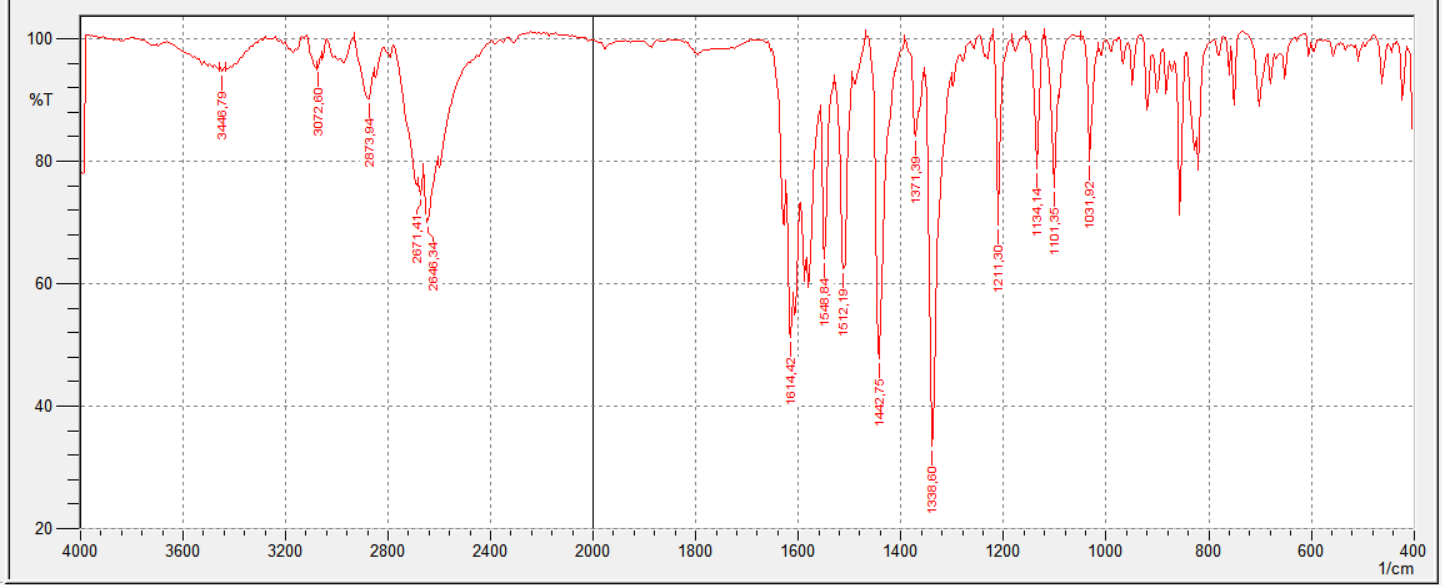
**

**Figure S9.** ^1^H-NMR of compound **GPQF-8Q4**

**
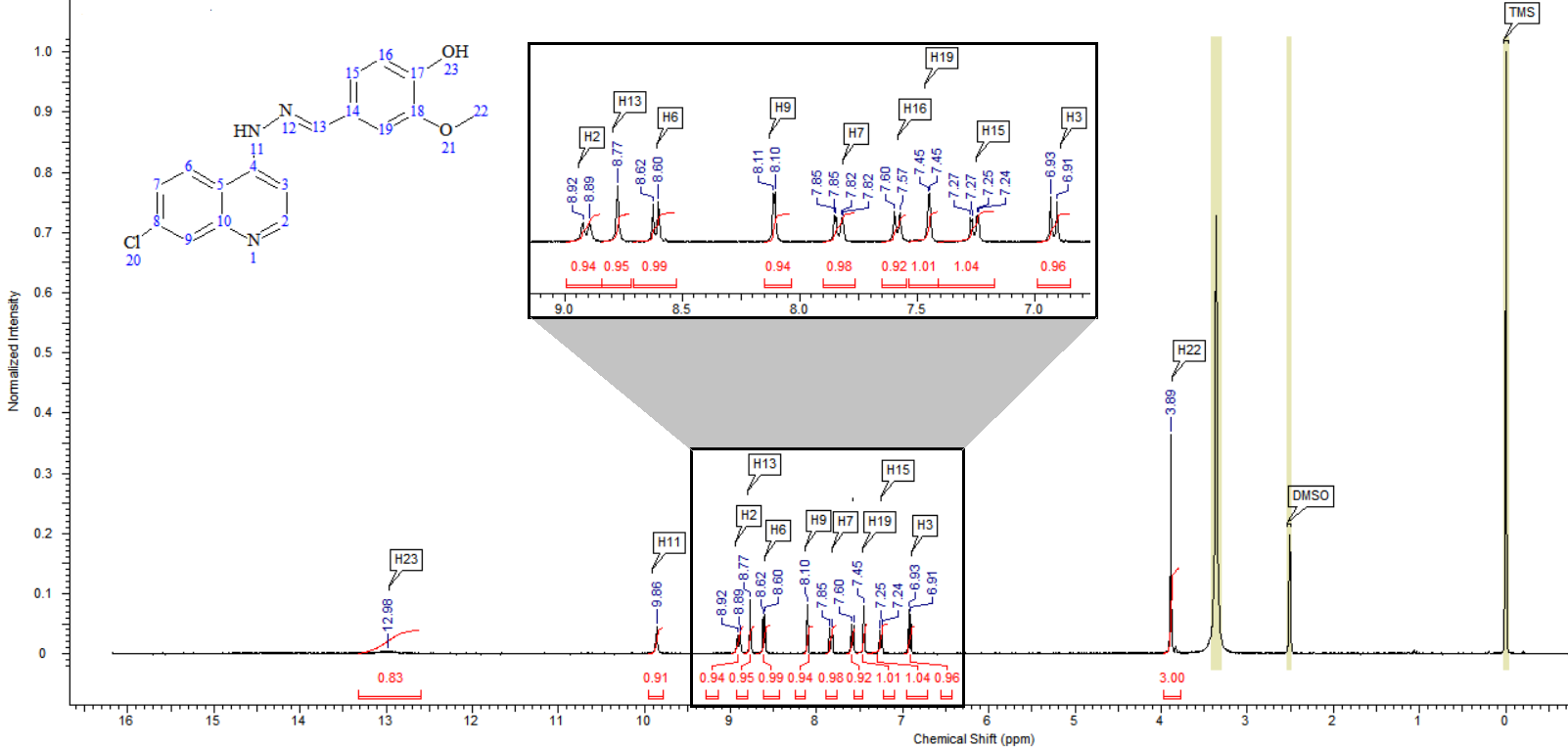
**

**Figure S10**. IV of compound **GPQF-8Q4**

**
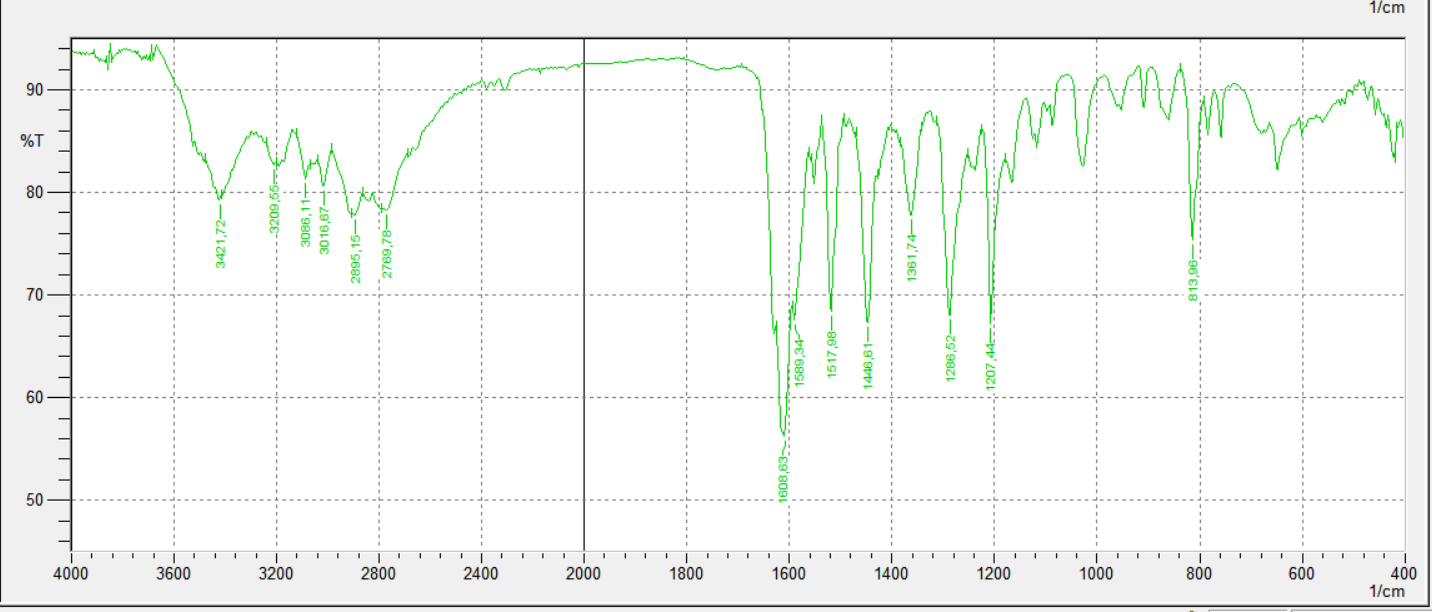
**

**Figure S11.** ^1^H-NMR of compound **GPQF-8Q6**

**
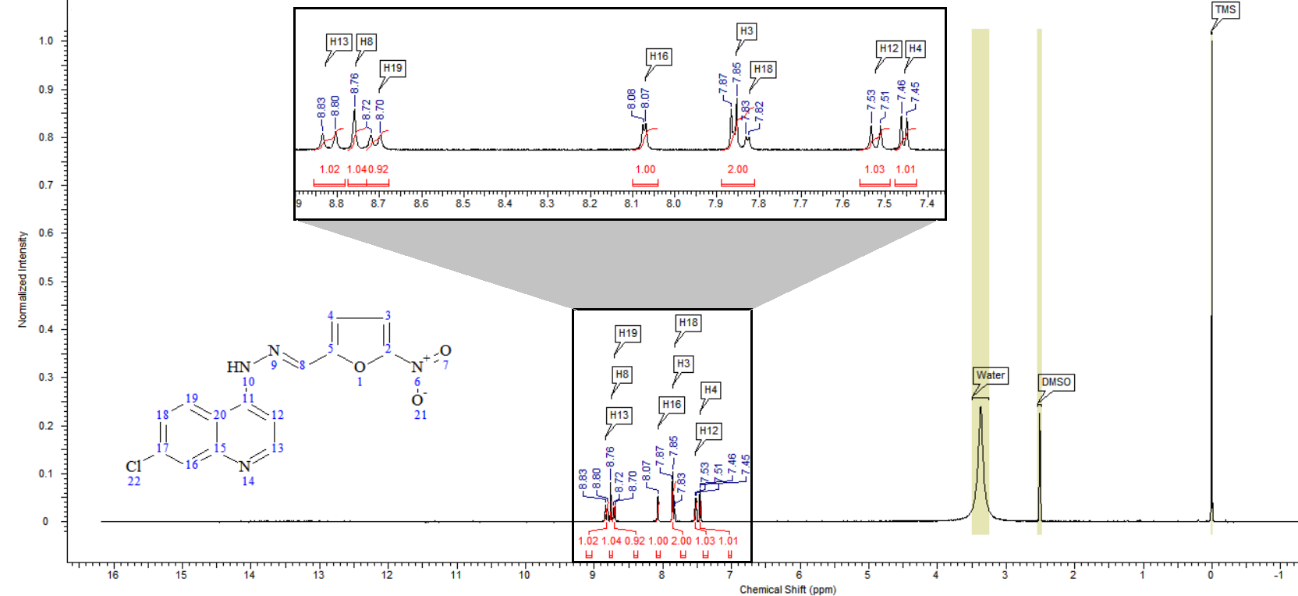
**

**Figure S12**. IV of compound **GPQF-8Q6**

**
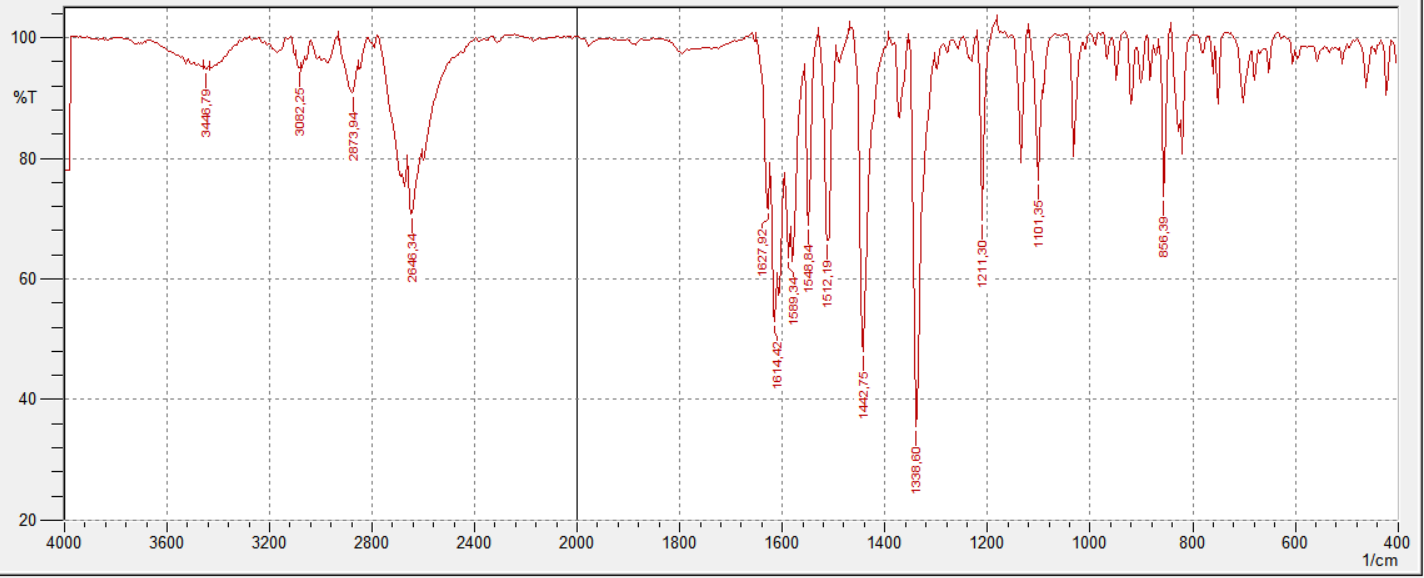
**

**Figure S13.** ^1^H-NMR of compound **GPQF-8Q8**

**
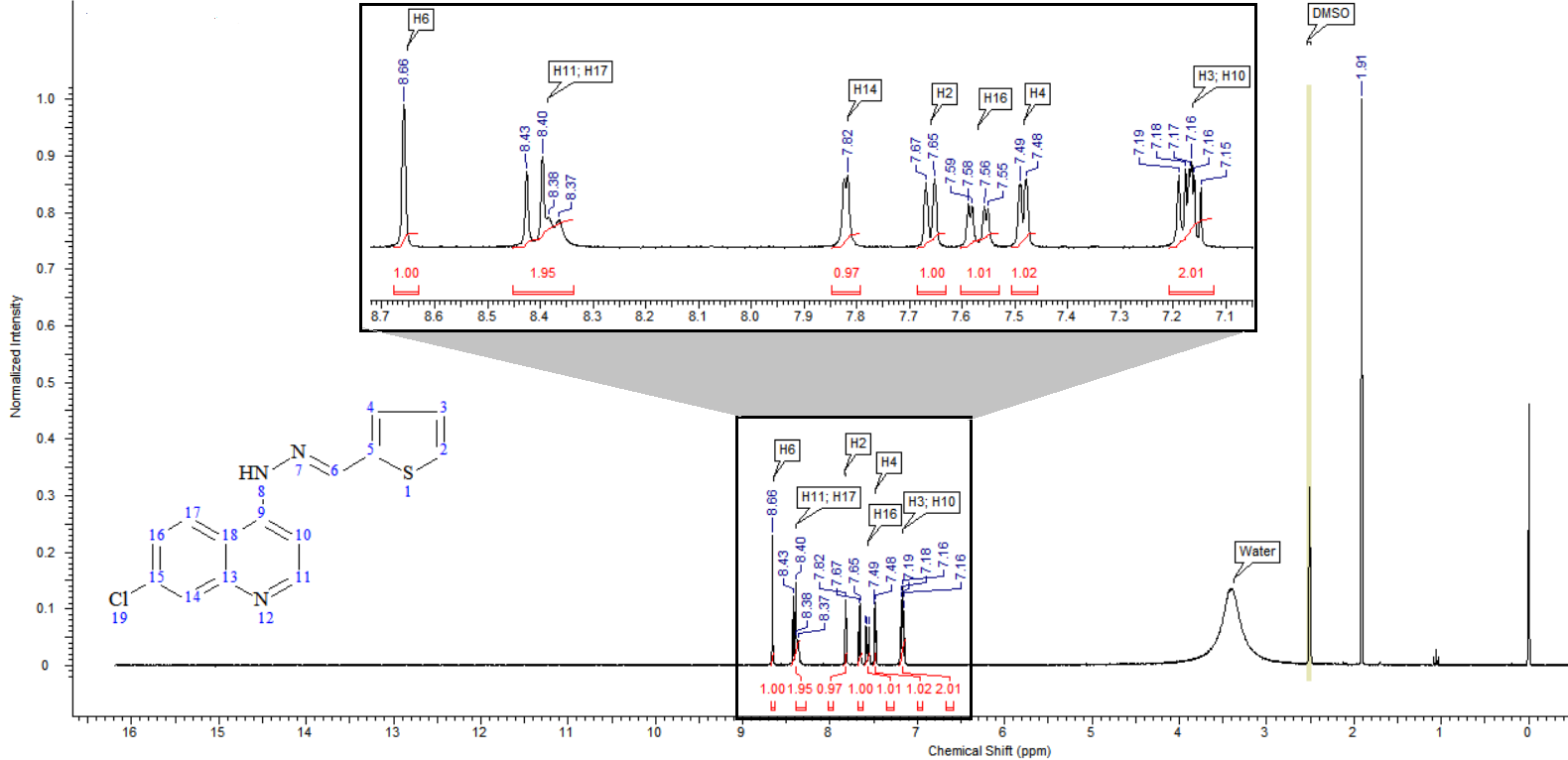
**

**Figure S14**. IV of compound **GPQF-8Q8**

**
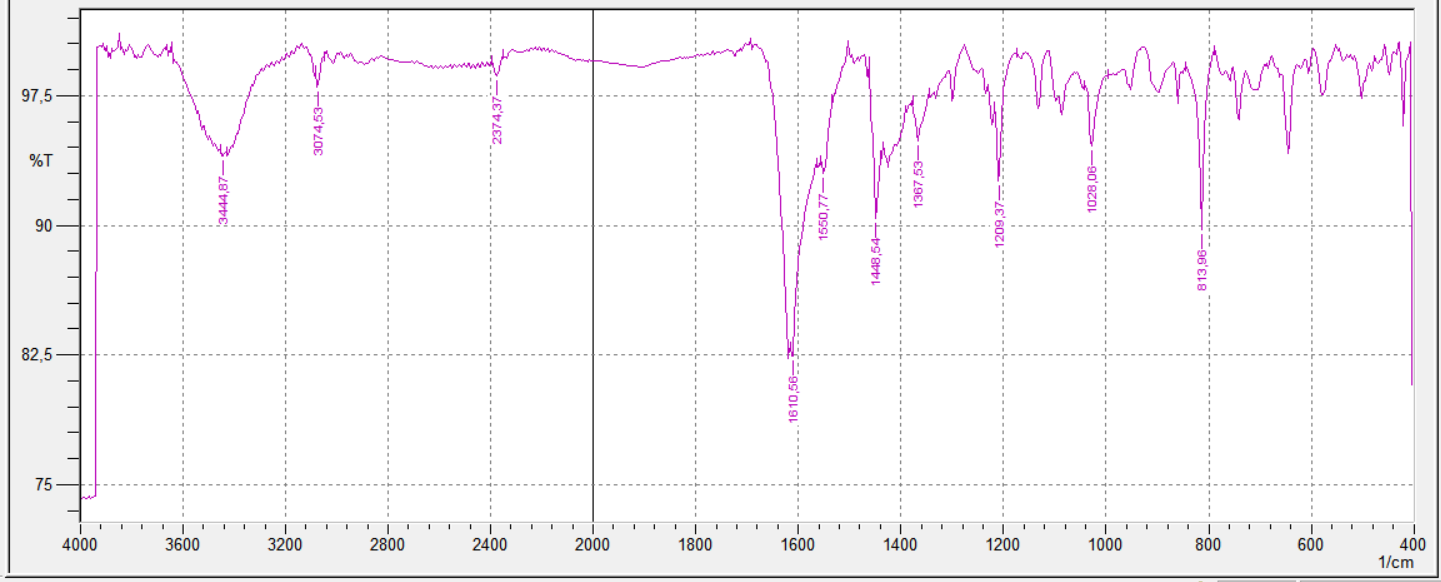
**

**Figure S15.** ^1^H-NMR of compound **GPQF-8Q9**

**
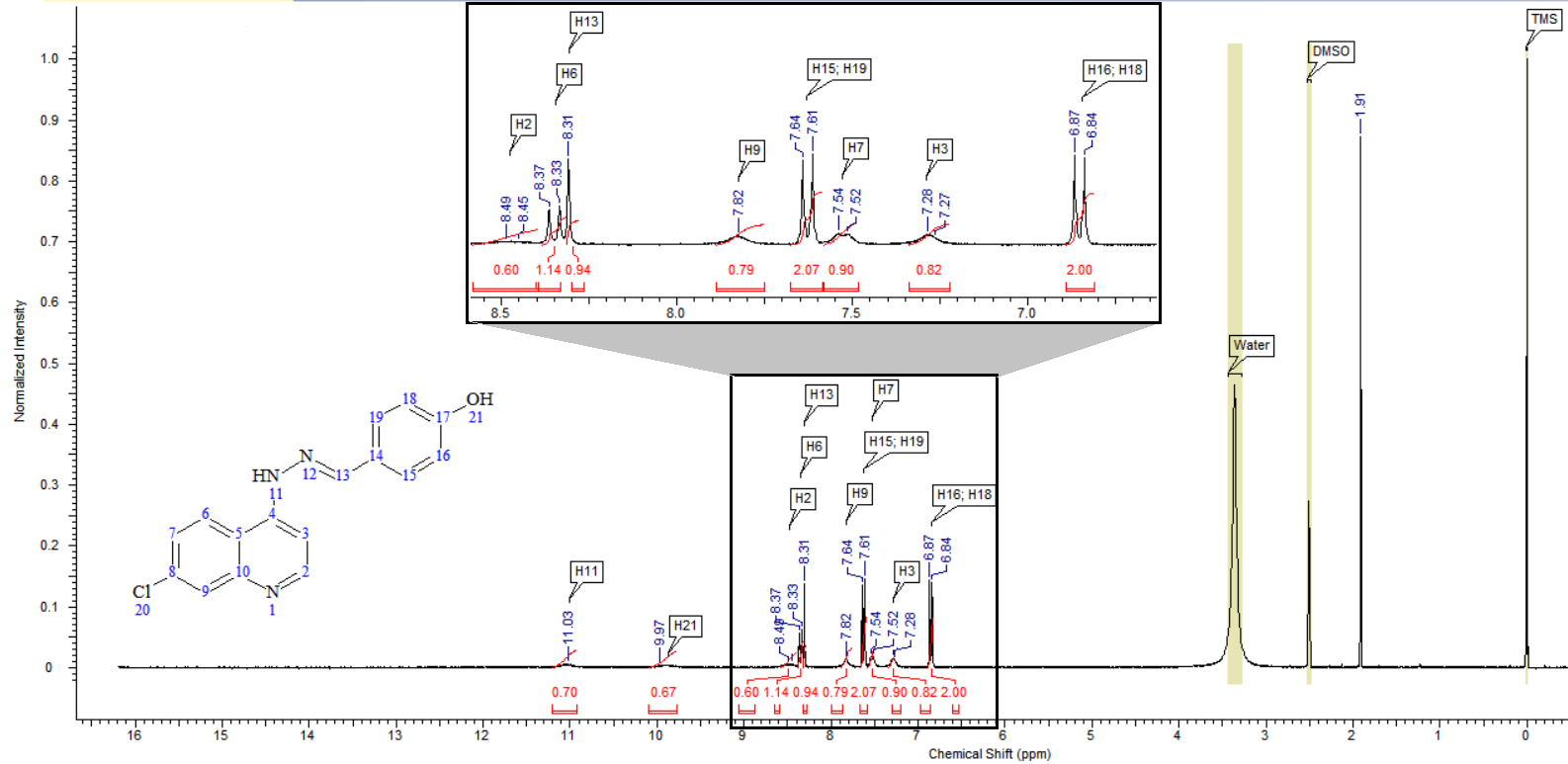
**

**Figure S16**. IV of compound **GPQF-8Q9**

**
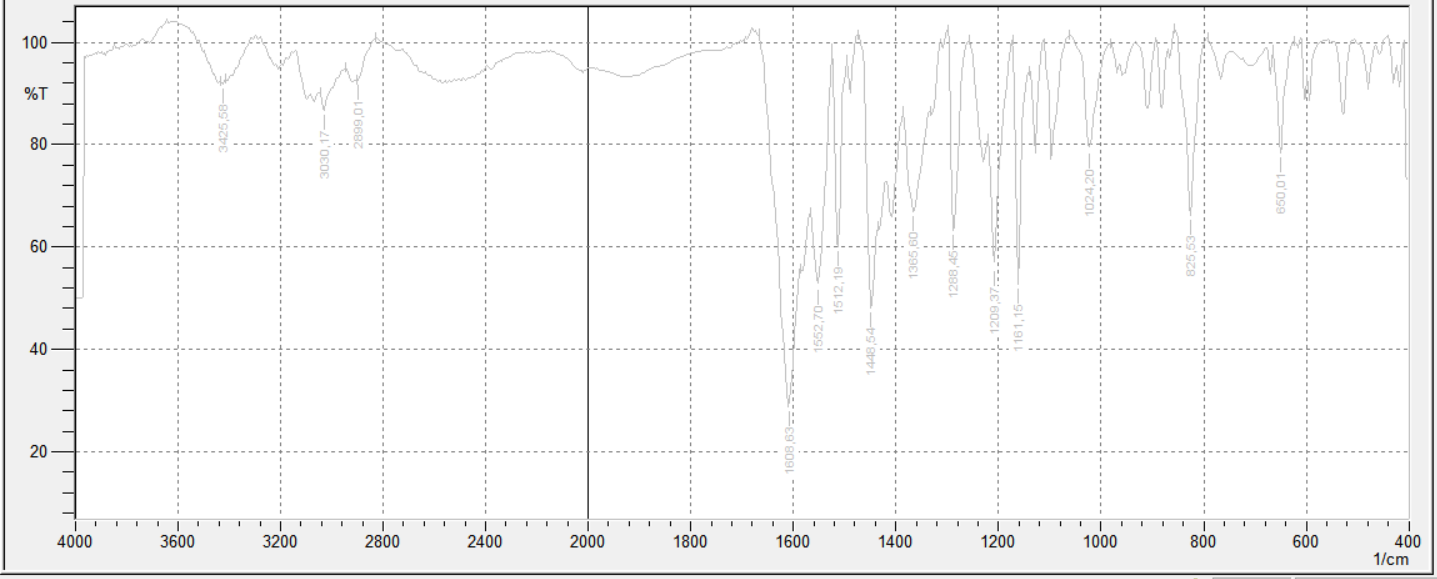
**

**Figure S17.** ^1^H-NMR of compound **GPQF-8Q10**

**
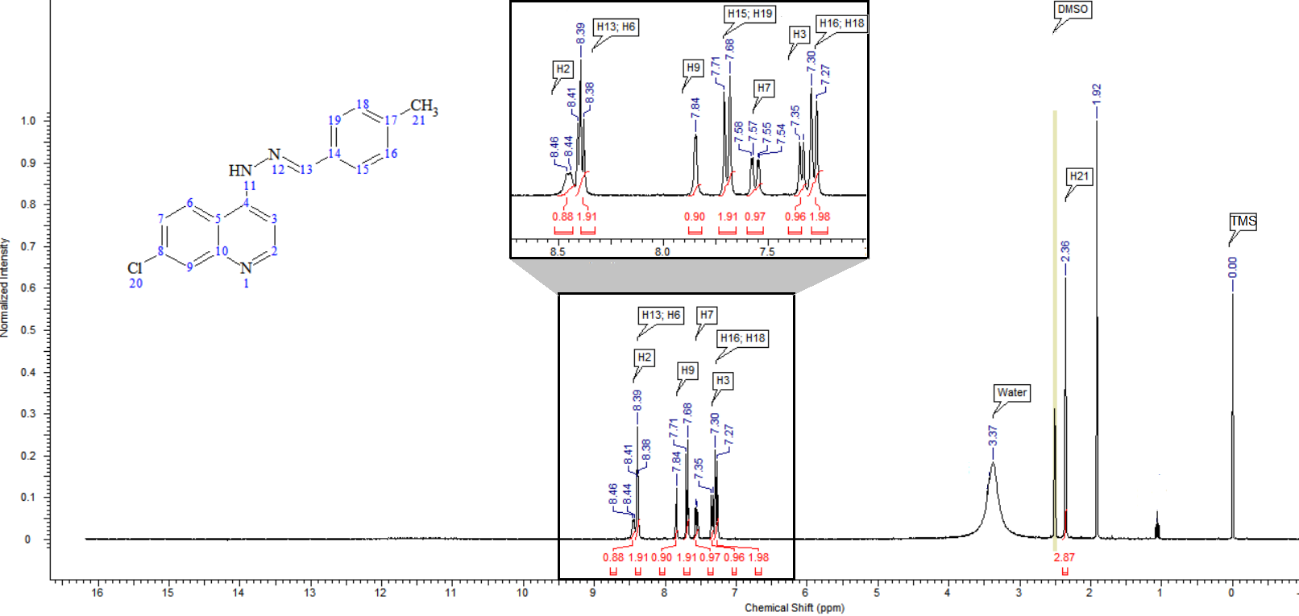
**

**Figure S18**. IV of compound **GPQF-8Q10**

**
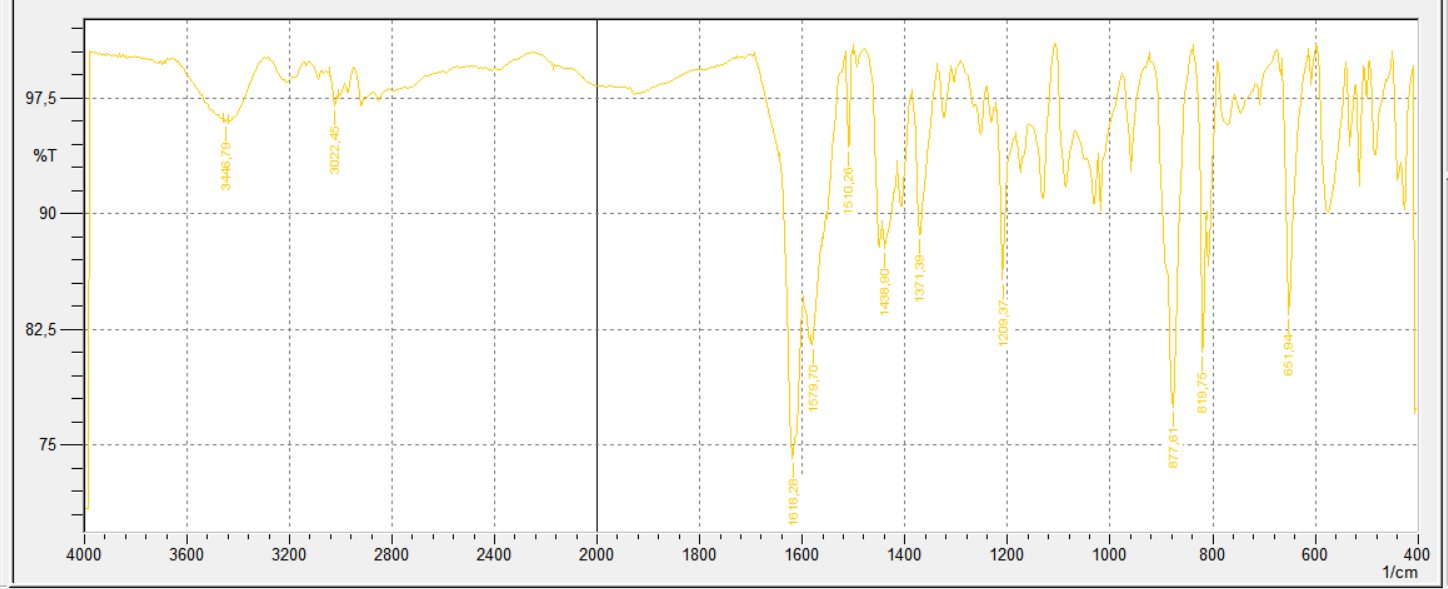
**

**Figure S19.** ^1^H-NMR of compound **GPQF-8Q11**

**
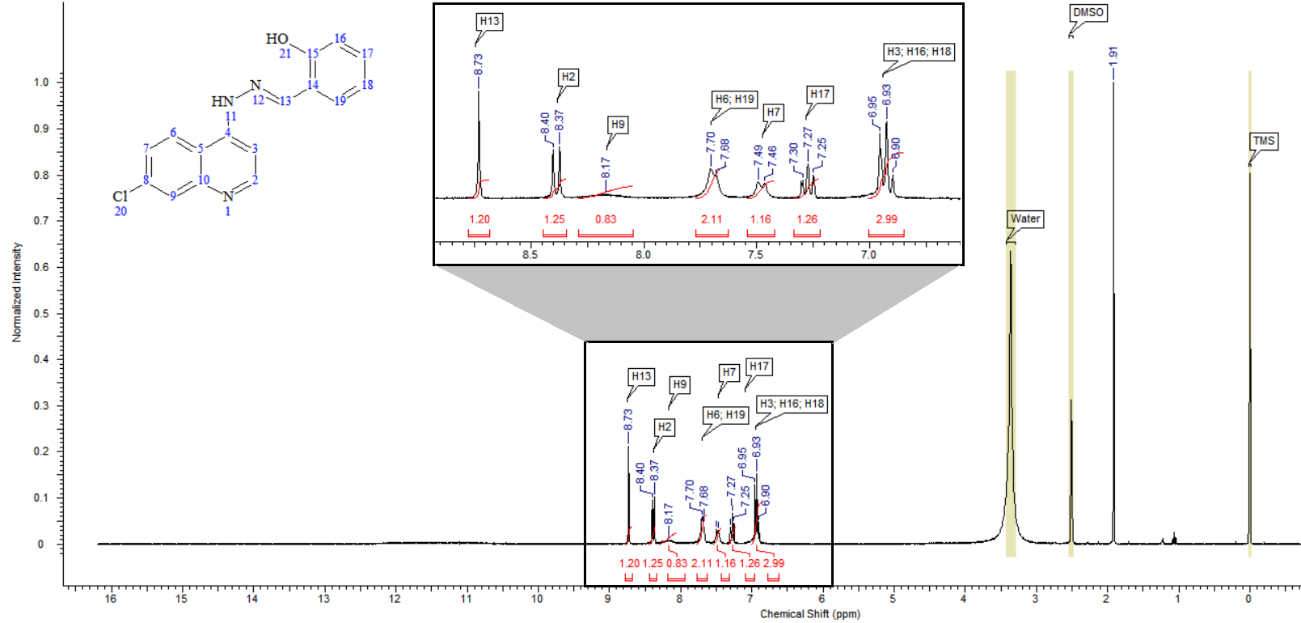
**

**Figure S20**. IV of compound **GPQF-8Q11**

**
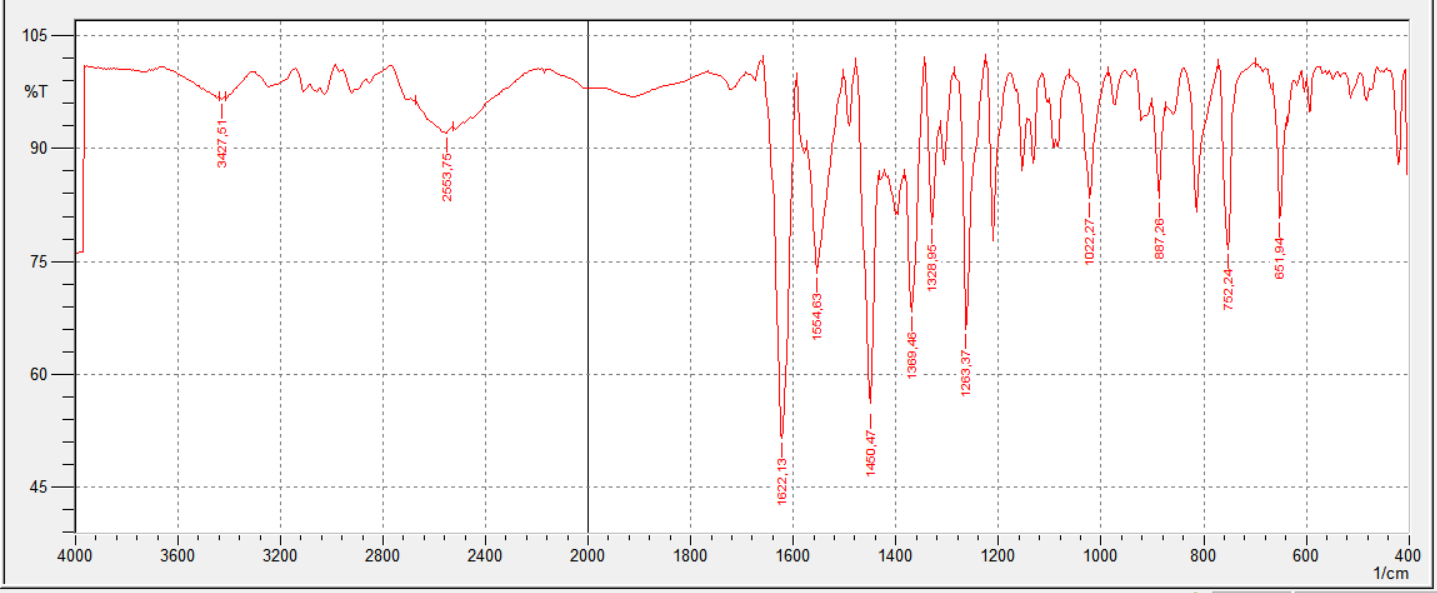
**

**Figure S21.** ^1^H-NMR of compound **GPQF-8Q12**

**
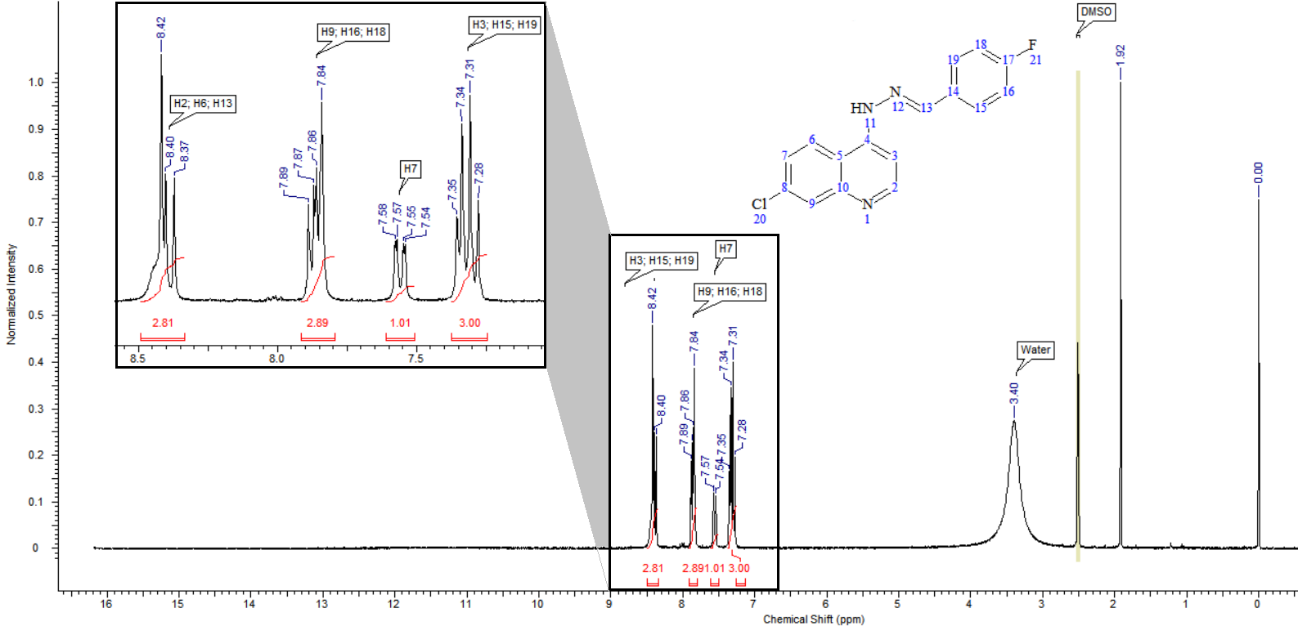
**

**Figure S22**. IV of compound **GPQF-8Q12**

**
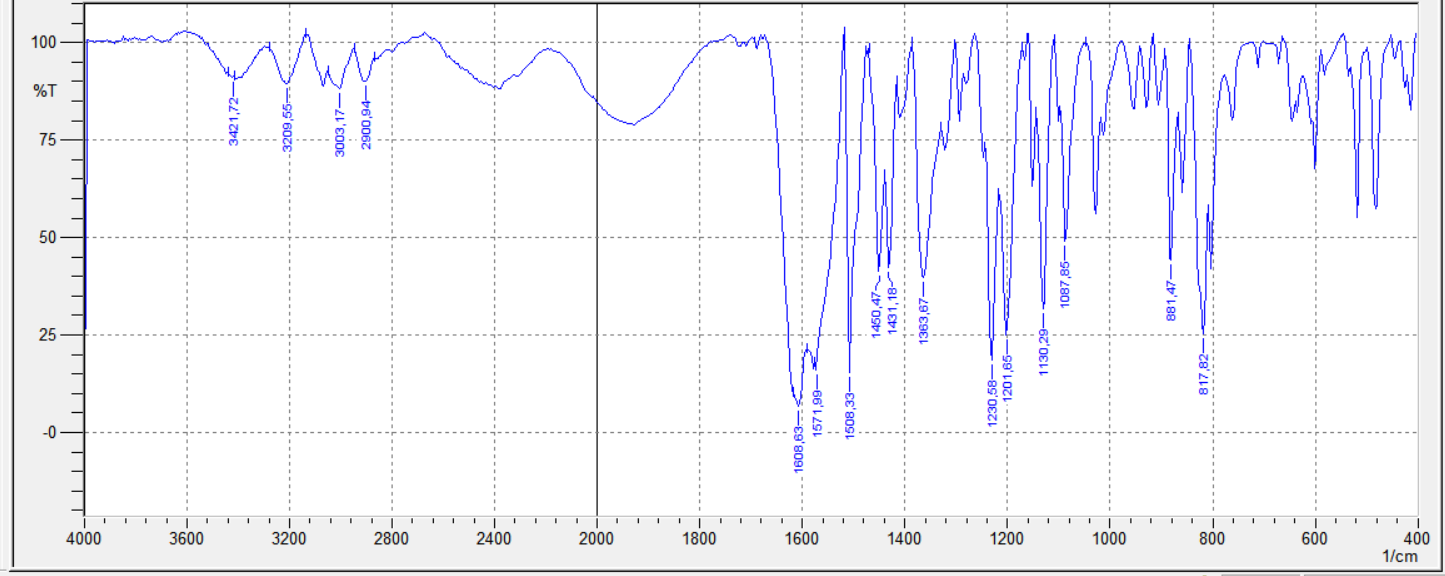
**

**Figure S23.** ^1^H-NMR of compound **GPQF-8Q13**

**
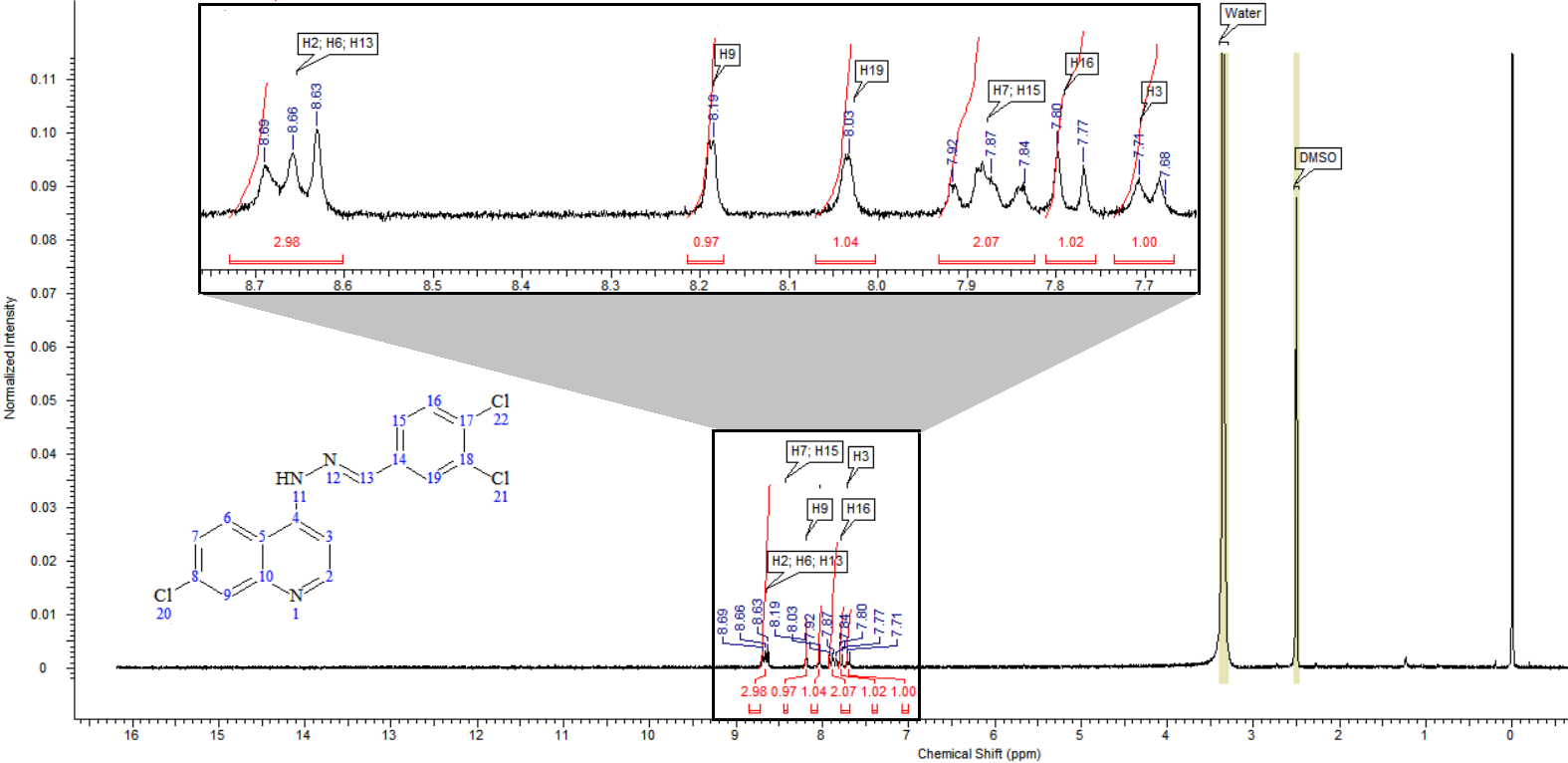
**

**Figure S24**. IV of compound **GPQF-8Q13**

**
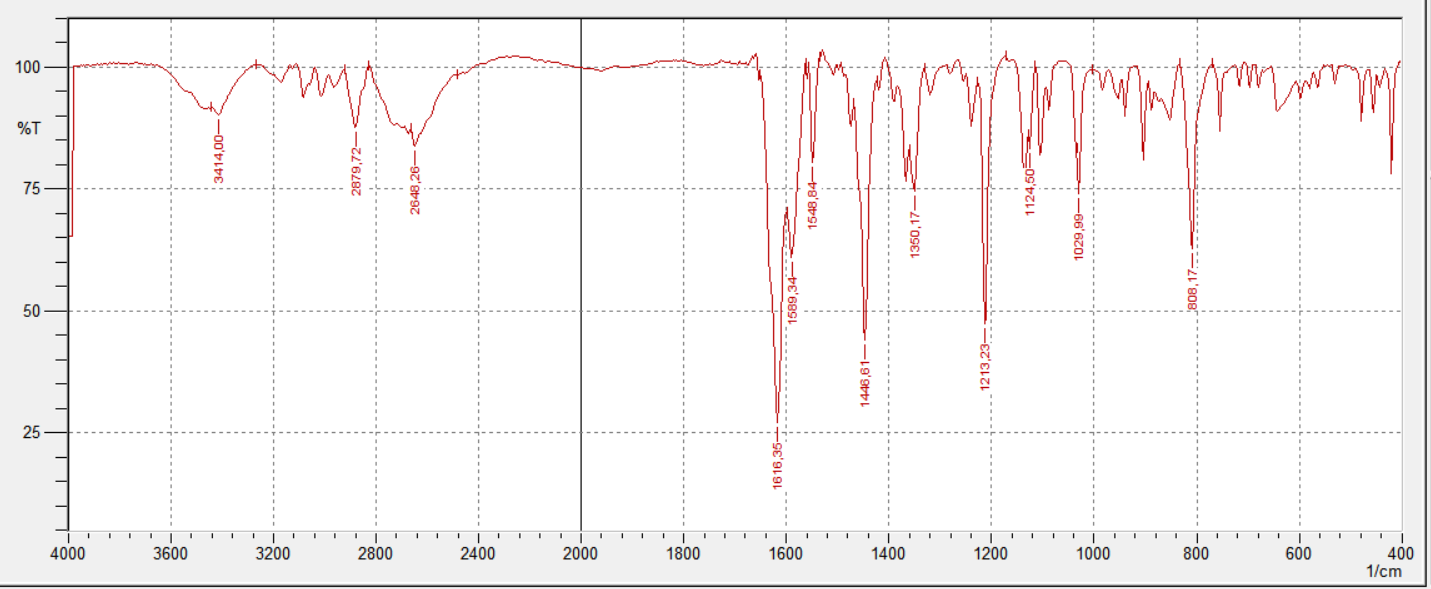
**

**Figure S25.** ^1^H-NMR of compound **GPQF-8Q14**

**
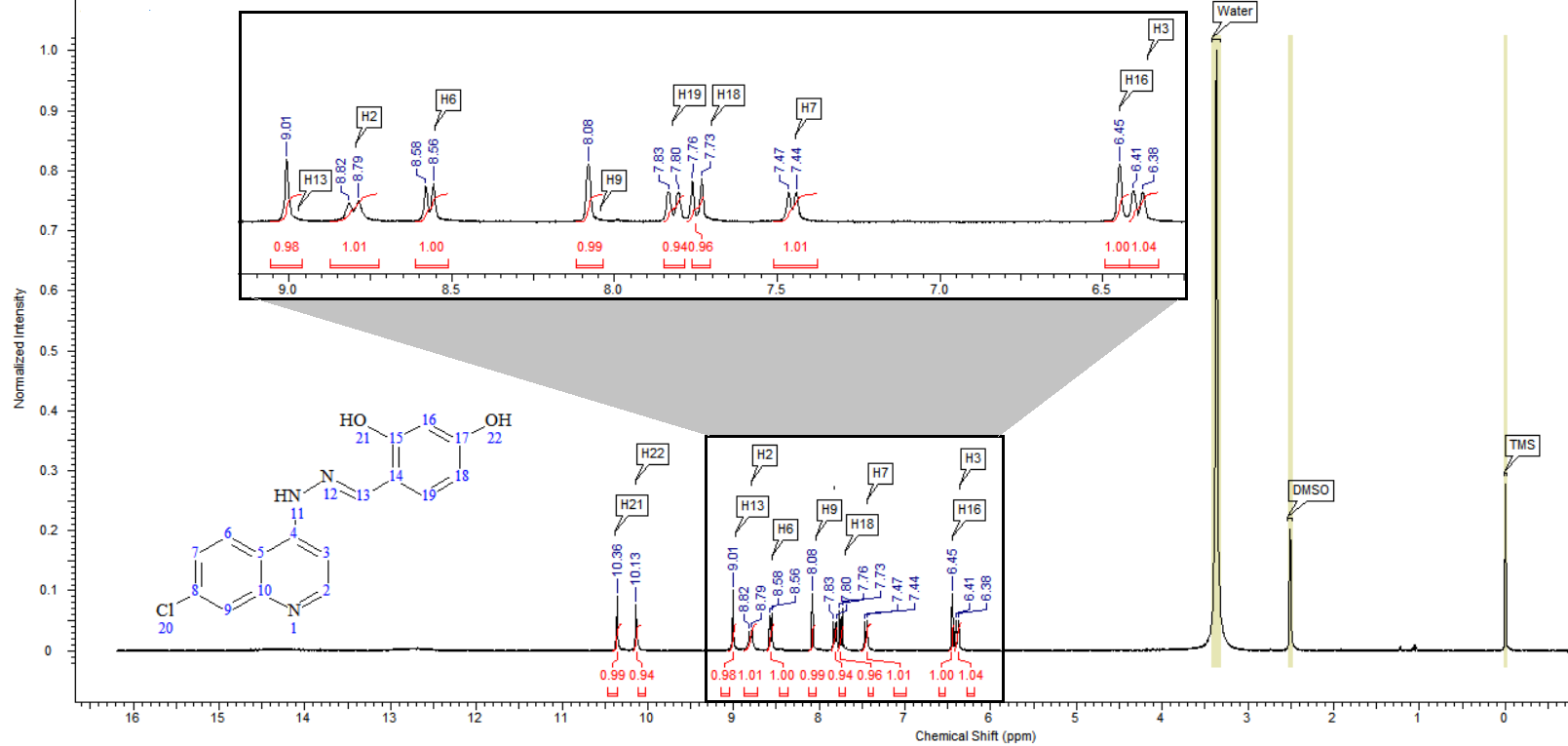
**

**Figure S26**. IV of compound **GPQF-8Q14**

**
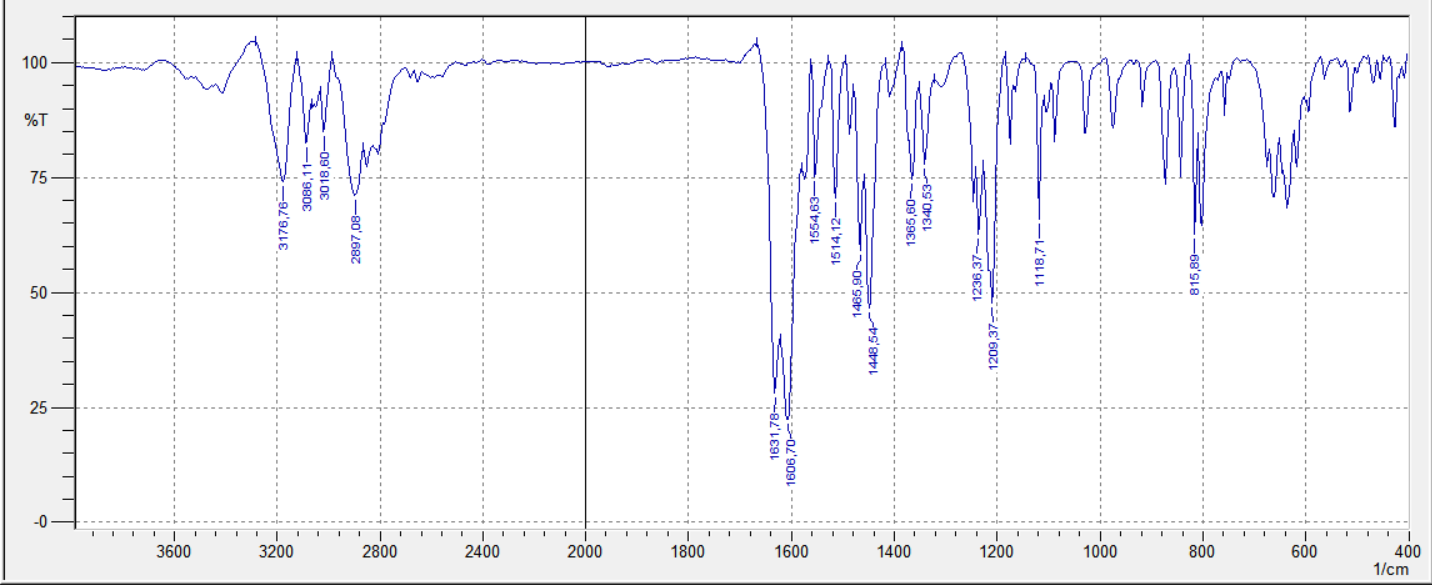
**

**Figure S27.** ^1^H-NMR of compound **GPQF-8Q16**

**
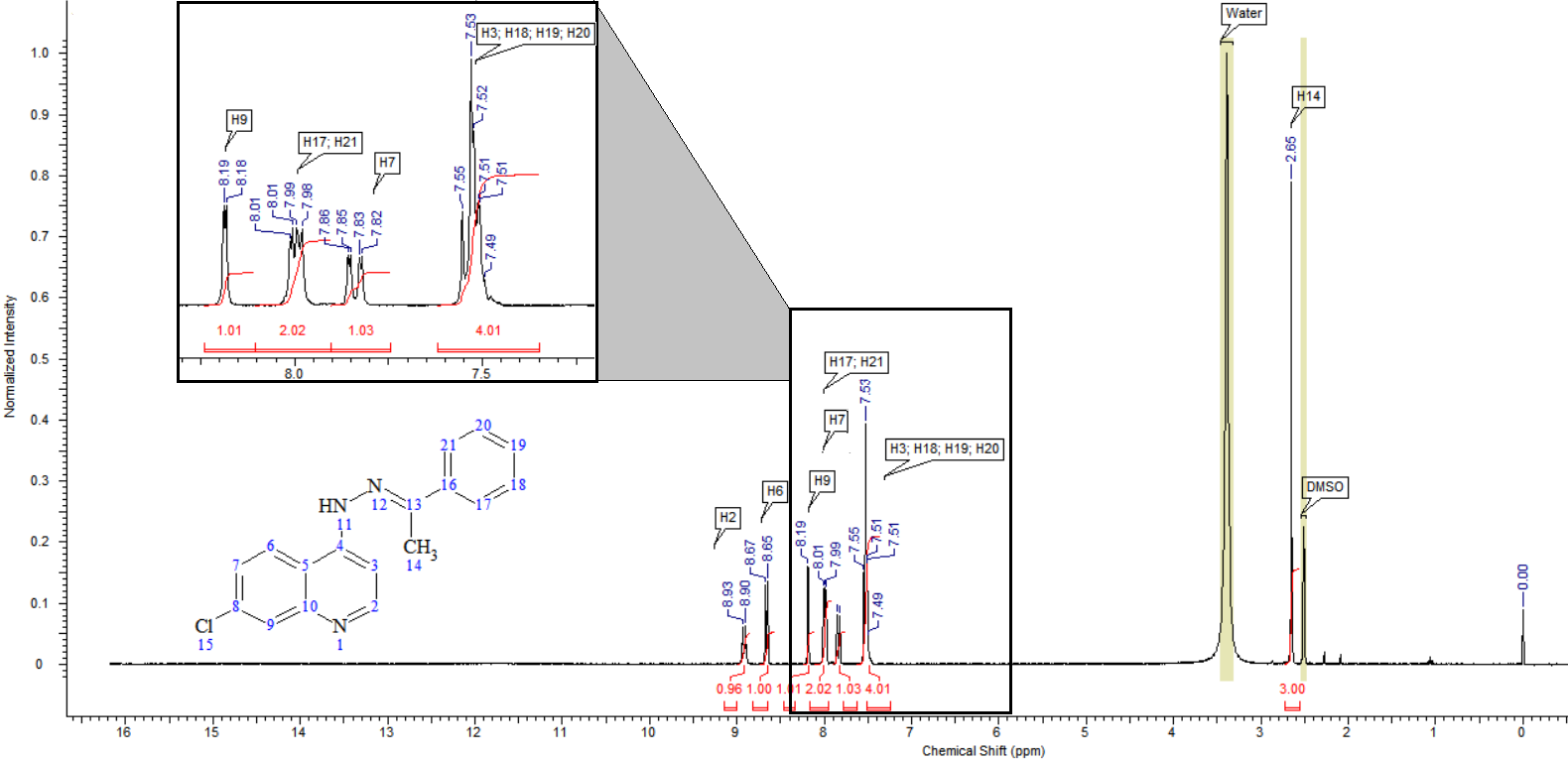
**

**Figure S28**. IV of compound **GPQF-8Q16**

**
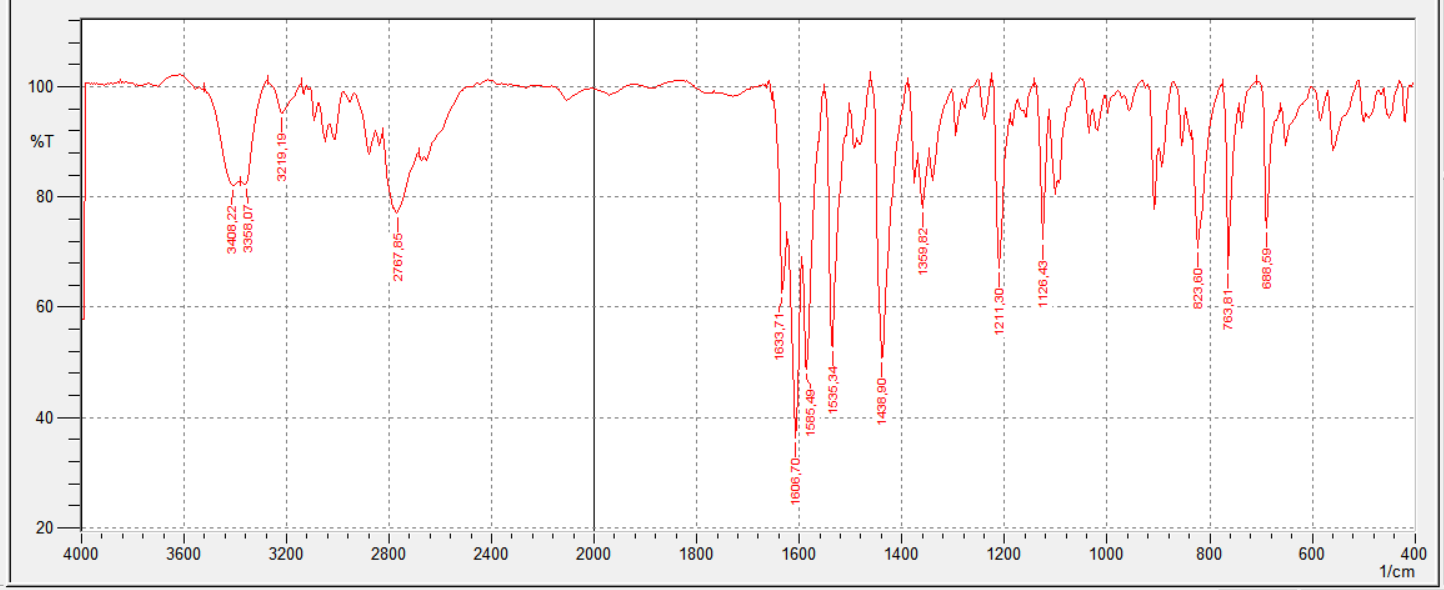
**

**Figure S29.** ^1^H-NMR of compound **GPQF-8Q17**

**
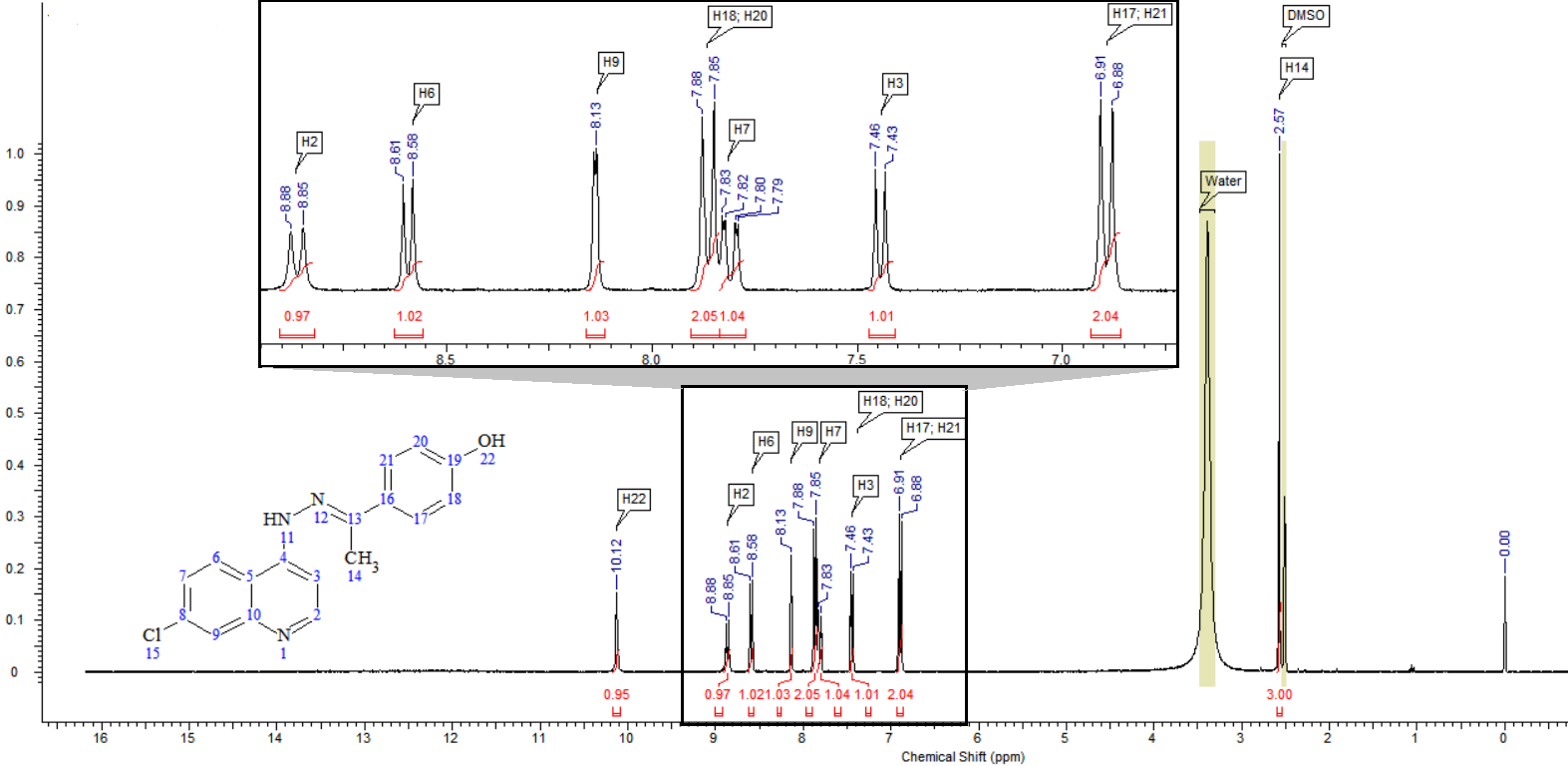
**

**Figure S30**. IV of compound **GPQF-8Q17**

**
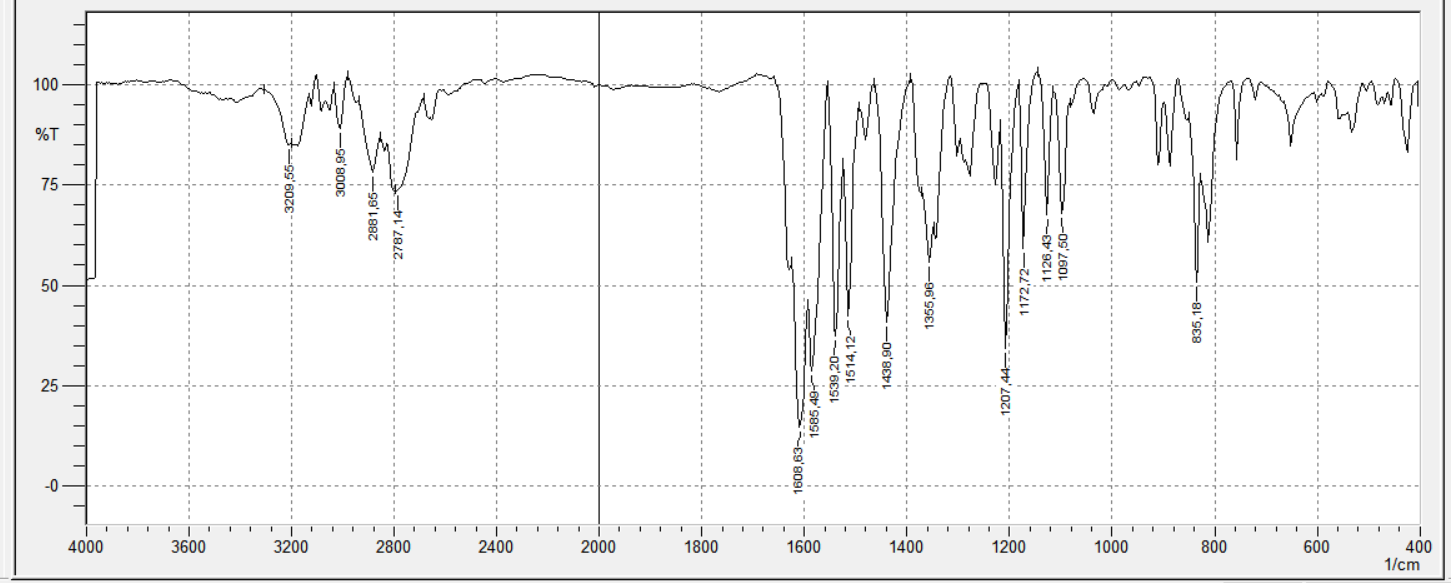
**

**Figure S31.** ^1^H-NMR of compound **GPQF-8Q18**

**
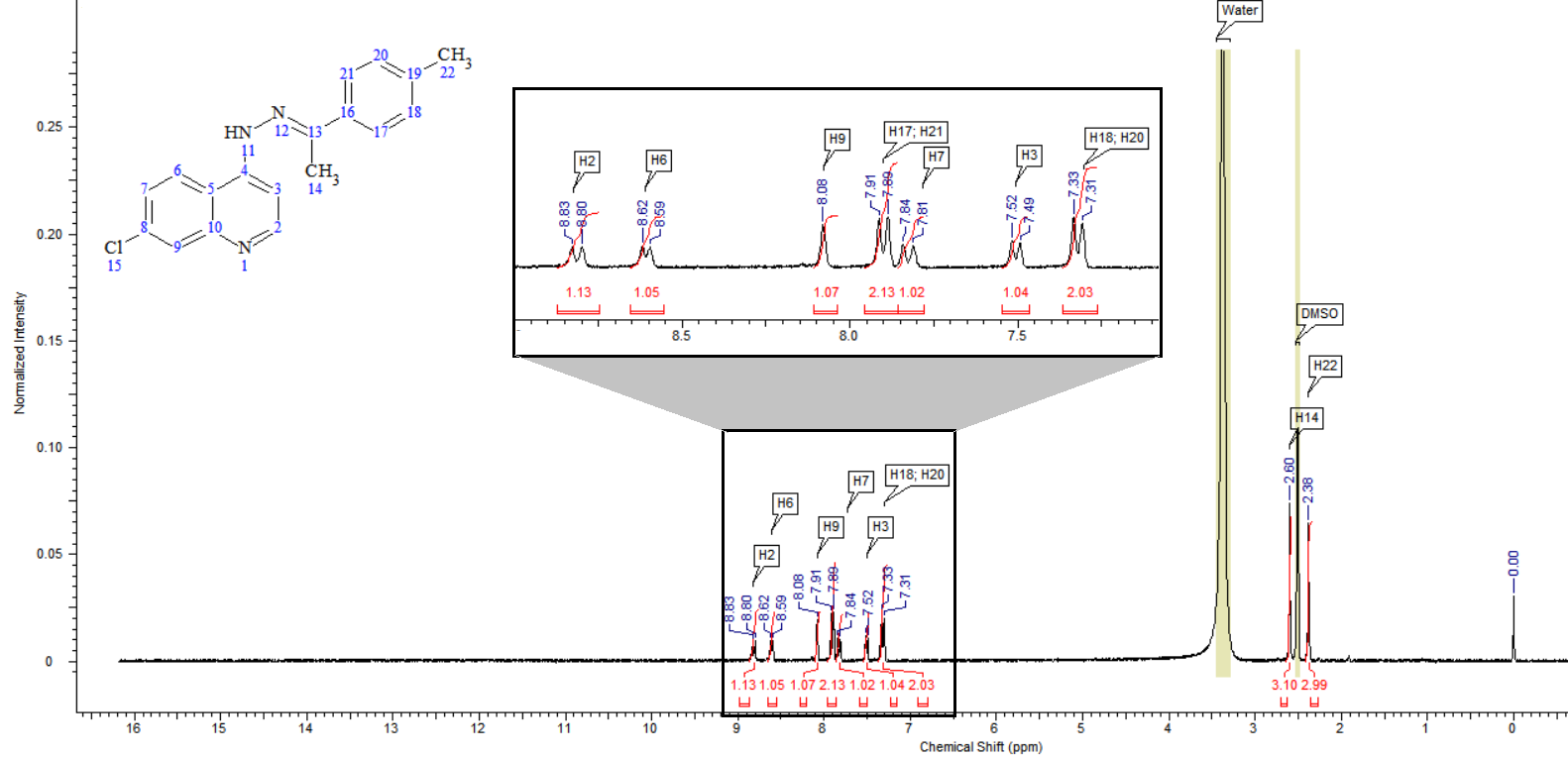
**

**Figure S32**. IV of compound **GPQF-8Q18**

**
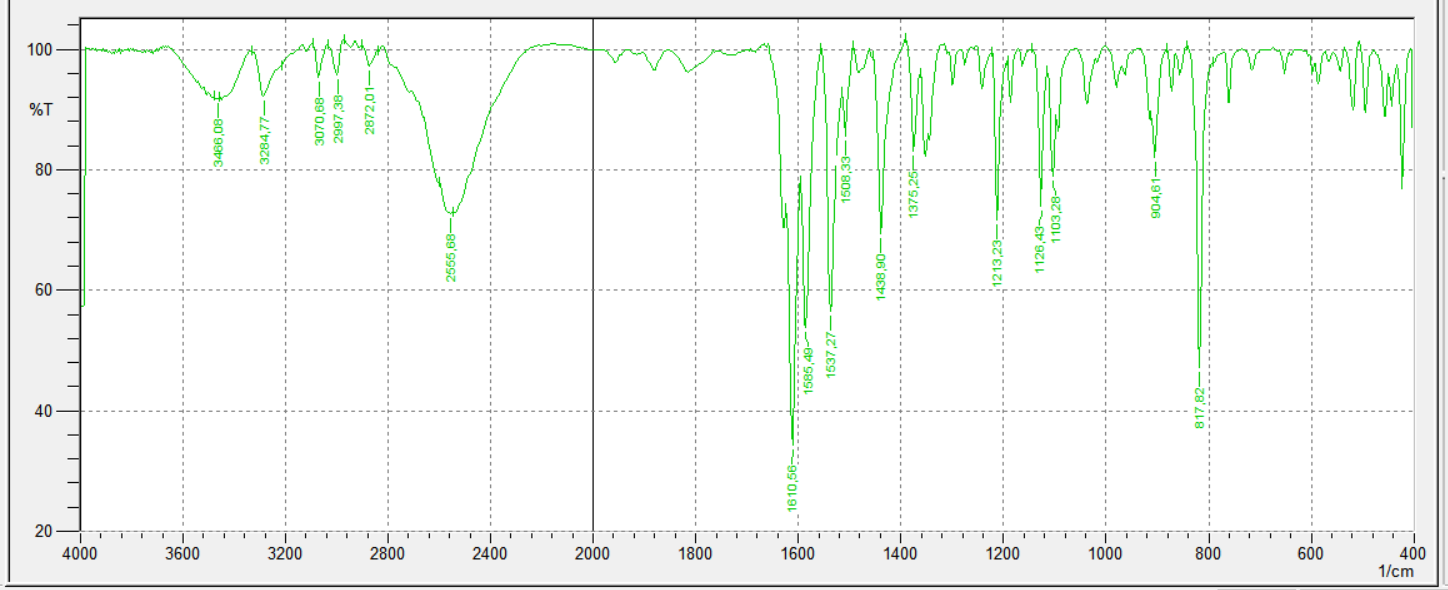
**

**Figure S33.** ^1^H-NMR of compound **GPQF-8Q19**

**
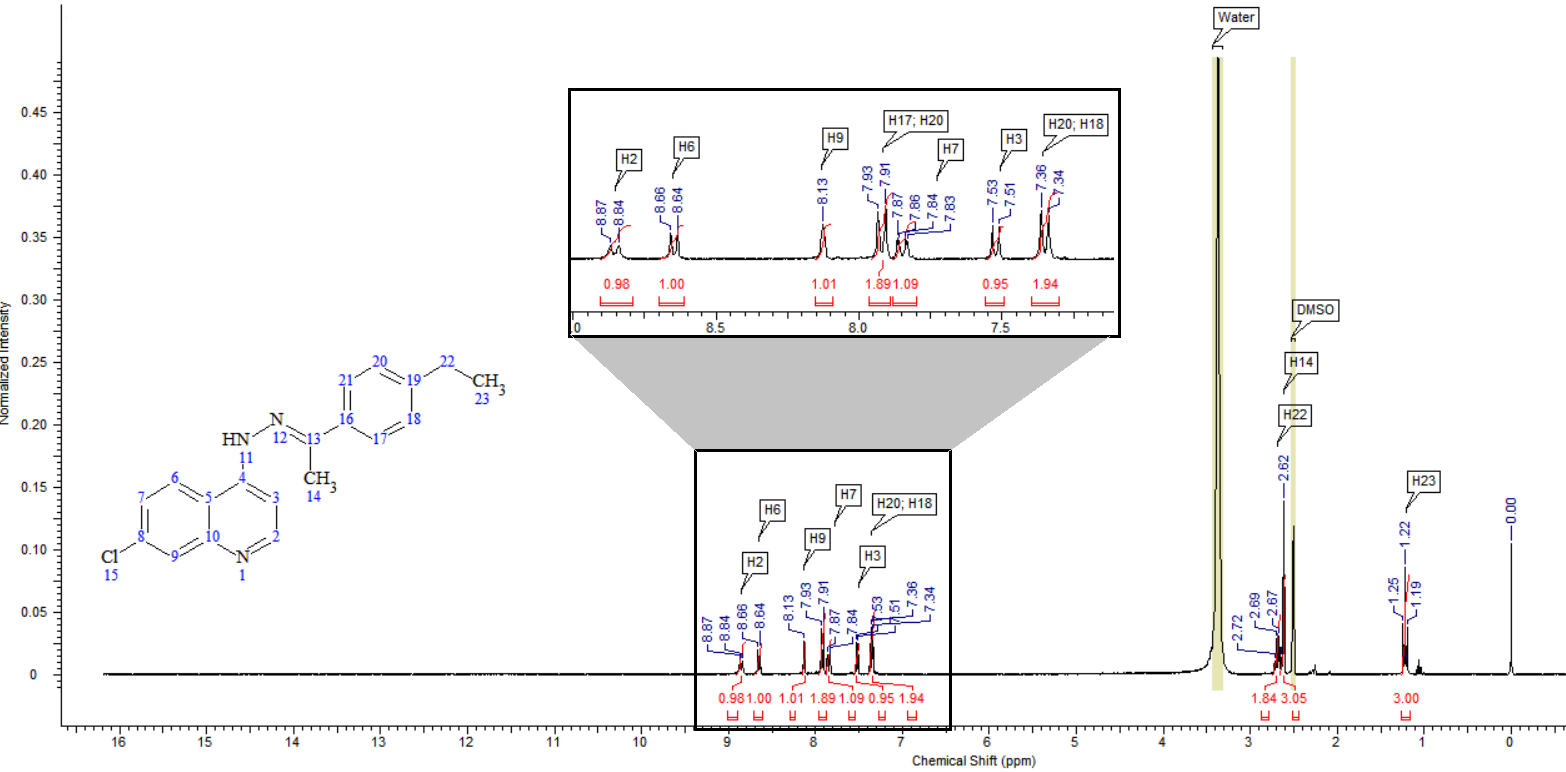
**

**Figure S34**. IV of compound **GPQF-8Q19**

**
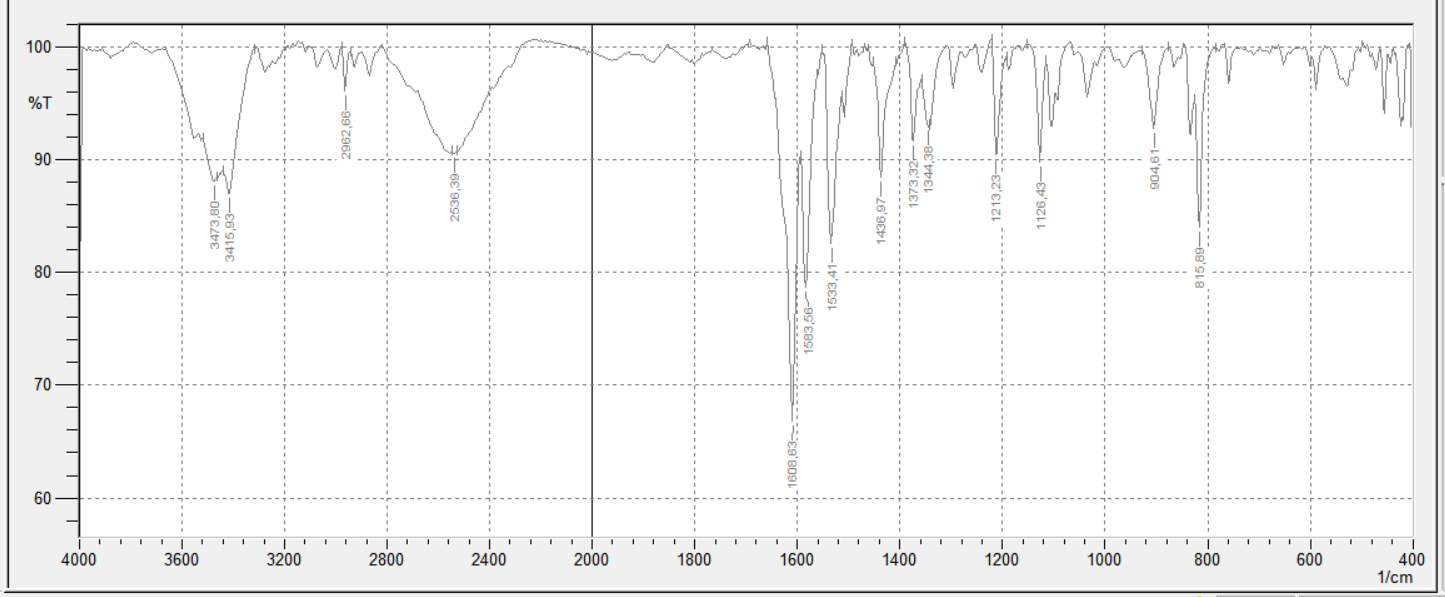
**

**Figure S35.** ^1^H-NMR of compound **GPQF-8Q20**

**
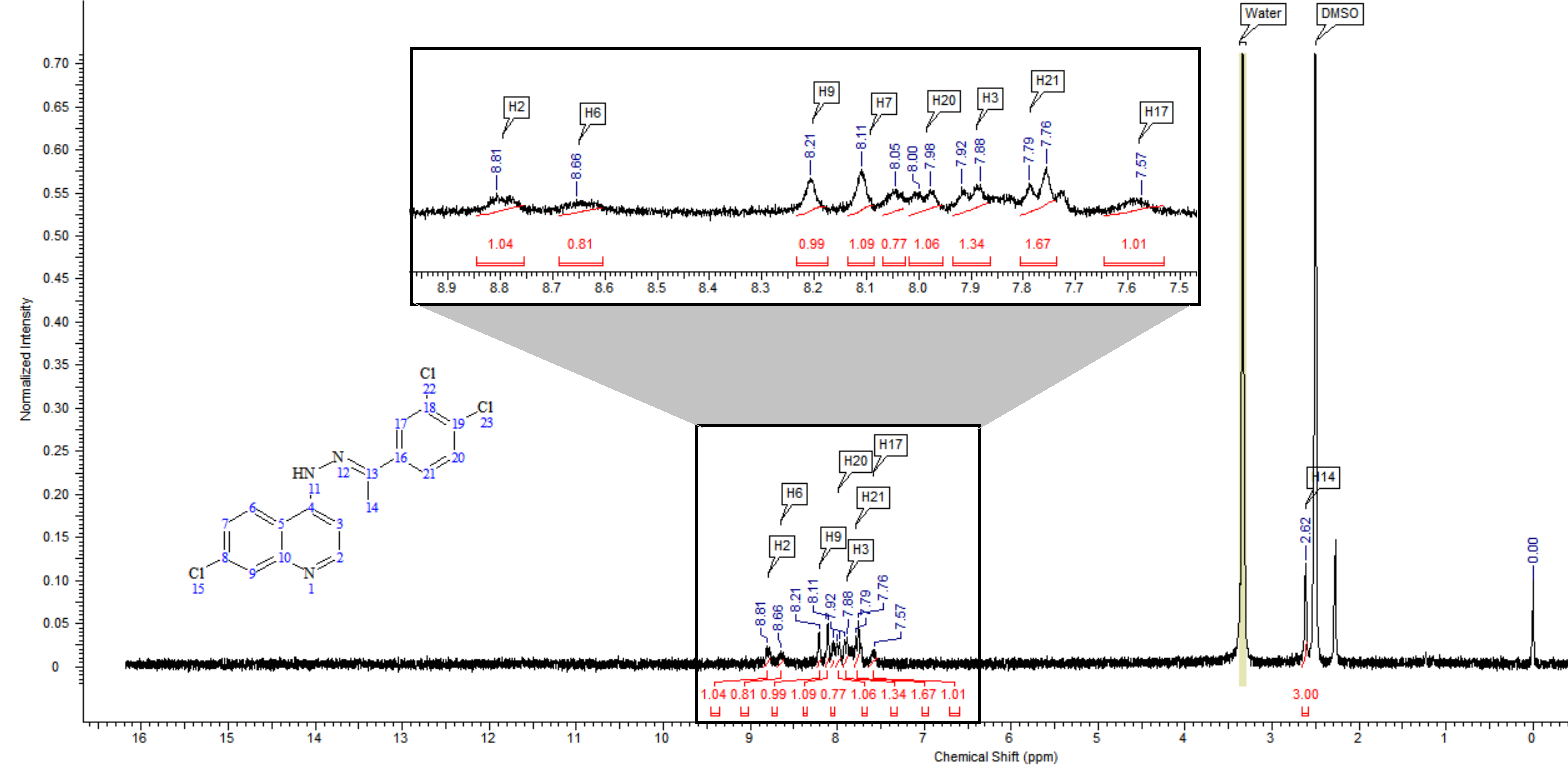
**

**Figure S36**. IV of compound **GPQF-8Q20**

**
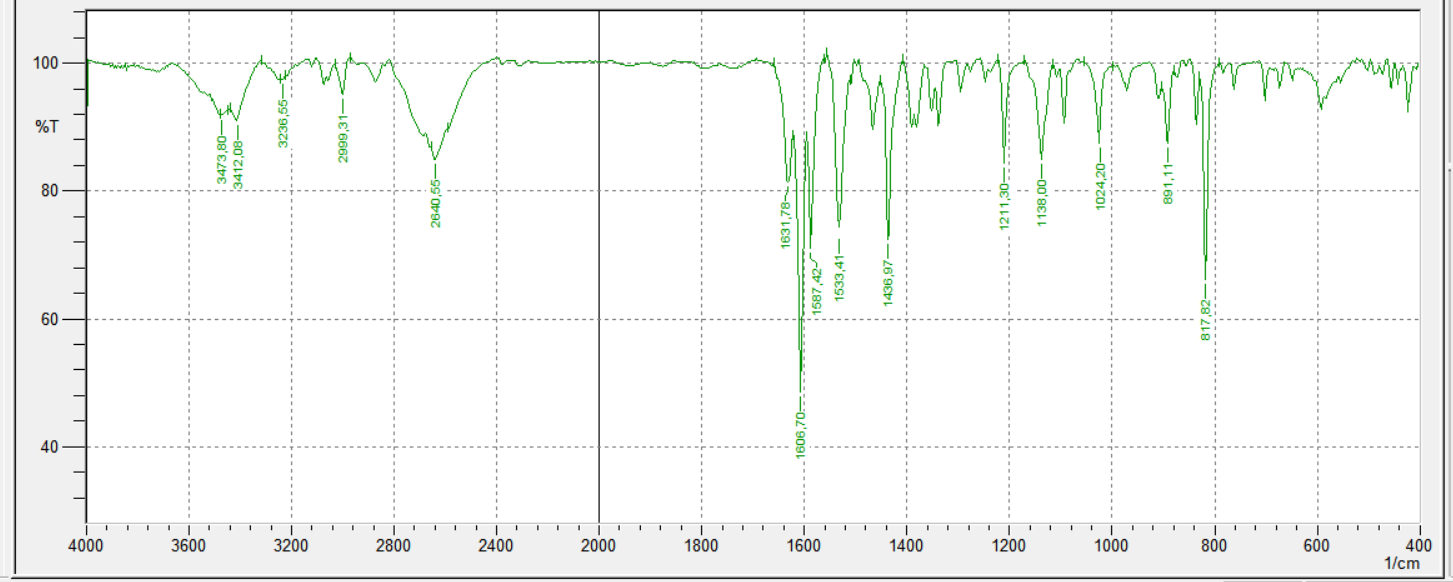
**

**Figure S37.** ^1^H-NMR of compound **GPQF-8Q21**

**
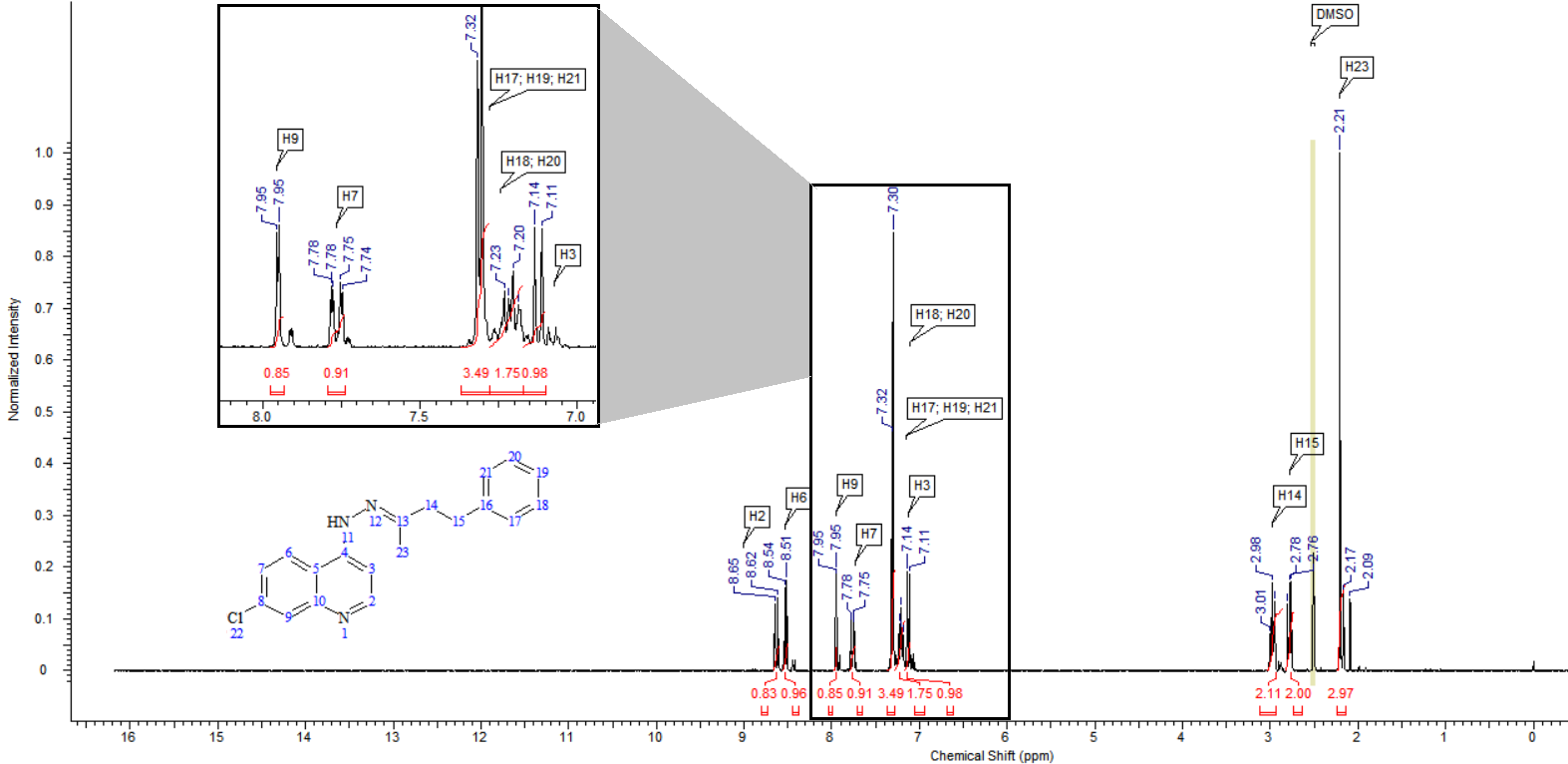
**

**Figure S38**. IV of compound **GPQF-8Q21**

**
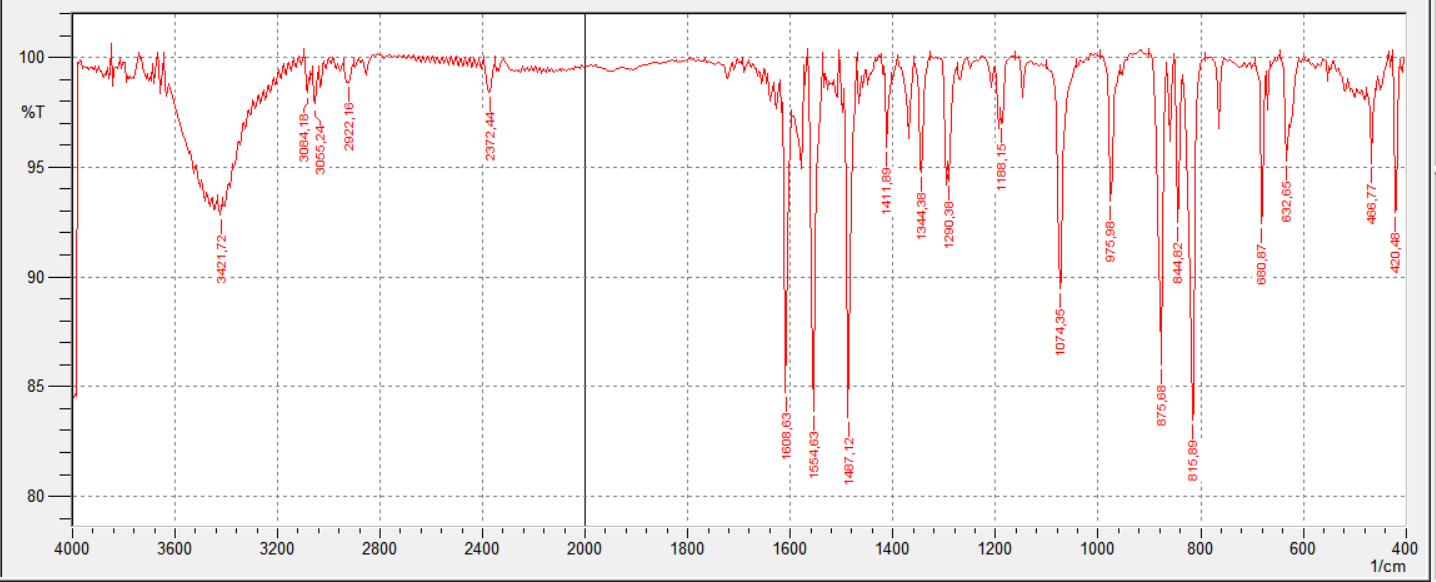
**

**Figure S39.** ^1^H-NMR of compound **GPQF-8Q22**

**
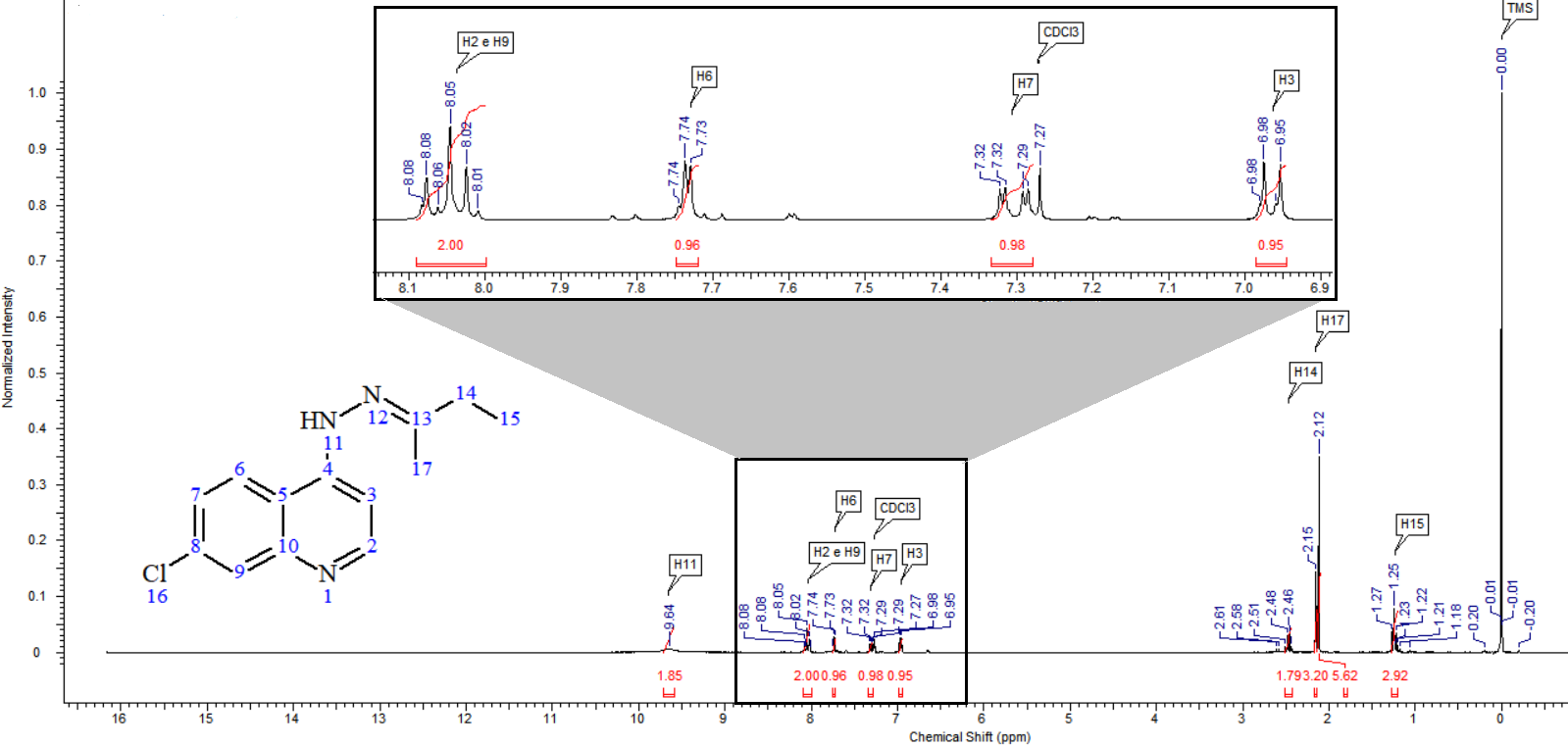
**

**Figure S40**. IV of compound **GPQF-8Q22**

**
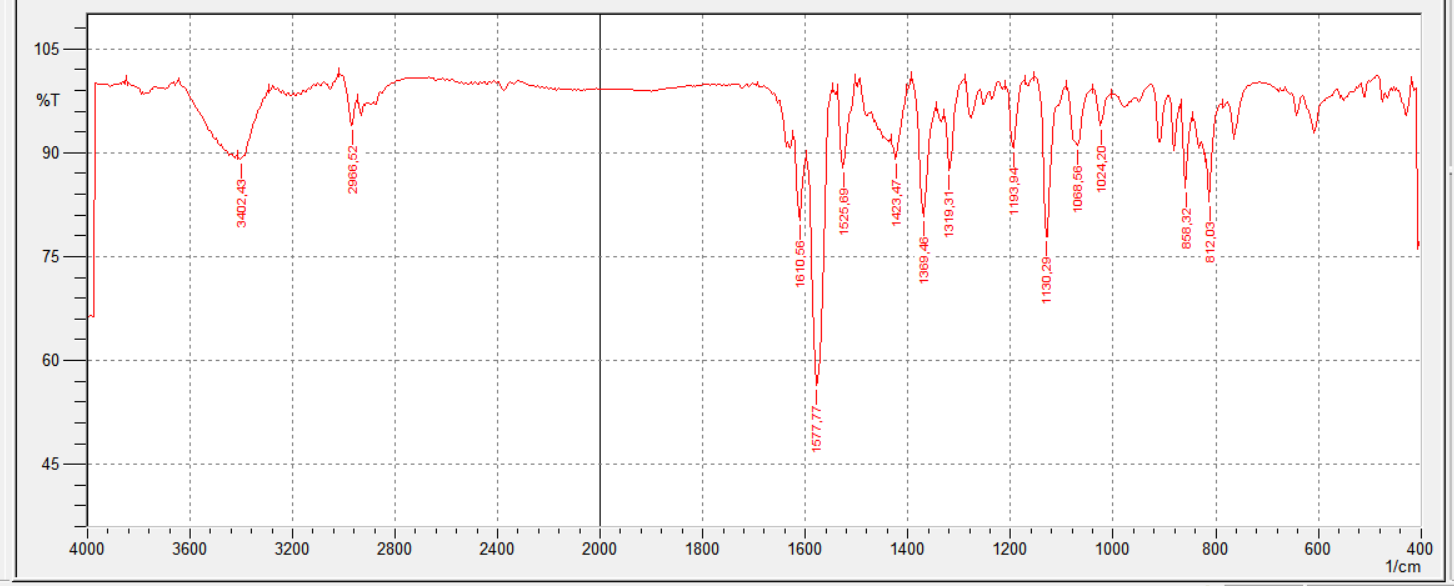
**

**Figure S41.** ^1^H-NMR of compound **GPQF-8Q23**

**
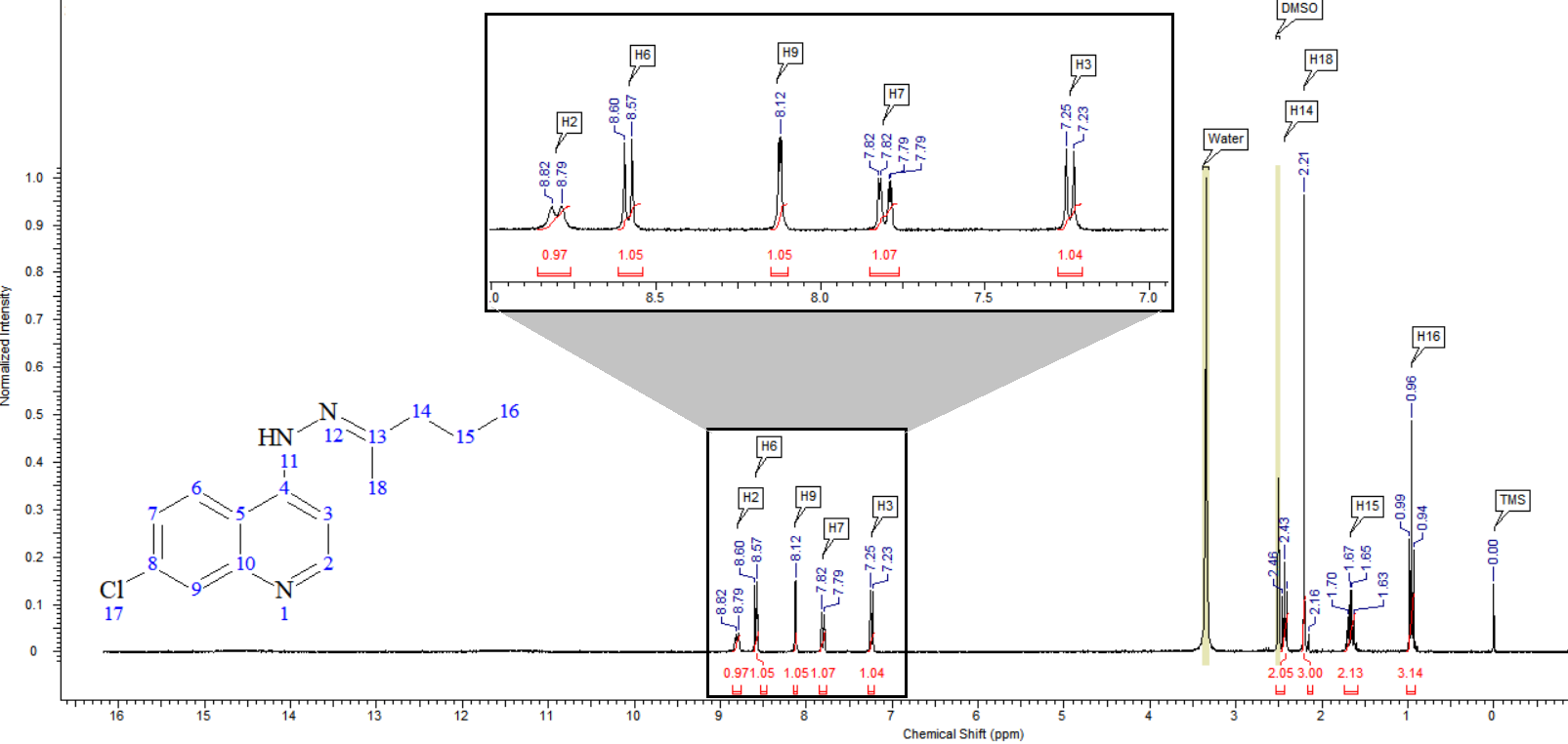
**

**Figure S42**. IV of compound **GPQF-8Q23**

**
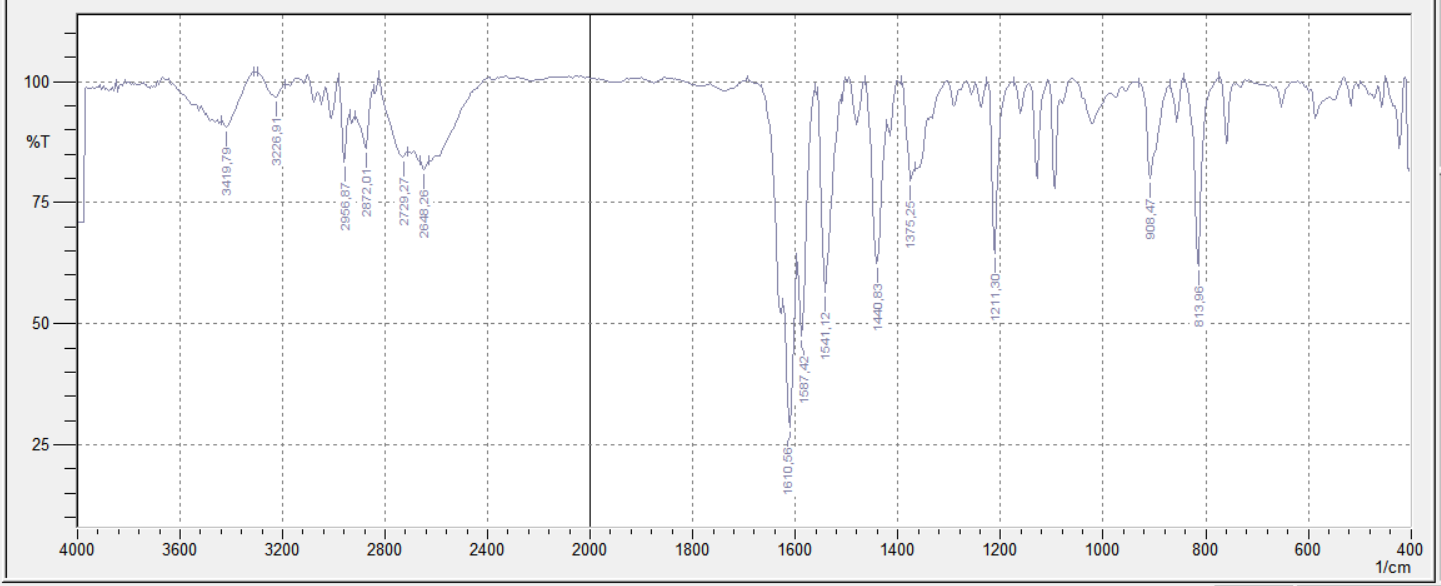
**

**Figure S43.** ^1^H-NMR of compound **GPQF-8Q25**

**
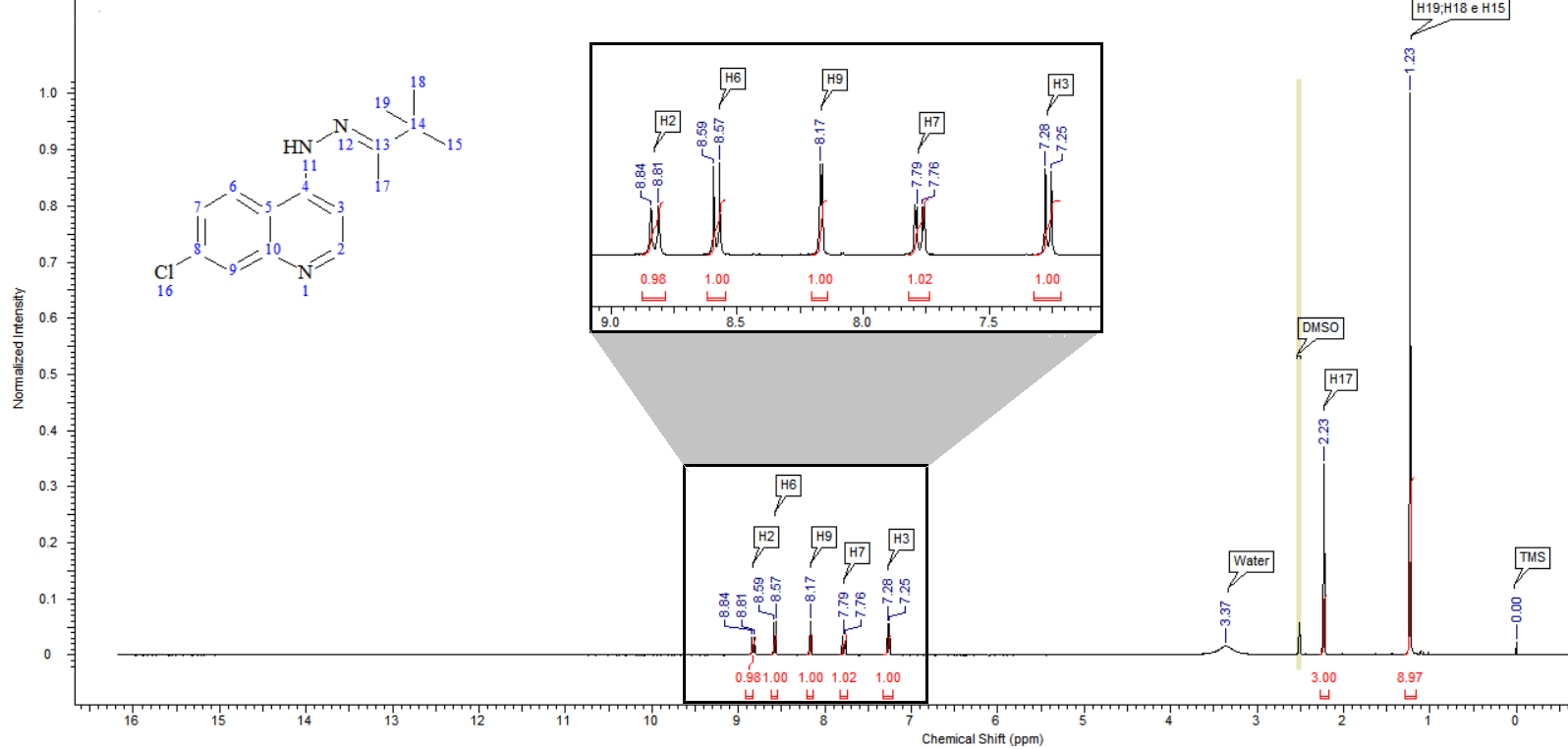
**

**Figure S44**. IV of compound **GPQF-8Q25**

**
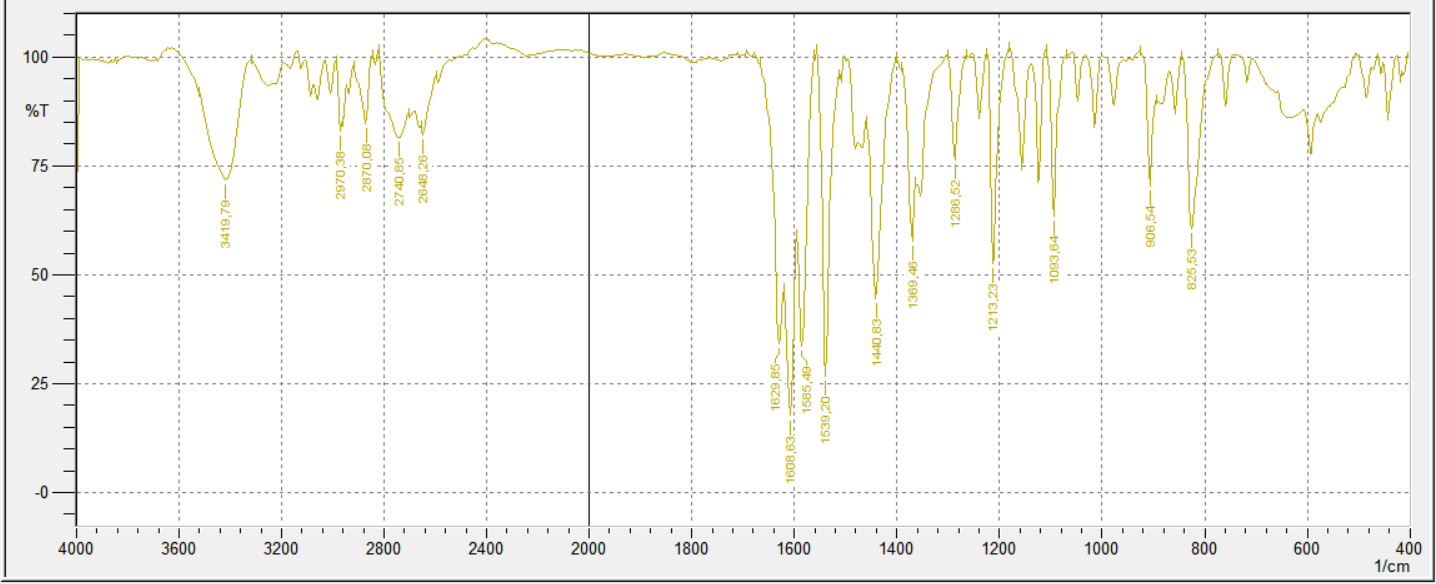
**

**Figure S45.** ^1^H-NMR of compound **GPQF-8Q26**

**
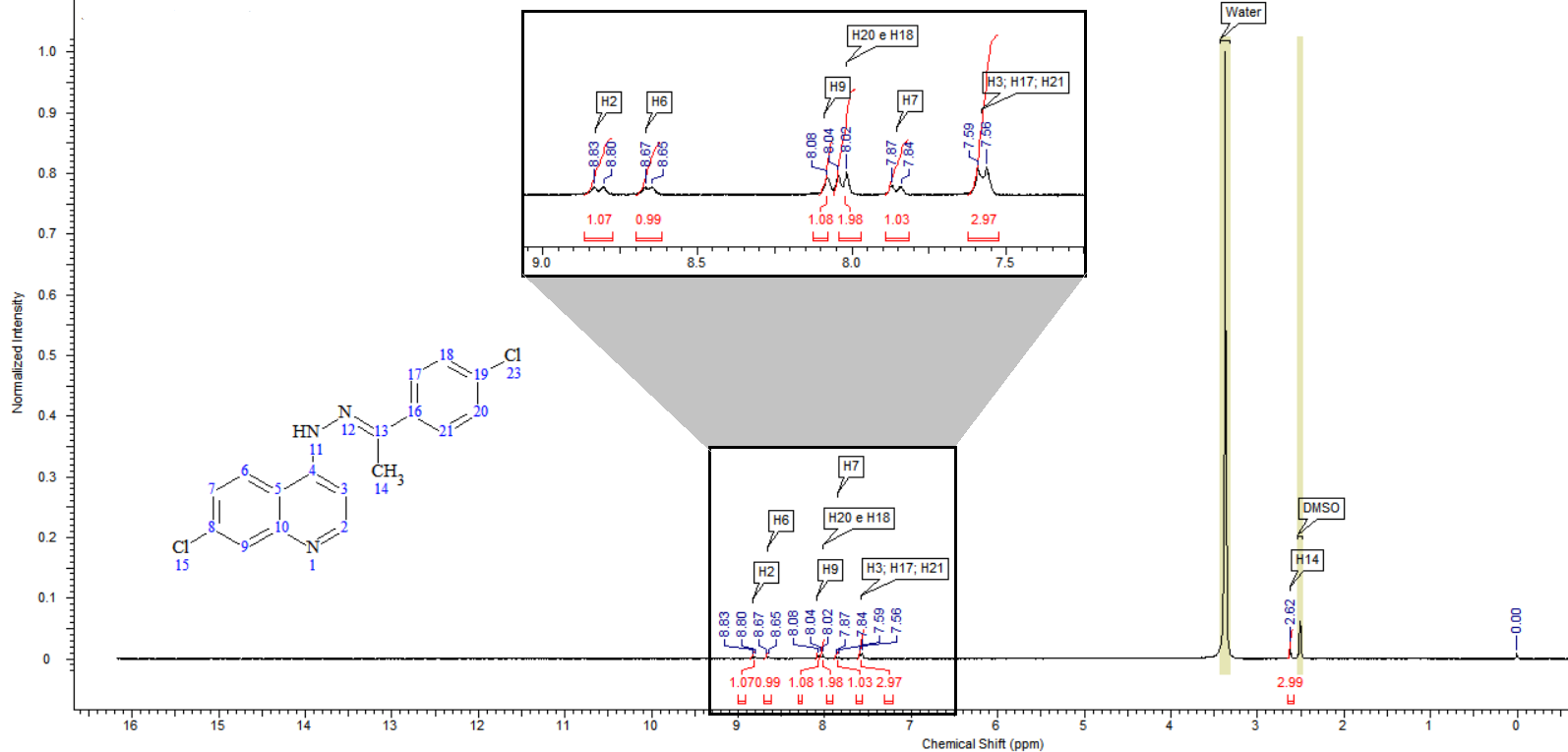
**

**Figure S46**. IV of compound **GPQF-8Q26**

**
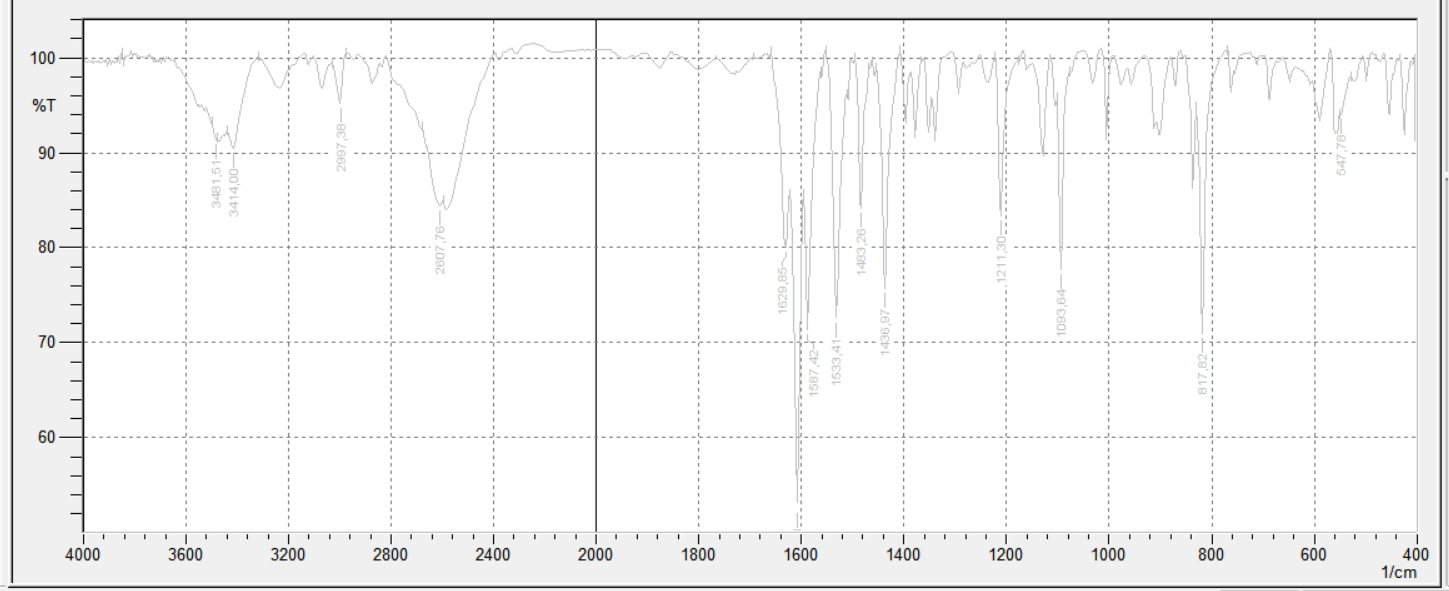
**
